# Fibroblast activation protein α activatable theranostic pro-photosensitizer for accurate tumor imaging and highly-specific photodynamic therapy

**DOI:** 10.7150/thno.70308

**Published:** 2022-05-01

**Authors:** Yong Luo, Zishan Zeng, Ting Shan, Xiaoyu Xu, Jie Chen, Yuanfeng He, Tao Zhang, Zeqian Huang, Guihong Chai, Yanjuan Huang, Yanfang Zhao, Chunshun Zhao

**Affiliations:** 1 School of Pharmaceutical Sciences, Sun Yat-sen University, Guangzhou, 510006, P. R. China.; 2 Key Laboratory of Structure-Based Drug Design and Discovery, Ministry of Education, Shenyang Pharmaceutical University, 103 Wenhua Road, Shenhe District, Shenyang, 110016, P. R. China.

**Keywords:** Fibroblast activation protein α, activatable photosensitizer, oxidative stress amplification, tumor imaging, photodynamic therapy

## Abstract

The development of activatable photosensitizers (aPSs) responding to tumor-specific biomarkers for precision photodynamic therapy (PDT) is urgently required. Due to the unique proteolytic activity and highly restricted distribution of tumor-specific enzymes, enzyme activatable photosensitizers display superior selectivity.

**Methods:** Herein, a series of novel Fibroblast Activation Protein α (FAPα) activatable theranostic pro-photosensitizers were designed by conjugating the different *N*-terminal blocked FAPα-sensitive dipeptide substrates with a clinical PS, methylene blue (MB), through a self-immolative linker, which resulting in the annihilation of the photoactivity (fluorescence and phototoxicity). The best FAPα-responsive pro-photosensitizer was screened out through hydrolytic efficiency and blood stability. Subsequently, a series of *in vitro* and *in vivo* experiments were carried out to investigate the FAPα responsiveness and enhanced PDT efficacy.

**Results:** The pro-photosensitizers could be effectively activated by tumor-specific FAPα in the tumor sites. After response to FAPα, the “uncaged” MB can recover its fluorescence and phototoxicity for tumor imaging and cytotoxic singlet oxygen (^1^O_2_) generation, eventually achieving accurate imaging-guided PDT. Simultaneously, the generated azaquinone methide (AQM) could serve as a glutathione (GSH) scavenger to rapidly and irreversibly weaken intracellular antioxidant capacity, realizing synergistic oxidative stress amplification and enhanced PDT effect.

**Conclusion:** This novel FAPα activatable theranostic pro-photosensitizers allow for accurate tumor imaging and admirable PDT efficacy with minimal systemic side effects, offering great potential in clinical precision antitumor application.

## Introduction

Cancer is a major public health threat worldwide with morbidity and mortality increasing year by year 1. Delayed diagnosis and drug resistance of existing treatments are two major factors leading to poor prognosis of cancer patients, which highlights the urgent need for timely and effective tumors theranostics.

Photodynamic therapy (PDT), combining photosensitizers (PSs), light, and molecular oxygen, has gained extensive attention as a noninvasive and reliable cancer treatment modality 2, 3. During PDT process, PSs can generate toxic reactive oxygen species (ROS), especially singlet oxygen (^1^O_2_) upon light irradiation to induce cell death 4-7. More importantly, the inherent fluorescence of PSs offers an opportunity for bioimaging 8-10. However, currently available “always-on” PSs are lack of tumor specificity, which cannot adjust themselves for the selective imaging and tumor cells killing, thereby inducing the treatment-related side effects in adjacent normal tissues as well as prolonged skin photosensitivity 11, 12. Moreover, on account of the high concentration of glutathione (GSH) in tumor cells, the ^1^O_2_ produced during PDT is subjected to rapid chemical reduction, thereby reducing the effectiveness of PDT 13-15. Therefore, it is extremely urgent to develop tumor-specific activatable photosensitizers (aPSs) for accurate tumor imaging and enhanced PDT efficacy with minimal side effects.

Up to now, the aPSs have been designed mainly based on the mechanisms of fluorescence resonance energy transfer (FRET), aggregation-induced emission (AIE), photo-induced electron transfer (PeT) or self-quenching of PSs 16-22. However, the photoactivities of most aPSs always cannot be annihilated completely. It is still of great significance for the development of aPSs with almost non-photoactivities before activation to enhance the specificity of tumor imaging and PDT. Meanwhile, the previously reported aPSs can be activated by a variety of triggers, such as GSH, ROS, acidic environment, etc 23-29. Notably, enzyme activatable aPSs show higher tumor selectivity owing to strict biodistribution of tumor-specific enzymes 8, 30-33. Particularly, fibroblast activation protein α (FAPα) is a type II transmembrane serine protease of the dipeptidyl peptidase (DPP) subfamily, which is overexpressed in over 90% of solid tumors, but generally absent in adjacent normal tissues 34, 35. In addition, FAPα is distinguished from other proteases in the DPP subfamily, as its activity is restricted to substrates containing the *N*-terminal blocked Gly-Pro dipeptide. The highly tumor restricted distribution and specific proteolytic activity of FAPα make it a very attractive target for precision cancer diagnosis and treatment. Previously studies have been verified that FAPα could exclusively hydrolyze substrates containing the *N*-terminal benzyloxycarbonyl blocked Gly-Pro (Z-GP) 36-39. However, the Z-GP dipeptide is not suitable for all molecules, owing to the different spatial structure when fluorogenic substrates or drugs are covalently linked to the targeted peptides, which may lead to obvious distinctions in the hydrolysis of FAPα 40-44. Consequently, it is necessary to further explore the correlation between the enzymolysis efficiency and structure of artificial substrates, and screen the most suitable *N*-terminal blocked structure.

**Scheme 1:**
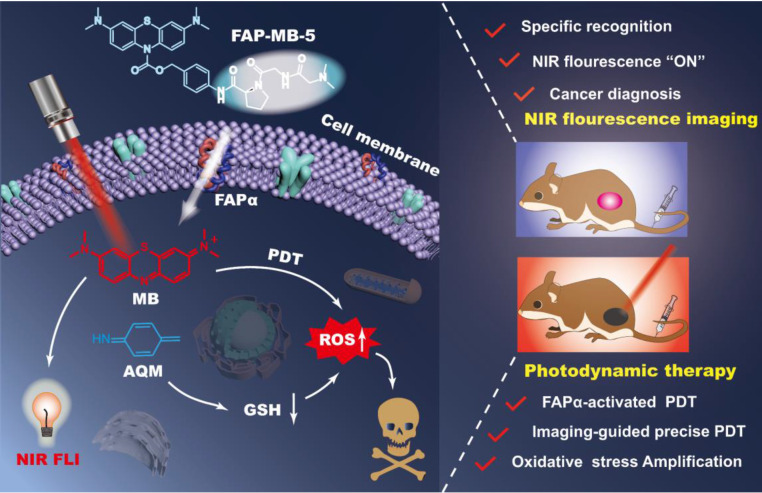
Illustration of FAPα activatable “one for all” theranostic pro-photosensitizer for *in vivo* accurate cancer diagnosis and high-specific (imaging-guided/enhanced) PDT.

Herein, we designed and constructed a series of novel FAPα activatable theranostic pro-photosensitizers (FAP-MB-1~10) by conjugating the different *N*-terminal blocked FAPα-sensitive dipeptide substrates with a clinical PS, methylene blue (MB), through a self-immolative *p*-aminobenzyl alcohol linker. Due to the formation of carbamate bond at nitrogen of MB phenothiazine ring and thus the destruction of π-conjugation in backbone, these pro-photosensitizers containing “caged” MB initially have no photoactivities (“turn-off” state) 45-48. Among these FAPα activatable theranostic pro-photosensitizers, FAP-MB-5 with *N,N*-dimethylglycine blocked *N*-terminal structure could be specifically and effectively activated by recombinant human FAPα (rhFAPα) and FAPα-expressed tumor tissues. The hydrolysis of amide bond between the dipeptide substrate and self-immolative linker in FAP-MB-5 initiates the formation of intermediate aniline, followed by 1,6-elimination, and eventually releasing free MB (“turn-on” state) to recover the photoactivity (fluorescence and phototoxicity). Notably, the “turn-on” of near-infrared (NIR) fluorescence of MB not only allows for specific tumor imaging, but also realizes the accurate guidance for PDT. Additionally, the synchronously generated azaquinone methide (AQM) could effectively deplete GSH 49, 50, suppressing the antioxidant ability of tumor cells for further amplified oxidative stress and reinforced PDT efficacy. *In vivo* studies verified that FAP-MB-5 could effectively reach the tumor sites to display selective activation for accurate tumor imaging and admirable PDT efficacy with minimal systemic side effects. Therefore, this novel FAPα activatable theranostic pro-photosensitizer with excellent biosafety displayed great potential for clinical precise tumor diagnosis and highly-specific PDT (**Scheme 1**).

## Results and Discussion

### Design and synthesis of FAP-MB-X

**Figure 1:**
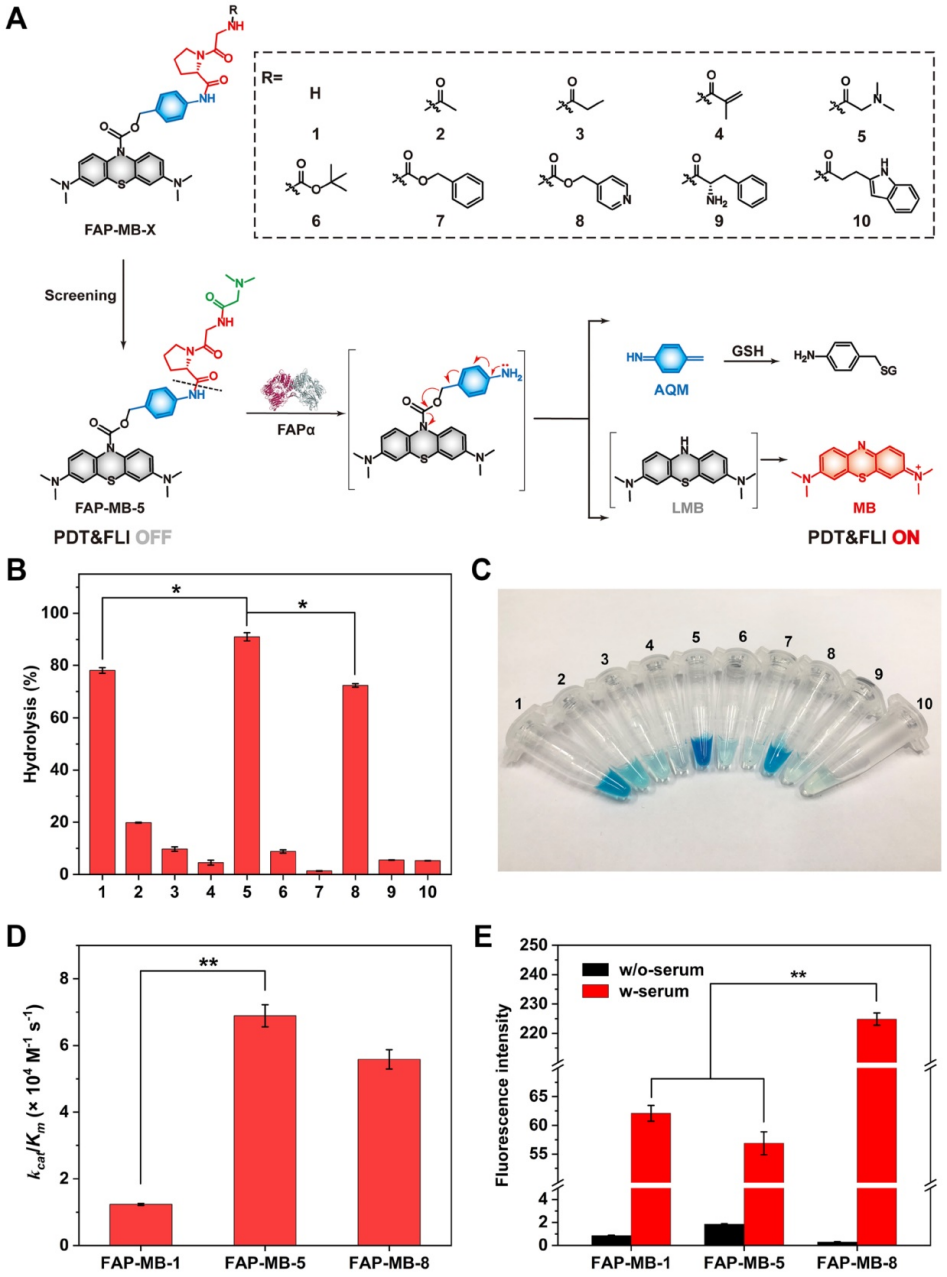
(**A**) The design strategy and mechanism of FAPα activatable theranostic photosensitizer FAP-MB-1~10. (**B**) Evaluation of the hydrolytic efficiency of pro-photosensitizer FAP-MB-1~10 by rhFAPα *via* HPLC. (**C**) The photograph of pro-photosensitizer FAP-MB-1~10 after hydrolysis by rhFAPα. (**D**) The catalytic efficiency of rhFAPα towards FAP-MB-1, FAP-MB-5 and FAP-MB-8. (**E**) The fluorescence intensity of FAP-MB-1, FAP-MB-5 and FAP-MB-8 incubated with or without serum. Results are described as mean ± SD, *n* = 3. *p < 0.05, **p < 0.01, and ***p < 0.001.

FAPα has been verified to exhibit efficient endopeptidase activity, which is highly specific for the X-Gly-Pro pattern (where X stands for an acyl terminus or a peptide) 51-53. Meanwhile, various *N*-terminal blocked groups (X) may have different steric hindrances for molecules, so that replacing diverse blocked groups may have different response to FAPα. To explore the most suitable *N*-terminal blocked structure, a series of novel FAPα activatable pro-photosensitizers (FAP-MB-1~10) by conjugating the different *N*-terminal blocked FAPα-sensitive dipeptide substrates with MB, through a PABA self-immolative linker were designed and constructed **(Figure 1A)**. To create the novel theranostic aPSs, a synthetic route of the target compounds was developed (**Scheme S1**). Taking MB as the staring material, the active intermediate A was obtained by reduction and acylation reaction. The intermediate B was prepared *via* condensation of *N*-terminal Boc-protected dipeptides (Gly-Pro) with *p*-aminobenzyl alcohol. The intermediate C was obtained by reaction of active intermediate A with intermediate B, followed by removing Boc under the trifluoroacetic acid to obtain intermediate D, which was then condensed or substituted with different *N*-terminal blocked groups to obtain the FAP-MB-1~10. The final compounds and important intermediates were characterized by LC-MS, HPLC,^ 1^H-NMR and ^13^C-NMR. (**Figures S21-S64**).

### Evaluation of the hydrolytic efficiency of pro-photosensitizer

In order to screen out the best FAPα-responsive pro-photosensitizer from candidate compound 1-10, we detected whether these ten pro-photosensitizers could be hydrolyzed into active ingredient MB by rhFAPα using HPLC along with color changes of the hydrolysate solution (**Figure 1B, 1C and Figure S1**). Based on the standard curve of MB, there was negligible hydrolysis in compound 7 incubated with rhFAPα, which proved that Z-GP dipeptide was not suitable for all molecules. Only less than 20% of compound 2, 3, 4, 6, 9, 10 were hydrolyzed to MB, indicating that compound 2, 3, 4, 6, 7, 9, 10 were not suitable substrates for FAPα. By contrast, 80% of compound 1 and 8 (referred to FAP-MB-1 and FAP-MB-8) were hydrolyzed while the hydrolytic efficiency of compound 5 (also referred to FAP-MB-5) was greater than 90%, demonstrating compound 1, 5, 8 were probably optimum substrates towards FAPα. To further evaluate the binding affinity between FAPα and pro-photosensitizers, the Michaelis-Menten constants (*K*_m_), a significant metric for enzymatic reaction kinetics, of rhFAPα towards FAP-MB-1, FAP-MB-5, FAP-MB-8 were calculated to be 368, 78.1 and 26.4 μM, respectively (**Figure S2**). Moreover, the catalytic efficiency (*k*_cat_/*K*_m_) of rhFAPα towards FAP-MB-1, FAP-MB-5, FAP-MB-8 were calculated to be 1.23 × 10^4^, 6.89 × 10^4^ and 5.58 × 10^4^ M^-1^ s^-1^ (**Figure 1D**). The catalytic efficiency of FAP-MB-5 was approximately 5.6-fold higher than that of FAP-MB-1, and 1.2-fold higher than that of FAP-MB-8, indicating FAP-MB-5 was the most suitable substrate towards FAPα among these candidate compounds. Additionally, to evaluate stability of pro-photosensitizers in blood, FAP-MB-1, FAP-MB-5, FAP-MB-8 were incubated with serum collected from mice, respectively. As shown in **Figure 1E** and **Figure S3**, FAP-MB-1, FAP-MB-5 and FAP-MB-8 displayed negligible fluorescence in PBS at 674 nm, which verified conjugation at the nitrogen of phenothiazine ring of MB could thoroughly eliminate its fluorescence. Both FAP-MB-1 and FAP-MB-5 possessed favorable blood stability when incubation with serum. Nevertheless, FAP-MB-8 was readily cleaved by bioactive substance and released MB in blood, which would cause undesired systemic toxicity and incapable cancer diagnosis *in vivo*. Therefore, according to above screening results, FAP-MB-5 was selected as optimal FAPα activatable theranostic photosensitizer for accurate cancer diagnosis and PDT in following studies.

### The optical properties of FAP-MB-5 towards FAPα

**Figure 2:**
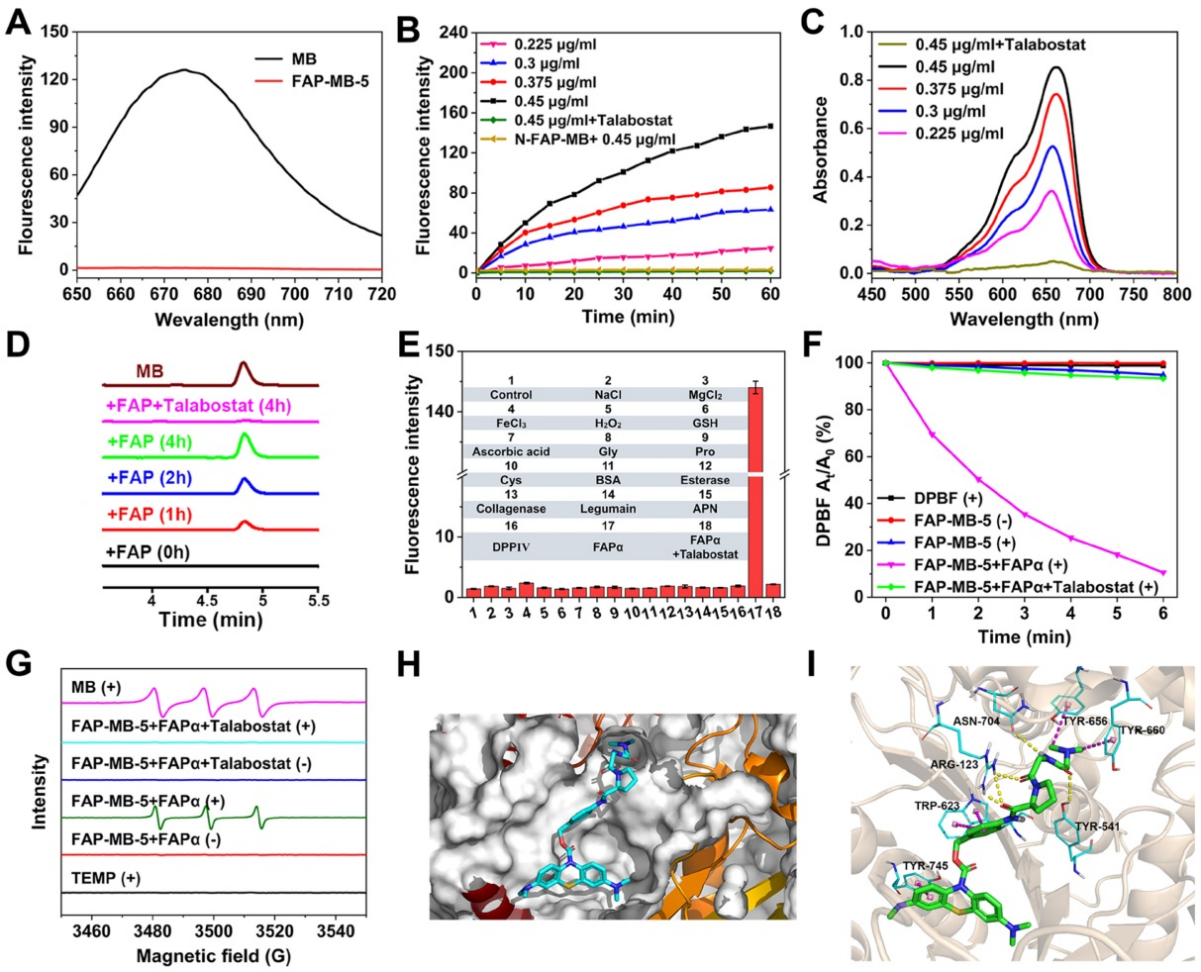
(**A**) Optical characterization of fluorescence emission of FAP-MB-5 and MB in MeOH. (**B**) Fluorescence intensity of FAP-MB-5 (200 μM) pre-incubated with or without talabostat (100 μM) for 1 h towards various concentration of rhFAPα (0.225, 0.3, 0.375, 0.45 μg/mL) over time. Excitation: 630 nm. (**C**) UV-vis absorption spectra change of FAP-MB-5 (500 μM) towards various concentration of rhFAPα (0.225, 0.3, 0.375, 0.45 μg/mL). As inhibition group, rhFAPα (0.45 μg/mL) was pre-incubated with talabostat (100 μM) for 1 h, and then incubated with FAP-MB-5. (**D**) HPLC chromatogram of rhFAPα-mediated hydrolysis of FAP-MB-5 over time. As inhibition group, rhFAPα was pre-incubated with talabostat (100 μM) for 1 h. (**E**) Fluorescence response of FAP-MB-5 (200 μM) treated with the indicated protein (0.45 μg/mL unless otherwise specified), metal iron (50 μM) and other analytes (50 μM) for 1 h. 1, control; 2, Na^+^; 3, Mg^2+^; 4, Fe^3+^; 5, H_2_O_2_; 6, GSH; 7, ascorbic acid; 8, Gly; 9, Pro; 10, Cys; 11, BSA; 12, esterase; 13, collagenase; 14, Legumain; 15, APN; 16, DPPIV; 17, rhFAPα (0.225 μg/mL) pre-incubated with talabostat; 18, rhFAPα (0.225 μg/mL). (**F**) DPBF attenuation by ^1^O_2_ generation with different treatments in MeOH at 415 nm. (**G**) ESR spectra of different reaction systems with TEMP as the spin trap. (**H**) Docking analysis of the interactions of FAP-MB-5 with FAPα. (**I**) Detailed interactions between FAP-MB-5 and FAPα in a three-dimensional view. (+) and (-) refer to the treatment with or without irradiation, respectively. Results are described as mean ± SD, *n* = 3.

Inspired by the design of FAP-MB-5, N-FAP-MB, which cannot be effectively cleaved by FAPα, was synthesized as negative control following the synthesis procedures illustrated in (**Scheme S2**). Using FAPα inactive Gly-Gly dipeptide as linker substituted Gly-Pro dipeptide to obtain the N-FAP-MB (**Figure S65-S76**). MB exhibits a strong maximal absorption peak at 665 nm and fluorescence emission at 674 nm (λ_ex_ = 630 nm). As expected, either FAP-MB-5 or N-FAP-MB displays extremely weak absorbance and fluorescence at 665 nm and 674 nm, respectively (**Figure 2A and Figure S4-S6**). When conjugated at the nitrogen of phenothiazine ring, MB is converted into the reduced form of MB, which would interrupt intramolecular electron transfer and block the π-conjugated system, resulting in thorough fluorescence and absorption elimination with photosensitivity quenching. In the presence of FAPα, the amide bond between the dipeptide substrate and self-immolative linker of FAP-MB-5 was cleaved, followed by 1,6-elimination to release leucomethylene blue (LMB) that would be further oxidized to MB, resulting in the significant recovery of fluorescence at 674 nm, which was important for tumor tissue imaging and PDT *in vivo*. In comparison, due to the lack of recognition substrate, N-FAP-MB could not be cleaved and release free MB, resulting in inapparent fluorescence change. When FAPα was pretreated with FAPα inhibitor talabostat before incubated with FAP-MB-5, the recovery of fluorescence was greatly inhibited, indicating the fluorescence “turn-on” response of FAP-MB-5 was specifically aroused by enzymatic cleavage of FAPα. Furthermore, the fluorescence intensity at 674 nm was gradually increased with time-dependent manner (**Figure 2B**). Meanwhile, the reaction contained FAP-MB-5 and rhFAPα (0.45 μg/mL) pre-incubated with talabostat showed imperceptible change of fluorescence intensity with increased treatment time. FAPα concentration-dependent liberation was observed *via* UV-vis absorption and fluorescence spectra, respectively (**Figure 2B and 2C**). By detecting a newly emerged product peak at 665 nm, HPLC analyses further verified FAP-MB-5 could efficiently release MB in response to rhFAPα. As shown in **Figure 2D**, the peak of the elution product with a retention time of 4.8 min increased over incubation time, which was corresponding to the reference compound MB. Whereas, N-FAP-MB was rarely hydrolyzed after rhFAPα incubation, indicating the FAPα non-responsive property of N-FAP-MB (**Figure S7**). To investigate the specific hydrolysis of pro-photosensitizer at tumor site which exhibited higher FAPα expression, the fluorescence changes of FAP-MB-5 incubated with major organ homogenates were recorded at 674 nm. As shown in **Figure S8**, compared with other organ homogenates, FAP-MB-5 treated with tumorous homogenates possessed prominent fluorescence intensity enhancement within 4 h of incubation, suggesting FAP-MB-5 preferred to be activated and release MB in tumor site, which would be beneficial for achieving precise cancer diagnosis and high-specificity PDT.

Moreover, considering the complex physiological environment* in vivo*, it is significant to investigate the performance of FAP-MB-5 towards biological relevant analytes (**Figure 2E**). Obviously, FAP-MB-5 displayed high selectivity towards rhFAPα which was unaffected by other biological species, including important metal ions (Na^+^, Mg^2+^, Fe^3+^), bioactive small molecules (H_2_O_2_, GSH, ascorbic acid, Gly, Pro, Cys), and related proteins and enzymes (bovine serum albumin (BSA), dipeptidyl peptidase IV (DPPIV), aminopeptidase N (APN), legumain, esterase, collagenase), demonstrating the satisfactory specificity of FAP-MB-5 for recognition towards FAPα. In addition, the photosensitivity recovery owing to FAPα-mediated FAP-MB-5 activation was assessed by diphenylisobenzofuran (DPBF) oxidation, with the decrease of absorption intensity at 410 nm. As shown in **Figure 2F**, FAP-MB-5 rapidly generated plentiful ^1^O_2_ in the presence of rhFAPα under irradiation. Nevertheless, no significant absorption change was observed when talabostat inhibited the activity of rhFAPα, attesting the FAPα-activated generation of photosensitive MB for subsequent ROS generation. Therefore, this specific and distinguished responsiveness towards FAPα endowed FAP-MB-5 as a promising theranostic agent for cancer imaging and PDT. Additionally, ESR assay was performed to detect the generation of ^1^O_2_ of FAP-MB-5 under irradiation. 2,2,6,6-Tetramethylpiperidine (TEMP) was used as the capture agent to trap ^1^O_2_ in which the adduct displayed the representative “1:1:1” triplet signal. As show in **Figure 2G** and **Figure S9**, triplet ESR signals cannot be monitored in FAP-MB-5 (+) group and FAP-MB-5 + FAPα (-) group. In contrary, the signal of ^1^O_2_ could be detected in MB (+) and FAP-MB-5 + FAPα (+), suggesting FAP-MB-5 could be cleaved by FAPα to successfully release MB and generated ^1^O_2_ with irradiation. FAP-MB-5 could not effectively produce ^1^O_2_ with FAPα-mediated hydrolysis under irradiation condition in the presence of talabostat. These results demonstrated that FAP-MB-5 displayed the capacity of FAPα-specific activation for effective PDT.

To obtain the probable interaction and hydrolysis mechanism between FAP-MB-5 and FAPα, molecular docking analysis based on Schrödinger platform were conducted. The X-ray crystal structure of FAPα from the Protein Data Bank (PDB code 1Z68) was used 54. FAP-MB-5, when docked into the active site of FAPα featured by the Pro of FAP-MB-5, fits well with the cleavage site formed by one D (D703), two E (E203 and E204) and five Y (Y124, Y541, Y625, Y656, and Y660) 37, 40, which contributes to the amide bond between the dipeptide substrate and the self-immolative linker being cleavable by FAPα (**Figure 2H, 2I and Figure S10**). These results not only provided evidence that FAP-MB-5 is better tolerated but also demonstrated that FAP-MB-5 has stronger binding ability to FAPα.

### *In vitro* living cell imaging

**Figure 3:**
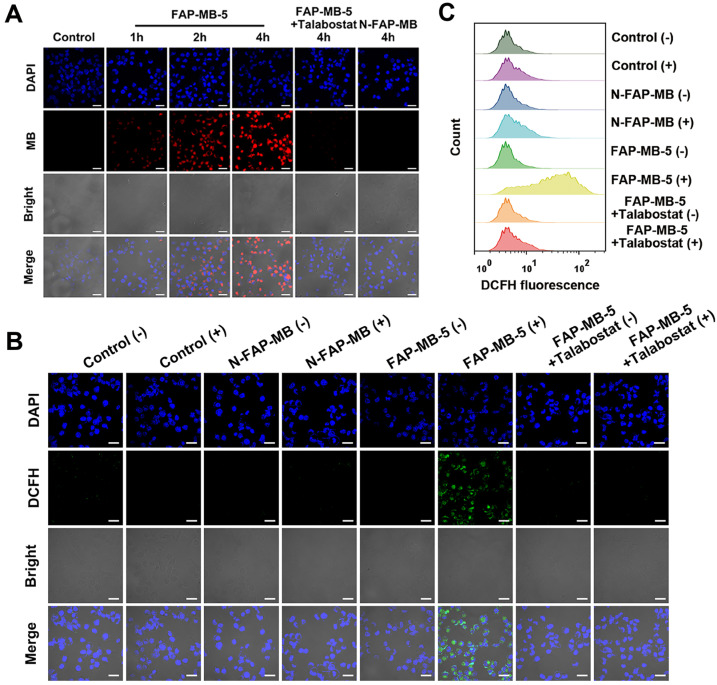
(**A**) CLSM images of Mia-paca-2 cells incubated with FAP-MB-5 (40 μg/mL) for 1 h, 2 h and 4 h. As inhibition group, the cells were pre-incubated with talabostat (50 μM) for 1 h, and then incubated FAP-MB-5 for 4 h. The blue fluorescence represents the cell nuclei stained with DAPI (excitation: 405 nm) and the red fluorescence represents MB liberated from FAP-MB-5 (excitation: 633 nm). Scale bar: 30 μm. Intracellular ROS generation of Mia-paca-2 cell treated with FAP-MB-5 (40 μg/mL) or N-FAP-MB (40 μg/mL) under dark or irradiation condition observed by CLSM (**B**) and flow cytometry analysis (**C**). The inhibition group was pre-incubated with talabostat for 1 h and treated with FAP-MB-5 with irradiation. Scale bar: 30 μm. (+) and (-) refer to the treatment with or without irradiation, respectively.

As FAPα could trigger fluorescent MB release from non-fluorescent FAP-MB-5, the concurrent fluorescence recovery in living cells was evaluated. Mia-paca-2 cells, the human pancreatic cancer cell line, were firstly verified high expression of FAPα *via* western blot (**Figure S11**) 55. Subsequently, the CLSM imaging illustrated in **Figure 3A** showed, FAP-MB-5 was efficaciously internalized into Mia-paca-2 cells with visible fluorescence signal appeared after incubation for 1 h. The fluorescence intensity became stronger at 4 h of treatment, declaring that FAP-MB-5 possessed the time-dependent cellular activation and fluorescent MB liberation. Furthermore, to confirm the intracellular fluorescence recovery was exactly caused by FAPα, Mia-paca-2 was pre-incubated with talabostat before being treated with FAP-MB-5. As expected, the fluorescence intensity was significantly decrease compared with that of without talabostat pre-incubated. Thus, we reasonably speculated that FAP-MB-5 could be efficiently and expeditiously activated by FAPα-positive cancer cells for specific cancer imaging and therapy.

### *In vitro* intracellular ROS production and GSH depletion

Due to the highly expressed FAPα of cancer cells, FAPα activatable FAP-MB-5 could be activated followed by 1,6-elimination to release photosensitizer MB and GSH consumption agent AQM. It is crucial for FAPα-activated photosensitizer to recover the ROS generation capability to perform PDT efficacy. ROS probe 2′,7′-dichlorofluorescein diacetate (DCFH) was employed to evaluate intracellular ROS generation *via* flow cytometry and CLSM, respectively. As illustrated in **Figure 3B**, inapparent green fluorescence was observed in control or N-FAP-MB group, which proved structure modification of MB at the nitrogen of phenothiazine ring would lose its inherent photoactivity for ROS generation. As expected, ROS-associated fluorescence intensity was dramatically higher in FAP-MB-5 group under irradiation. This result indicated that FAP-MB-5 could simultaneously generate photosensitizer MB to produce ROS under irradiation and GSH consumption agent AQM to restrain intracellular antioxidant capacity. Comparatively, FAP-MB-5 group in the present of talabostat only exhibited very weak green signal, which verified again that FAPα played a vital role in FAP-MB-5 activation and ROS generation to cause unbalanced redox homeostasis. In addition, flow cytometry analysis revealed consistent tendency with the results reflected by CLSM (**Figure 3C and Figure S12**).

**Figure 4:**
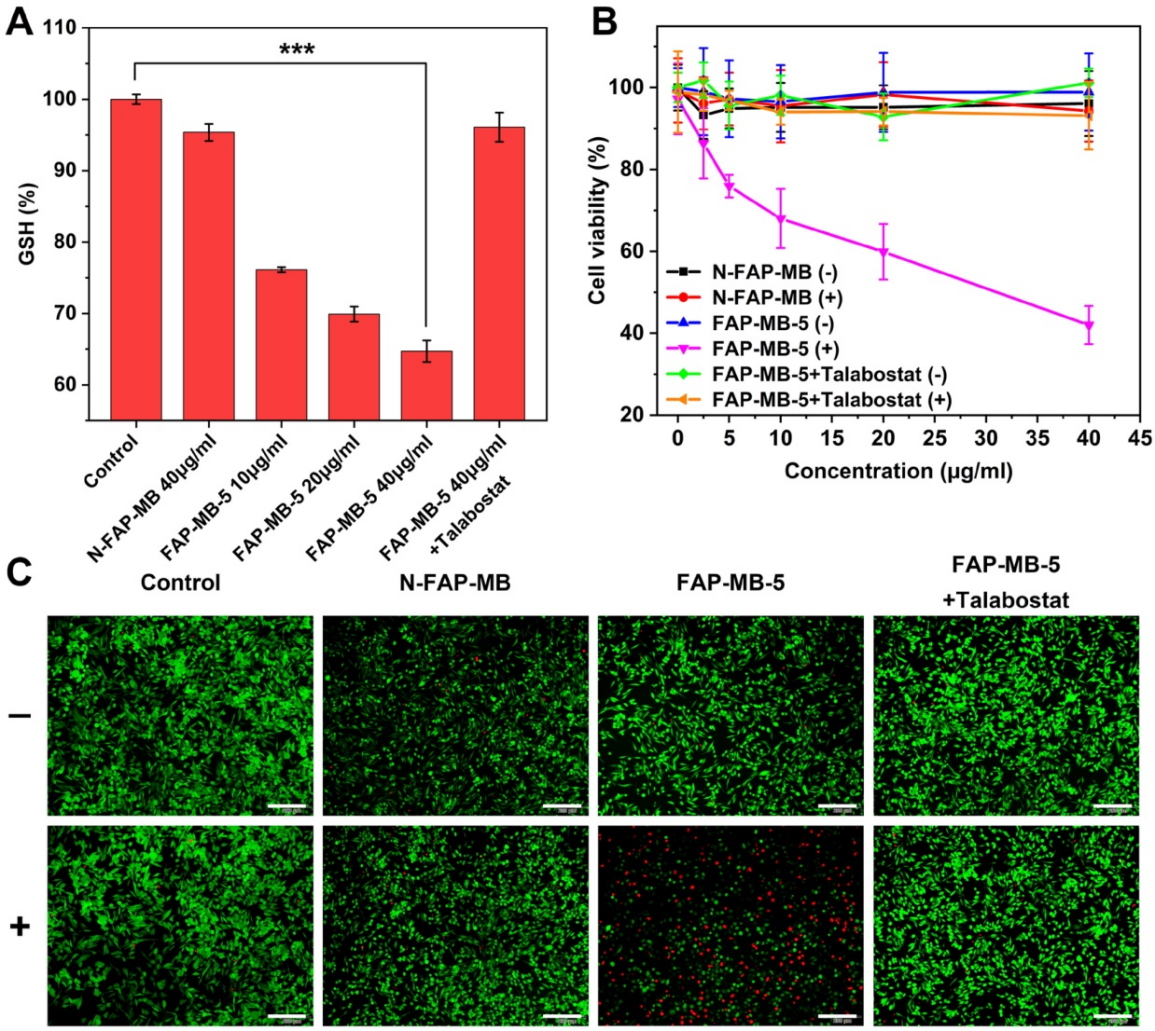
(**A**) Intracellular GSH levels of Mia-paca-2 cells treated with various concentrations of FAP-MB-5 (10, 20, 40 μg/mL) and N-FAP-MB (40 μg/mL). The inhibition group was pre-incubated with talabostat for 1 h and then incubated with FAP-MB-5 (40 μg/mL). (**B**) The cytotoxicity of Mia-paca-2 cells under dark or irradiation condition (633 nm, 100 mW/cm^2^, 5 min) after treatment with FAP-MB-5, N-FAP-MB or FAP-MB-5 pre-incubated with talabostat, respectively. (**C**) Live/dead cell staining assay of Mia-paca-2 cells after incubating with FAP-MB-5 and N-FAP-MB (40 μg/mL) under irradiation (633 nm, 100 mW/cm^2^, 5 min). Live cells and dead cells were stained with FDA (green) and PI (red), respectively. Scale bar: 200 μm. (+) and (-) refer to the treatment with or without irradiation, respectively. Results are described as mean ± SD, *n* = 3. ***p < 0.001.

The highly reactive and electron-deficient Quinone methide (QM) and AQM were widely reported to react with intracellular thiol-relevant nucleophiles such as GSH 56-59. To evaluate the release of AQM and the capture of GSH of FAP-MB-5 in the presence of FAPα, FAP-MB-5 was treated with FAPα and GSH, followed by subjected to LC-MS analysis (**Figure S13**). The product between AQM and GSH (AQM-SG) was detected, indicating the release of AQM and the efficient addition reaction between AQM and GSH. To quantitatively detect intracellular GSH-exhausting effect of AQM, GSH detection kit was utilized as GSH level monitor. As shown in **Figure 4A**, a significant decrease in GSH percent with dose-dependent behavior was observed in FAP-MB-5 group, which suggested that AQM could expend GSH to interrupt intracellular redox homeostasis. By contrast, FAPα inhibition by talabostat caused inefficient AQM production, and exhibited only slight restraint of GSH level. Therefore, above results collectively demonstrated that FAP-MB-5 could achieve enhanced PDT *via* MB-mediated ROS production and AQM-mediated GSH depletion.

### *In vitro* cell cytotoxicity assay

To evaluate the PDT efficacy of FAPα activatable FAP-MB-5 against cancer cells, the cytotoxicity after FAP-MB-5 treatment was examined by MTT assay, apoptosis study and live/dead cell staining. As shown in **Figure 4B** and **Figure S14**, inappreciable change was observed in cells treated with FAP-MB-5 in the dark, demonstrating the good biocompatibility and slight dark toxicity. By contrast, FAP-MB-5 displayed significant concentration-dependent cytotoxicity under irradiation towards Mia-paca-2 cells and the cell viability decreased rapidly to 42%. Although the cell growth inhibition of FAP-MB-5 was not as good as MB because of the delayed and time-consuming hydrolysis of FAP-MB-5 in Mia-paca-2 cells, which is impossible to reach up to 100% hydrolysis in a short incubation time, FAP-MB-5 still displayed much excellent FAPα-responsiveness for effective PDT compared with N-FAP-MB. N-FAP-MB could not affect the cell survival under the conditions of both with and without irradiation and FAP-MB-5 exerted minimal inhibitory effect in the presence of talabostat. These results clearly indicated that PDT efficacy resulted from the FAPα-triggered liberation of photosensitizer MB with succedent irradiation. In addition, NIH-3T3 cells and 4T1 cells were served as FAPα lowly expressed cell lines to verify the FAPα-specific hydrolysis (**Figure S15 and S16**). The cell viability of 4T1 cells and NIH-3T3 cells treated with FAP-MB-5 were both much higher than the FAPα highly expressed Mia-paca-2 cells, indicating the inadequate release of MB in FAPα lowly expressed cells for PDT. These results were forcefully confirmed that FAP-MB-5 could be liberated by the cleavage of FAPα to effectively release MB for specific PDT. Consistent with the cell viability assay, live/dead cell staining further proved that FAP-MB-5 displayed a great potential for efficient PDT (**Figure 4C**). Moreover, the stage of cell apoptosis was distinguished *via* flow cytometry using Annexin V-FITC/PI apoptosis kit. As illustrated in **Figure S17**, FAP-MB-5 with NIR group displayed 51.4% total apoptotic and necrotic cells, which was much higher than other groups. The result was attributed to the specific hydrolysis of FAP-MB-5 by FAPα to liberate photoactive photosensitizer, which eventually leading to the ROS generation and GSH depletion for enhanced intracellular PDT efficacy.

### *In vivo* tumor imaging and bio-distribution

**Figure 5:**
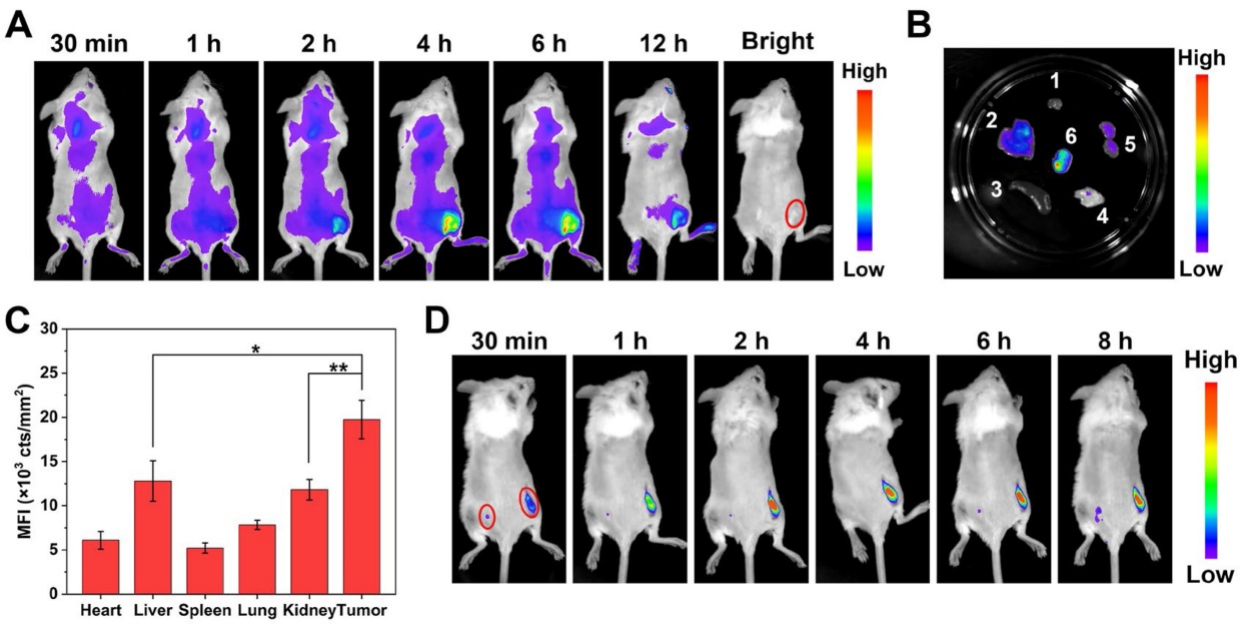
(**A**) *In vivo* fluorescence images of 4T1 tumor-bearing mice after intravenous injection of FAP-MB-5 (7mg/kg). (**B**) *Ex vivo* fluorescence images of tumor and major organs at 12 h post-injection of FAP-MB-5 (7 mg/kg). 1, heart; 2, liver; 3, spleen; 4, lung; 5, kidney; 6, tumor. (**C**) The mean fluorescence intensity (MFI) of major organs and tumor at 12 h poet injection of FAP-MB-5. (**D**) Real-time *in vivo* fluorescence imaging of FAP-MB-5 (7 mg/kg) after injection into the right tumor (right red circle) and the left flank of hypoderm (left red circle) in the tumor-bearing mice, respectively.

We subsequently investigated the performance of FAP-MB-5 as a tumor-specific theranostic pro-photosensitizer for fluorescence imaging in tumor-bearing mice. Although Mia-paca-2 cells were utilized to evaluate the cellular behavior of FAP-MB-5 and PDT effect *in vitro*, it was a great obstacle to establish Mia-paca-2 tumor xenograft model due to its low success rate, so we decided to build 4T1 bearing mice model to conduct the following *in vivo* experiments. FAPα overexpressed on the surface of cancer associated fibroblasts (CAFs) in 4T1 tumor while exhibited extremely low expression in most normal tissues 60, which was verified by western blot and immunofluorescence. (**Figure S18**). Firstly, we investigated the *in vivo* bio-distribution of FAP-MB-5 at 4T1 tumor-bearing mice *via* intravenous injection. The real-time fluorescence images showed that the fluorescence signal gradually enhanced at the tumor region and reached a plateau at 4 h post-injection, indicating that FAP-MB-5 could quickly reach the tumor tissue, followed by specific response to FAPα in tumor site and release free MB to recover strong fluorescence for accurate identification of tumor region (**Figure 5A**). The tumor and major organs were harvested immediately at 12 h post-injection for *ex vivo* fluorescence imaging (**Figure 5B**). The significantly stronger fluorescence signal at tumor site indicated that FAP-MB-5 could efficiently reach and light tumor tissues for subsequent *in vivo* imaging-guided PDT. Furthermore, the quantitative fluorescence statistic of major organs and tumor similarly confirmed the specific tumor-activation of FAP-MB-5 (**Figure 5C**). The biodistribution assay of FAP-MB-5 and MB further verified that FAP-MB-5 displayed tumor-specific hydrolysis followed by MB release, which is favorable basis for fluorescence imaging and subsequently imaging-guided antitumor PDT (**Figure S19**).

In addition, to further confirm the exclusive fluorescence imaging in specific tumor site, FAP-MB-5 was injected into the right tumor tissue with subcutaneously injected at normal tissue as a comparison. As shown in **Figure 5D**, the fluorescence of tumor site was greatly stronger than that of normal tissue. Furthermore, FAP-MB-5 and N-FAP-MB were separately injected into the tumor site of bilateral 4T1 tumor-bearing mice to confirm the specific FAPα activation *in vivo*. As shown in **Figure S20**, the extremely negligible fluorescence signal was observed in left tumor after intratumoral injection with N-FAP-MB at 6 h. By contrast, the right tumor of FAP-MB-5 group showed much stronger fluorescence with time-dependent manner. Above results demonstrated that FAP-MB-5 could serve as a promising FAPα-triggered pro-photosensitizer for precise tumor diagnosis and subsequent *in vivo* imaging-guided PDT.

### *In vivo* antitumor efficacy study and biosafe evaluation

**Figure 6:**
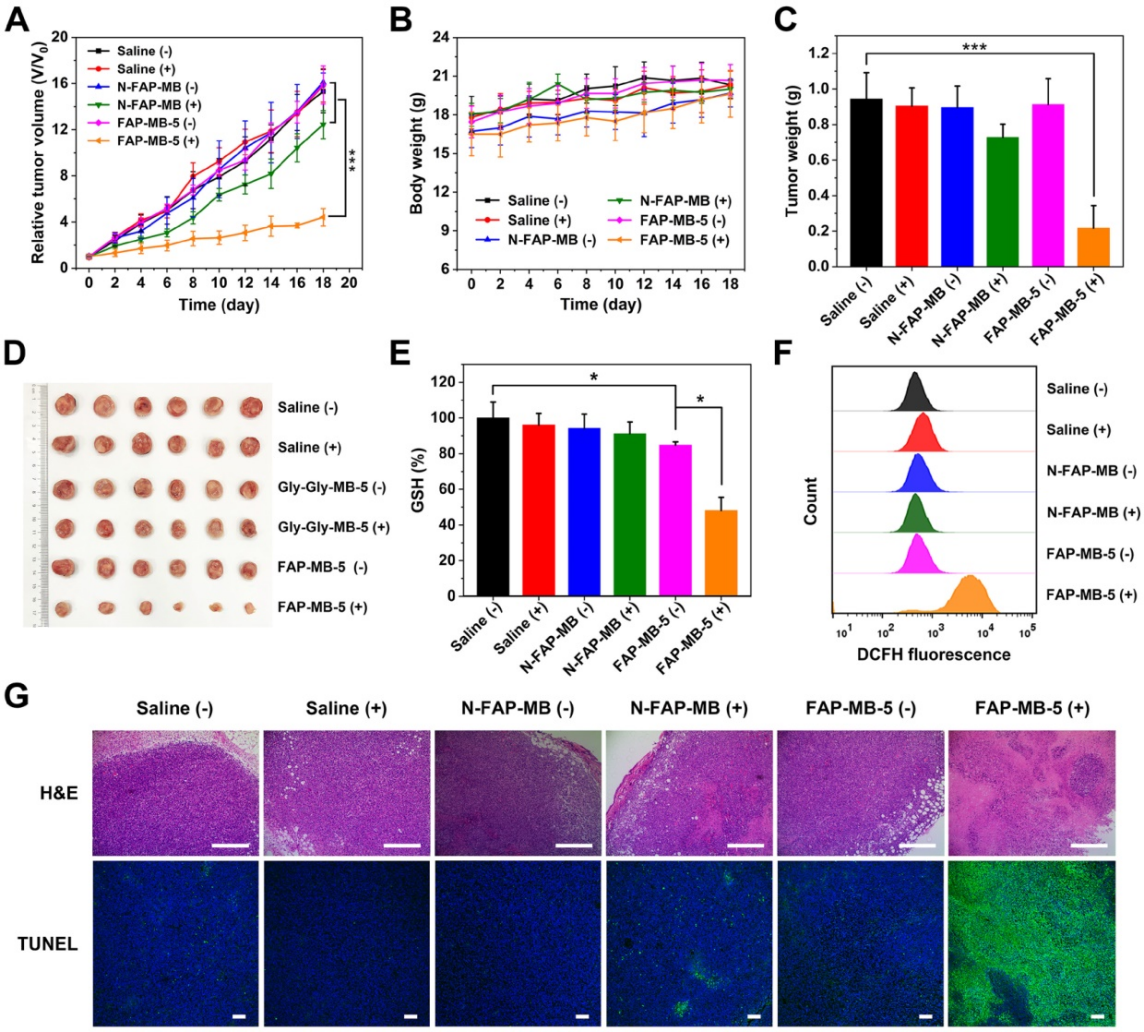
Relative tumor growth curve (**A**) and average body weight (**B**) of 4T1 tumor-bearing mice after various treatments indicated at an equivalent dosage of MB in 18 days. Corresponding average tumor weights (**C**) and representative tumor photographs (**D**) of 4T1 tumor-bearing mice after treatments on 18^th^ day. Intratumoral GSH level (**E**) and ROS generation (**F**) isolated from 4T1 tumor- bearing mice with different treatments. (**G**) The H&E and TUNEL staining of tumor sections after treatments on 18^th^ day. Scale bar: H&E staining 400 μm; TUNEL staining 100 μm. (+) and (-) refer to the treatment with or without NIR irradiation, respectively. Results are described as mean ± SD, *n* = 6. *p < 0.05, **p < 0.01, and ***p < 0.001.

With the confirmation of precise guidance of tumor *in vivo* by fluorescence imaging, we further verified the PDT efficacy of FAP-MB-5 for tumor growth inhibition *in vivo*. The 4T1 tumor bearing mice were randomly divided into 6 groups as follows: saline (without irradiation, -), saline (with irradiation, +), N-FAP-MB (without irradiation, -), N-FAP-MB (with irradiation, +), FAP-MB-5 (without irradiation, -) and FAP-MB-5 (with irradiation, +). At 4 h post-intravenous injection, laser groups were exposed to irradiation for 5 min. As shown in **Figure 6A**, similar to saline (-) group, saline (+) group showed negligible inhibition on tumor growth, indicating that only irradiation for 5 min had no influence on rapid tumor growth. In addition, unapparent antitumor effect was observed in N-FAP-MB groups with irradiation or not, further demonstrating that N-FAP-MB failed to release photosensitizer MB for available PDT in tumor region because of the lack of FAPα-sensitive dipeptide. By contrast, FAP-MB-5 (+) group exhibited remarkable tumor inhibition, which certified the effective FAPα-triggered MB release as well as rapid GSH depletion followed by abundant ROS generation and oxidation stress amplification under irradiation for enhanced PDT. The weigh date and photograph of the excised tumors with different treatment were consistent with tumor growth curves shown in **Figure 6C** and** 6D**. The tumors isolated from mice were collected to measure intratumoral GSH level and ROS generation (**Figure 6E** and **6F**). It is noted that the treatment of FAP-MB-5 under irradiation apparently reduced intracellular GSH level and extensive ROS production, indicating *in situ* FAPα-activation of FAP-MB-5 in the tumor site, and irradiation could induce intense oxidative stress accompanied by GSH consumption and ROS outburst to achieve enhanced PDT efficacy. Furthermore, the representative tumor tissue slices of various treatment groups were characterized by H&E and TUNEL staining, and the results showed that FAP-MB-5 (+) displayed the most valid cell apoptosis and necrosis compared with other groups (**Figure 6G**).

**Figure 7:**
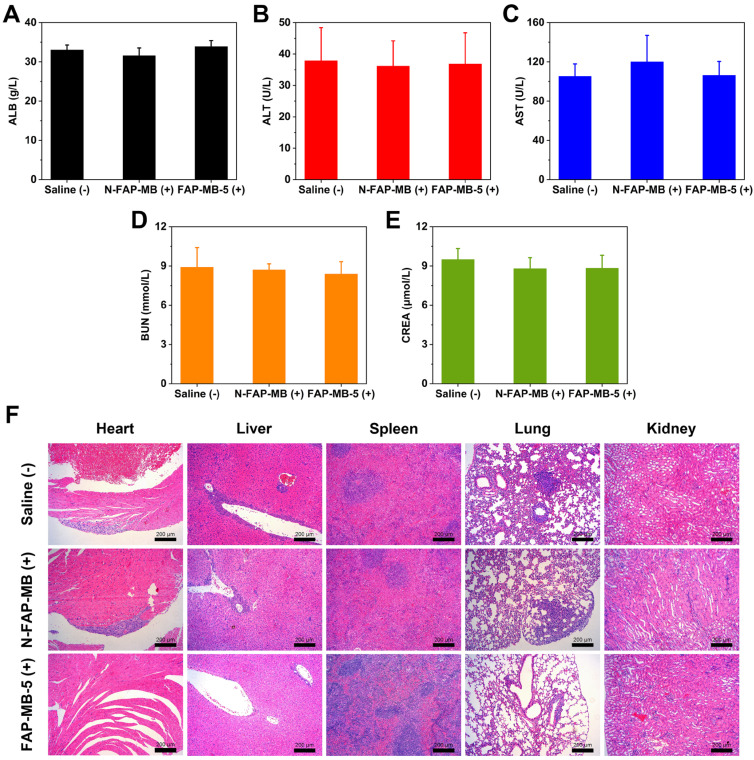
Serum biochemistry analysis ALB (**A**), ALT (**B**), AST (**C**), BUN (**D**), CREA (**E**) of different groups after the 18-day treatment. (**F**) The H&E staining of major organs slices in various groups on 18^th^ day. Scale bar: 200 μm. (+) and (-) refer to the treatment with or without NIR irradiation, respectively. Results are described as mean ± SD, *n* = 6.

To assess the side effects of various treatments *in vivo*, the body weight of mice was monitored during treatment period. The major organs were collected for H&E staining and the blood samples were used to perform serum biochemistry analysis. All groups exhibited no obvious body weight loss and noticeable abnormality in major organs, implying negligible systemic toxicity of all treatments (**Figure 6B and 7F**). Meanwhile, serum biochemistry analysis of FAP-MB-5 (+) including albumin (ALB), alanine aminotransferase (ALT), aspartate aminotransferase (AST), urea nitrogen (BUN) and creatinine (CREA), displayed no significant distinction compared with saline (+) group, which confirmed the superior biocompatibility (**Figure 7A-7E**). Collectively, FAP-MB-5 was a potential tumor activatable “one for all” pro-photosensitizer for superior tumor theranostics with distinguished biocompatibility.

## Conclusion

In summary, we presented the design and synthesis of novel FAPα activatable pro-photosensitizer FAP-MB-X with different *N*-terminal blocked structures for tumor-selective NIR fluorescence imaging and high-efficiency PDT. It was clearly verified that candidate compound 5 (FAP-MB-5) was the most suitable substrate towards FAPα compared with other compounds. FAP-MB-5 displayed favorable blood stability, excellent FAPα-responsiveness and satisfactory tumor-specific imaging capacity. Under tumor microenvironment, FAP-MB-5, whose fluorescence and photoactivity were initially quenched, was activated by recognition towards FAPα with enzymatic reaction, followed by 1,6-elimination to release photosensitizer MB and GSH consumption agent AQM. This tumor-specific activation could result in inherent photoactivity recovery for precise tumor diagnosis and imaging-guided/enhanced PDT. Moreover, FAP-MB-5 exhibited distinguished biocompatibility during treatment. Thereby, FAP-MB-5 could serve as a promising FAPα- activatable theranostic photosensitizer owing to its accurate tumor imaging, remarkable tumor-specific PDT effect and favorable biosafety. This study provides a new approach for developing tumor-specific enzyme activatable photosensitizer for precise cancer diagnosis and high-specific PDT with improved biosafety.

## Methods

### Materials, cells, animals

All reagents and solvents were purchased from Bide Pharmatech Ltd. (Shanghai, China) and Aladdin Reagent Inc. (Shanghai, China). rhFAPα and rhDPPIV were purchased from Biolegend (San Diego, USA). Legumain and GSH Detection Kit were purchased from Solarbio (Beijing, China). Aminopeptidase N/CD13 was purchased from R&D (Minnesota, USA). Talabostat mesylate was purchased from TargetMol (Shanghai, China). Methylene blue (MB), 2′,7′-dichlorofluorescein diacetate (DCFH-DA), 3-(4.5-dimethyl-thiazol-2-yl)-2.5-diphenyl tetrazolium bromide (MTT), 1,3-diphenylisobenzofuran (DPBF), esterase, collagenase, penicillin-streptomycin were acquired from Sigma-Aldrich (St. Louis, USA). Dulbecco's Modified Eagle's Medium (DMEM), pancreatic enzymes and fetal bovine serum (FBS) were provided by Gibco (California, USA). Annexin V/PI Apoptosis Detection Kit was acquired from eBiosciences (Hatfield, UK). FAPα antibody was purchased from Abcam (Cambridge, UK), and GADPH antibody was purchased from Proteintech Group (Pennsylvania, USA).

Murine breast cancer cells 4T1 were cultured in DMEM supplemented with 10% FBS and 1% penicillin-streptomycin. Mia-paca-2, the human pancreatic cancer cell lines, were cultured in DMEM supplemented with 10% FBS, 2.5% Horse Serum and 1% penicillin-streptomycin. All of the cells were obtained from Laboratory Animal Center of Sun Yat-sen University (Guangzhou, China) and both were maintained at 37 °C in a 5% CO_2_ humidified incubator.

BALB/c female mice (4-6 weeks old) were ordered from the Laboratory Animal Center of Sun Yat-sen University (Guangzhou, China). All animal experimental procedures were performed in accordance with the National Institute of Health Guidelines under the protocols, approved and conducted with the Institutional Animal Care and Use Committee of Sun Yat-sen University.

### Synthesis of FAP-MB-X

#### Synthesis of compound A

To a solution of methylene blue (10 g, 31.26 mmol) and Na_2_CO_3_ (13.25 g, 125.01 mmol) in water (100 mL) were added DCM (50 mL), the reaction mixture was stirred at 40 °C. Na_2_S_2_O_4_ (21.77 g, 125.04 mmol) was dissolved in 100 mL of water and added dropwise. The mixture was stirred until the solution became yellow and then cooled with an ice bath, triphosgene (5.57 g, 18.77 mmol) in 50 mL of DCM was dropped into the solution. After the reaction was completed, the solution was poured into ice-water while stirring, then filtered through diatomite, extracted with DCM and washed with brine. The organic layer was dried over anhydrous Na_2_SO_4_, filtered and concentrated. The crude product was purified by silica gel chromatography using PE/EA (5: 1) as eluent to yield compound A as white solid (7.25 g, 66.7%). LC-MS (ESI, *m/z*): calcd. for C_17_H_19_ClN_3_OS [M+H]^+^ 348.09, found 348.15; ^1^H NMR (400 MHz, DMSO-*d*_6_) δ 7.42 (d, *J* = 8.9 Hz, 2H), 6.79 (d, *J* = 2.8 Hz, 2H), 6.71 (dd, *J* = 8.9, 2.8 Hz, 2H), 2.93 (s, 12H).

#### Synthesis of compound B

(*S*)-1-(2-(tert-butoxycarbonylamino)acetyl)pyrrolidine-2-carboxylic acid (Boc-Gly-Pro-OH, 0.5 g, 1.84 mmol), 2-(7-Azabenzotriazol-1-yl)-*N,N,N',N'*-tetramethyluronium hexafluorophosphate (HATU, 0.838 g, 2.2 mmol), *N,N*-Diisopropylethylamine (DIPEA, 0.475 g, 3.68 mmol) were added to 50 mL of DMF. *p*-aminobenzyl alcohol (0.249 g, 2.2 mmol) was slowly added under an ice bath and then stirred at room temperature. After the reaction was completed, the solvent was evaporated under reduced pressure. The residue was dissolved in DCM, then successively washed with water and brine. The organic phase was dried over anhydrous Na_2_SO_4_, filtered and concentrated. The residue was purified by silica gel chromatography using DCM/MeOH (30: 1) as eluent to yield compound B as faint yellow solid (0.515 g, 74.3%). LC-MS (ESI, *m/z*): calcd. for C_19_H_26_N_3_O_5_ [M-H]^-^ 376.20, found 376.20; ^1^H NMR (400 MHz, DMSO-*d*_6_) δ 9.88 (s, 1H), 7.53 (d, *J* = 8.5 Hz, 2H), 7.23 (d, *J* = 8.4 Hz, 2H), 6.80 (t,* J* = 5.9 Hz, 1H), 5.18-4.98 (m, 1H), 4.43 (d, *J* = 5.0 Hz, 2H), 3.91-3.64 (m, 2H), 3.64-3.44 (m, 2H), 2.20-1.76 (m, 4H), 1.38 (s, 9H).

#### Synthesis of compound C

Compound B (0.8 g, 0.33 mmol), 4-Dimethylaminopyridine (DMAP, 0.285 g, 0.37 mmol), Na_2_CO_3_ (0.674 g, 1 mmol) were added to 20 mL of DCM. Compound A (0.811 g, 0.37 mmol) in 10 mL of DCM was dropped into the above mixture in an ice-water bath and then stirred at room temperature until the reaction was completed as monitored by TLC analysis. The reaction mixture was filtered and followed by being washed with water, 0.2 M HCl and brine. The organic layer was dried over anhydrous Na_2_SO_4_, filtered and concentrated. The crude product was purified by silica gel chromatography using DCM/MeOH (25: 1) as eluent to yield compound C as white solid (0.728 g, 49.8%). LC-MS (ESI, *m/z*): calcd. for C_36_H_45_N_6_O_6_S [M+H]^+^ 689.30, found 689.45; HPLC purity: 96.85%; ^1^H NMR (400 MHz, DMSO-*d*_6_) δ 10.04 (s, 1H), 7.59 (d, *J* = 8.3 Hz, 2H), 7.42-7.22 (m, 4H), 6.78 (t, *J* = 5.8 Hz, 1H), 6.70-6.60 (m, 4H), 5.10 (s, 2H), 4.55-4.34 (m, 1H), 3.89-3.63 (m, 2H), 3.62-3.39 (m, 2H), 2.88 (s, 12H), 2.21-1.79 (m, 4H), 1.38 (s, 9H); ^13^C NMR (101 MHz, DMSO-*d*_6_) δ 171.03, 168.04, 156.26, 154.06, 149.08, 139.31, 132.45, 131.39, 128.81, 128.05, 127.47, 119.54, 111.35, 110.19, 78.37, 67.37, 60.77, 46.34, 42.89, 40.65, 29.74, 28.67, 24.90.

#### Synthesis of compound D

Trifluoroacetic acid (TFA, 5 v/m) was added dropwise to the solution of compound C (0.4 g) in anhydrous DCM (45 v/m) under an ice bath and stirred for 30 min, followed by being stirred at room temperature. After the reaction was completed, the pH of reaction solution was adjusted to 12 with a.q. 5% NaOH solution, and the organic layer was separated, washed with brine. The organic phase was dried over anhydrous Na_2_SO_4_, filtered and concentrated to afford a solid residue, which was subsequently purified by silica gel chromatography using DCM/MeOH (20: 1) as eluent to yield compound D as white solid (0.26 g, 76.1%). LC-MS (ESI, *m/z*): calcd. for C_31_H_37_N_6_O_4_S [M+H]^+^ 589.25, found 589.35; HPLC purity: 99.71%; ^1^H NMR (400 MHz, DMSO-*d*_6_) δ 10.05 (s, 1H), 7.57 (d,* J* = 8.4 Hz, 2H), 7.39-7.17 (m, 4H), 6.73-6.57 (m, 4H), 5.09 (s, 2H), 4.57-4.39 (m, 1H), 3.60-3.46 (m, 4H), 2.88 (s, 12H), 2.14-1.80 (m, 4H); ^13^C NMR (126 MHz, DMSO-*d*6) δ 171.17, 154.06, 149.06, 139.34, 132.43, 131.38, 128.86, 128.01, 127.48, 119.84, 119.53, 111.33, 110.18, 67.38, 60.67, 46.11, 43.74, 40.64, 29.84, 24.79.

### Synthesis of FAP-MB-1~10

Method A: Compound D (0.17 mmol), acid anhydride (0.34 mmol), DMAP (0.034 mmol) were added to 25 mL of anhydrous DCM. After stirring for 4 h at room temperature, the reaction mixture was washed with 0.2 M HCl and brine. The organic phase was dried over anhydrous Na_2_SO_4_, filtered and concentrated to afford a solid residue, which was subsequently purified by silica gel chromatography using DCM/MeOH (25: 1) as eluent to yield target compound.

Method B: Compound D (0.17 mmol), acid (0.2 mmol), HATU (0.21 mmol), DIPEA (0.35 mmol) were added to 25 mL of anhydrous DCM. The reaction mixture was stirred at room temperature for 5 h. After the reaction completion, the mixture was washed successively with water and brine. The organic layer was dried over anhydrous Na_2_SO_4_, filtered and concentrated. The crude product was purified by silica gel chromatography using DCM/MeOH (25: 1) as eluent to yield target compound.

Method C: Alcohol (1.83 mmol), DIPEA (2.2 mmol) were added to 25 mL of anhydrous DCM. *p*-nitrophenyl chloroformate (2.2 mmol) was slowly added in batches under an ice bath. The reaction mixture was stirred at room temperature until the reaction was completed as monitored by TLC analysis. Then the mixture was washed with 0.2 M HCl and brine, the organic phase was dried over anhydrous Na_2_SO_4_, filtered and removed under reduced pressure, which was used in the next step without further purification. Such crude active carbonate intermediate (0.21 mmol), Compound D (0.17 mmol), DIPEA (0.51 mmol) were added to 25 mL of anhydrous DCM. The reaction mixture was stirred at room temperature for 4 h. After the reaction was completed, the mixture was washed with 0.2 M HCl and brine. The organic layer was dried over anhydrous Na_2_SO_4_, filtered and evaporated on a rotary evaporator to afford a solid residue, which was subsequently purified by silica gel chromatography using DCM/MeOH (25: 1) as eluent to yield target compound.

Method D: Compound D (0.17 mmol), Fmoc-AA-OH (0.2 mmol), HATU (0.21 mmol), DIPEA (0.35 mmol) were added to 25 mL of anhydrous DCM. The reaction mixture was stirred at room temperature for 5 h. After the reaction completion, the solvent was removed under reduced pressure, and 50% 1, 8-Diazabicyclo[5.4.0]undec-7-ene (DBU)-DCM solution was added, the resulting mixture was stirred at room temperature for 2 h. Upon reaction accomplished, the mixture was washed successively with water and brine. The organic layer was dried over anhydrous Na_2_SO_4_, filtered and concentrated. The crude product was purified by silica gel chromatography using DCM/MeOH (25: 1) as eluent to yield target compound.

### *In vitro* rhFAPα-mediated hydrolysis of pro-photosensitizers

The reaction buffer containing different pro-photosensitizers (300 μM) with rhFAPα (0.45 μg/mL) was maintained at 37 °C for 4 h, respectively. Methanol was used to terminate the reaction and the mixtures were centrifuged at 12000 rpm for 15 min. The supernatant was subjected to HPLC (HITACHI, Japan) analysis using an isocratic mobile phase, which was a mixture of acetonitrile and water supplementing with 0.1% trifluoroacetic acid (40: 60, v/v), and the wavelength was set at 665 nm. The standard curve was plotted using the peak area against various concentrations of MB (1.5-200 μM).

### Kinetic assay

Various concentrations of FAP-MB-1 (12.5, 25, 50, 100, 200, 400, 800 μM) or FAP-MB-5 (6.25, 12.5, 25, 50, 100, 200, 400 μM) were incubated with rhFAPα (0.45 μg/mL) at 37 °C for 15 min under assay buffer (50 mM Tris, 1 M NaCl, 0.1% BSA, pH = 7.5), whereas FAP-MB-8 (1.563, 3.125, 6.25, 12.5, 25, 50, 100 μM) was incubated with rhFAPα at 37 °C for 30 min, respectively. After incubation, methanol was used to terminate the reaction and the mixtures were centrifuged at 12000 rpm for 15 min. The supernatant was injected into HPLC for quantification analyses. Then initial velocity (nmol/min) was calculated, and plotted against different concentrations of FAP-MB-1, FAP-MB-5 or FAP-MB-8 following the Michaelis-Menten curve. The kinetic parameters were calculated using the Michaelis-Menten equation shown below:

*V* = *V*_max_ × [S] / (*K*_m_ + [S]), where *V* is the initial velocity, and [S] is a series of substrate concentration.

### *In vitro* blood stability of pro-photosensitizers

FAP-MB-1, FAP-MB-5 and FAP-MB-8 (2.5 μM) were incubated with the blood samples which were collected from orbit of mice at 37 °C for 4 h, respectively. Meanwhile, the fluorescence intensity of FAP-MB-1, FAP-MB-5 and FAP-MB-8 (2.5 μM) were measured in PBS solution, respectively. The fluorescence emission spectra of supernatant were measured by a fluorescence spectrophotometer (excitation: 630 nm; emission: 650-720 nm) (Fluoromax-4, Horiba, USA).

### *In vitro* rhFAPα activation and selectivity study

For activation study, FAP-MB-5 (200 μM) was incubated with rhFAPα at 37 °C in assay buffer (50 mM Tris, 1 M NaCl, 0.1% BSA, pH = 7.5). As inhibition group, rhFAPα (0.45 μg/mL) was pre-incubated with talabostat (100 μM) for 1 h followed by incubation with FAP-MB-5 (200 μM). As negative control group, N-FAP-MB (200 μM) was incubated with rhFAPα (0.45 μg/mL). The concentrations of rhFAPα incubated with FAP-MB-5 and the reaction time were showed in corresponding figure caption. The fluorescence emission spectra of the supernatant were measured by fluorescence spectrophotometer (excitation: 630 nm; emission: 650-720 nm).

FAP-MB-5 (500 μM) was mixed with various concentrations of rhFAPα in assay buffer for 1 h. As inhibition group, rhFAPα (0.45 μg/mL) was pre-incubated with talabostat (100 μM) for 1 h. UV-vis absorbance spectra were recorded using UV-vis spectrophotometer (UV2600, Techcomp, China) in the range from 450 to 800 nm.

FAP-MB-5 (400 μM) was incubated with rhFAPα (0.45 μg/mL) at 37 °C for 1 h, 2 h, 4 h, respectively. As a comparison, rhFAPα was pre-incubated with talabostat (100 μM) for 1 h. Methanol was used to terminate the reaction and the mixture was subjected to HPLC analysis using the mixture of acetonitrile and H_2_O supplementing with 0.1% trifluoroacetic acid (40: 60, v/v) as mobile phase at 260 and 665 nm. N-FAP-MB (400 μM) was incubated with rhFAPα (0.45 μg/mL) at 37 °C for 1 h and 4 h, respectively. The supernatant was subjected to HPLC analysis using the mixture of methanol and H_2_O supplementing with 0.1% trifluoroacetic acid (40: 60, v/v) as mobile phase at 260 and 665 nm.

For selectivity study, FAP-MB-5 (200 μM) were treated with rhFAPα (0.225 μg/mL) in Tris buffer (50 mM Tris, 1 M NaCl, 0.1% BSA, pH = 7.5), DPPIV in Tris buffer (25 mM Tris, pH = 8.0), APN in Tris buffer (50 mM, pH = 7.0), legumain in MES buffer (50 mM MES, 250 mM NaCl, pH = 5.0), and BSA, esterase, collagenase, NaCl, MgCl_2_, FeCl_3_, GSH, V_c_, H_2_O_2_, Gly, Pro, Cys in PBS (pH = 7.4) at 37 °C for 1 h, respectively. The concentration of protein, as described above containing BSA, DPPIV, APN, legumain, esterase, collagenase was 0.45 μg/mL and the rest of potential interfering substances concentrations were 50 μM. The fluorescence intensity of the solutions was measured by fluorescence spectrofluorometer.

### *In vitro* tissue-mediated hydrolysis

Tumor, heart, liver, spleen, lung, kidney tissues harvested from 4T1 tumor-bearing mice were weighed and homogenized under ice in Tris buffer (50 mM Tris, 100 mM NaCl, pH = 7.4) by using tissue homogenizer, while the blood sample was collected from orbit of mice. FAP-MB-5 (2.5 μM) was incubated with various homogenates of tissues at 37 °C for 4 h. The fluorescence emission spectra of supernatant were measured by a fluorescence spectrophotometer (excitation: 630 nm; emission: 650-720 nm).

### *In vitro* singlet oxygen generation

DPBF was used to confirm the singlet oxygen generation *in vitro*. Briefly, the reaction buffer containing FAP-MB-5 (200 μM) and rhFAPα (0.45 μg/mL) was maintained at 37 °C for 4 h. In addition, rhFAPα was pre-incubated with talabostat (100 μM) for 1 h for inhibition control. As negative group, N-FAP-MB (200 μM) was incubated with rhFAPα (0.45 μg/mL) for 4 h. Methanol was used to terminate the reaction and the mixtures were centrifuged at 12000 rpm for 15 min. The supernatant was mixed with DPBF (50 μM, methanol) into 2 mL followed by irradiation with NIR laser (633 nm, 100 mW/cm^2^) for the scheduled time. The absorbance variation at 416 nm was monitored by UV-vis spectroscopy. Meanwhile, electron spin resonance (ESR) spectroscopy was performed to monitor ^1^O_2_ production using 2,2,6,6-tetramethylpiperidine (TEMP) as ^1^O_2_ trapper. After incubating with rhFAPα (0.45 μg/mL) for 4 h, methanol was added into the samples and mixed with TEMP followed by irradiation with NIR laser (633 nm, 100 mW/cm^2^) for 30s. Afterward, the ESR spectra was recorded by electron paramagnetic resonance spectrometer (EMXplus, Bruker, Germany).

### Docking Analysis

The 2D structure of FAP-MB-5 was generated by ChemDraw, then converted to 3D structure using Maestro LigPrep in OPLS3 force field, version 2.1.207. The X-ray structure of FAPα (PDB code 1Z68) was obtained from the Protein Data Bank (http://www.rcsb.org/pdb). The Grid files were obtained following the standard procedure recommended by Schrödinger. The 3D structure of ligand was docked flexibly using Glide in XP mode, and other docking parameters were set to default values. Ten predicted poses were obtained during the docking process, and the Grid-based score was calculated for each pose. The image files were generated by PyMOL (version 1.7).

### Cell imaging and cytotoxicity assay

Mia-paca-2 cells were seeded into 12-well plates for 24 h. Then the cells pre-incubated with or without talabostat (50 μM) for 1 h were incubated with FAP-MB-5 or N-FAP-MB (both 40 μg/mL) for 1 h, 2 h and 4 h, respectively. Afterward, the cells were washed with PBS, stained with DAPI, and then imaged by confocal laser scanning microscopy (FV3000, Olympus, Japan).

The cytotoxicity of FAP-MB-5 and N-FAP-MB against Mia-paca-2 cells were verified by the MTT assay. Concretely, Mia-paca-2 cells were seeded into 96-well plates and treated with free MB, FAP-MB-5 and N-FAP-MB at equal dosage of MB. As a comparison, the cells were pre-treated with talabostat (50 μM) for 1 h before adding FAP-MB-5. After incubation for another 12 h, cells were irradiated with or without NIR laser (633 nm, 100 mW/cm^2^) for 5 min. The cell viability was detected by MTT assay after incubation for 12 h. In addition, the cell viability of FAP-MB-5 against 4T1 cells and NIH-3T3 cells with or without irradiation (633 nm, 100 mW/cm^2^) were also measured by MTT assay.

### Intracellular ROS generation

Mia-paca-2 cells were incubated with FAP-MB-5 or N-FAP-MB (both 40 μg/mL) for 12 h. The cells were washed three times, and incubated with ROS probe DCFH-DA (10 μM) for 30 min. The cells were irradiated with or without NIR laser (633 nm, 100 mW/cm^2^) for 5 min. As an inhibition group, the cell was pre-incubated with talabostat (50 μM) for 1 h before adding FAP-MB-5. The intracellular ROS was measured *via* flow cytometry (Guava EasyCyte 6-2L, Merck Millipore) and CLSM, respectively.

### Intracellular GSH level

Mia-paca-2 cells were cultured with different concentrations of FAP-MB-5 (10, 20, 40 μg/mL) or N-FAP-MB (40 μg/mL). After incubation for 12 h, the cells were harvested, washed, and lysed *via* freeze-thaw cycles three times. The lysates were centrifuged and the supernatant was collected to detect GSH level using GSH Detection Kit *via* microplate reader at 412 nm.

### Cell apoptosis and live/dead cell staining assay

Mia-paca-2 cells pre-incubated with talabostat (50 μM) for 1 h or not were treated with FAP-MB-5 and N-FAP-MB at the concentration of both 40 μg/mL for 12 h, and then irradiated with or without NIR laser (633 nm, 100 mW/cm^2^) for 5 min, respectively. After incubation for further 12 h, the cellular apoptosis degree was evaluated by Annexin V-FITC/PI apoptosis kit according to manufacturer's instructions.

For live/dead cell staining assay, Mia-paca-2 cells were stained with the mixtures of FDA (5 μg/mL) and PI (5 μg/mL) for 15min after different treatments, and subsequently imaged by Inverted Fluorescent Microscope (IX73, Olympus, Japan).

### Western blot

Tissues were lysed in cold RIPA buffer with protease inhibitors for 30 min, and the total protein concentration was determined by BCA assay kit. Protein extracts (20 μg) were separated by 10% SDS-PAGE gels and transferred to PVDF membranes. Afterward, the membranes were blocked with 5% skim milk for 1 h and performed with indicated primary antibodies and anti-rabbit IgG, HRP-linked antibody in sequence. Blots were imaged on chemiluminescence reagents autoradiography (BIO-RAD, ChemiDoc XRS+, USA). GADPH was served as an internal reference, and quantification of the protein band intensities were conducted by ImageJ software.

### *In vivo* fluorescence imaging for diagnosis and biodistribution analysis

4T1 tumor-bearing mice model was established by subcutaneously injection of 4T1 cells into the flanks of Balb/c mice. When tumor volumes approached 100 mm^3^, the mice were randomly divided into 2 groups (*n* = 3). For evaluating the FAP-mediated MB imaging ability, FAP-MB-5 at an equal MB dose of 3 mg/kg was injected intravenously into 4T1 tumor-bearing mice. Fluorescent images were taken at 0.5, 1, 2, 4, 6, 12 h post-injection by small animal imaging system (excitation: 630 nm, emission: 680 nm) (Night OWL LB983, Berthold, Germany). After 12 h post-injection, the major organs and tumor were harvested for further imaging. Furthermore, FAP-MB-5 (7 mg/kg) was injected directly into the right tumor and the left flank of hypoderm, respectively, and then monitored at time points of 0.5, 1, 2, 4, 6, 8 h with the same illumination settings as described above.

Balb/c female mice were inoculated subcutaneously with 1 × 10^6^ 4T1 cells on both left and right flanks to create bilateral tumor xenograft model. After the tumor volumes reaching about 100 mm^3^, bilateral 4T1 tumor-bearing mice were injected with FAP-MB-5 into tumor on right side, and injected with N-FAP-MB into tumor on the other side simultaneously. The fluorescent images were performed after 2, 4, 6 h of injection.

For biodistribution assay, the mice intravenously injected with FAP-MB-5 were sacrificed at 4 h post-injection, and tumor tissues and main organs were harvested, weighted and homogenized to detect the content of original FAP-MB-5 and the released MB in different tissues via HPLC and fluorescence spectrofluorometer, respectively.

### *In vivo* PDT antitumor efficacy and biosafety

Balb/c mice inoculating 4T1 tumors were randomly divided into 6 groups (*n* = 6) including saline (without irradiation), saline (with irradiation), N-FAP-MB (without irradiation), N-FAP-MB (with irradiation), FAP-MB-5 (without irradiation), FAP-MB-5 (with irradiation). The mice were intravenously injected with different administrations at a MB dose of 3 mg/kg every two days for four times. The mice were exposed to 633 nm laser (1 W/cm^2^) for 5 min after 4 h post vein injection. The tumor volumes and body weights were monitored every other day for 20 days after the first injection. The tumor volumes were calculated with the following formula: V = (tumor length) × (tumor width)^2^ / 2.

At the end of the treatments, the major organs including heart, liver, spleen, lung, kidney and tumor were collected for H&E and TUNEL staining analysis. Moreover, the blood samples were obtained from orbit of treated mice for serum biochemistry analysis including alanine aminotransferase (ALT), albumin (ALB), aspartate aminotransferase (AST), urea nitrogen (BUN) and creatinine (CREA).

### Evaluation of intratumoral ROS generation and GSH depletion

4T1 tumor-bearing mice were randomly divided into 6 groups (*n* = 6) as above description, and intravenously injected with various formulations same as antitumor experiments every two days for four times. For the examination of tumoral GSH, the tumors were harvested, weighed and lysed to measure intratumoral GSH level to the instruction of kit. In additional, the intratumoral ROS generation was detected by flow cytometry. Briefly, DCFH (100 μM) was injected intratumorally before 30 min of irradiation of last treatment. The tumors were separated into single cells through 200 μm and 70 μm filter for FCM analysis.

### Statistical analysis

Data were presented as mean ± standard deviation (S.D.). Significant differences were calculated using one-way analysis of variance. Statistical significance was performed as *p < 0.05, **p < 0.01 and ***p < 0.001.

## Supplementary Material

Supplementary methods and figures.
