# Supplementary material for: Fibroblast activation protein α activatable theranostic pro-photosensitizer for accurate tumor imaging and highly-specific photodynamic therapy
Source: Theranostics. 2022 May 1;12(8):3610–27. doi: 10.7150/thno.70308 (PMC9131278; doi:10.7150/thno.70308)
Supplement: Supplementary file 1 — Supplementary methods and figures. [file thnov12p3610s1.pdf]

Supplementary Material

Fibroblast activation protein α activatable theranostic pro-photosensitizer for accurate tumor imaging and highly-specific photodynamic therapy

Yong Luo[1,#], Zishan Zeng[1,#], Ting Shan[1], Xiaoyu Xu[1], Jie Chen[1], Yuanfeng He[1], Tao Zhang[1], Zeqian Huang[1], Guihong Chai[1], Yanjuan Huang[1], Yanfang Zhao[2] and Chunshun Zhao[1,*]

1. School of Pharmaceutical Sciences, Sun Yat-sen University, Guangzhou, 510006, P. R. China.
2. Key Laboratory of Structure-Based Drug Design and Discovery, Ministry of Education, Shenyang Pharmaceutical University, 103 Wenhua Road, Shenhe District, Shenyang, 110016, P. R. China.

*Corresponding author: Chunshun Zhao; E-mail: zhaocs@mail.sysu.edu.cn

[#]These authors contribute equally to this work.

Synthesis and characterization of FAP-MB-1~10

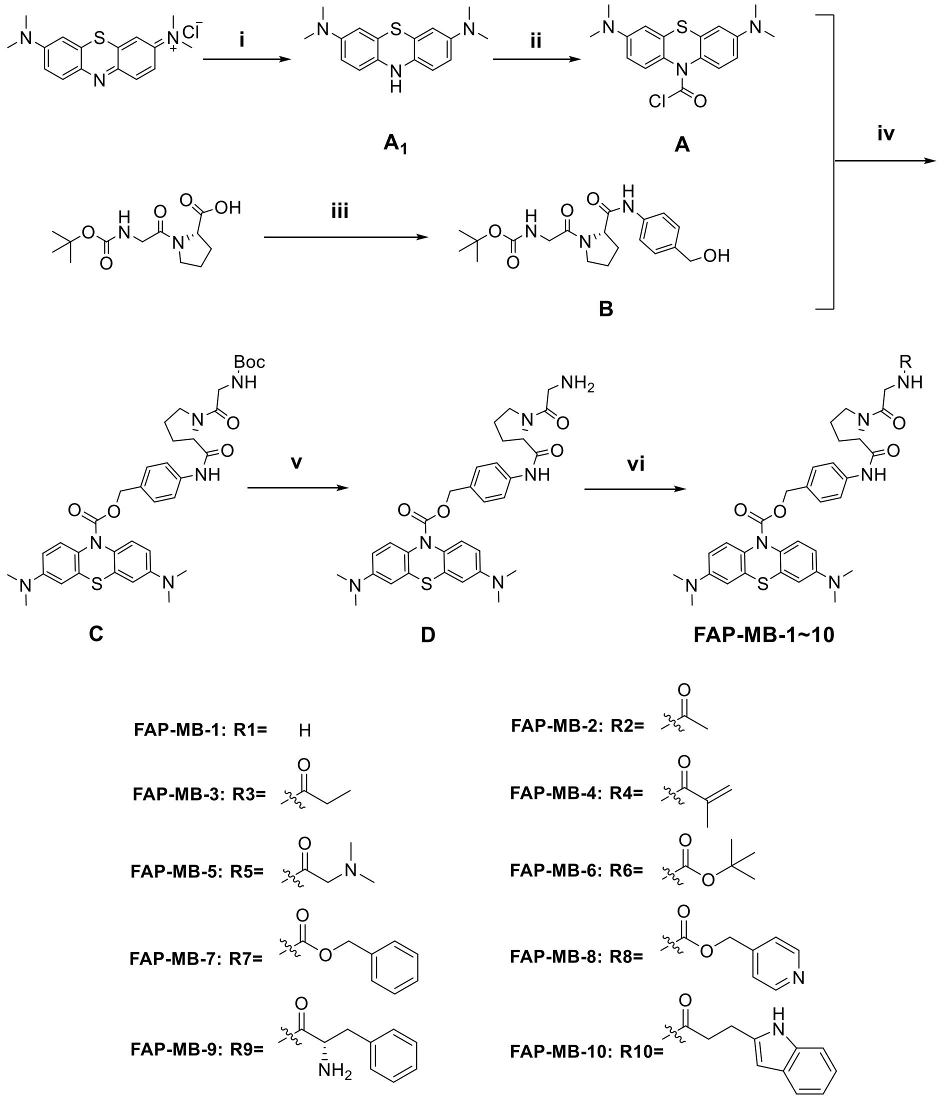

Scheme S1. Synthesis route of FAP-MB-1~10. Reagents and conditions: (i) Na_2CO_3, $Na_2S_2O_4$, DCM/H_2O, 40 °C; (ii) Triphosgene, Na_2CO_3, DCM, 0 °C; (iii) *p*-Aminobenzyl alcohol, HATU, DIPEA, DMF, r.t.; (iv) Na_2CO_3, DMAP, DCM, r.t.; (v) TFA, DCM, r.t.; (vi) HATU, DIPEA, DCM, r.t.

FAP-MB-1: Synthesis of **FAP-MB-1** has been described in the synthesis of compound D.

FAP-MB-2: Synthesis of **FAP-MB-2** by method A using acetic anhydride as reactant to yield a white solid (0.052 g, 48.6%). LC-MS (ESI, *m/z*): calcd. for $C_{33}H_{39}N_6O_5S$ $[M+H]^+$ 631.26, found 631.35; HPLC purity: 98.28%; ^1H NMR (400 MHz, DMSO-d_6) δ 9.97 (s, 1H), 8.01 (t, J = 5.5 Hz, 1H), 7.58 (d, J = 7.7 Hz, 2H), 7.40–7.16 (m, 4H), 6.78–6.55 (m, 4H), 5.10 (s, 2H), 4.49–4.34 (m, 1H), 4.08–3.79 (m, 2H), 3.65–3.40 (m, 2H), 2.88 (s, 12H), 2.21–1.90 (m, 4H), 1.87 (s, 3H); ^{13}C NMR (101 MHz, DMSO-d_6) δ 170.99, 169.90, 167.74, 154.06, 149.07, 139.26, 132.44, 131.44,

128.83, 128.03, 127.47, 119.56, 111.34, 110.19, 67.37, 60.82, 46.48, 41.71, 40.64, 29.81, 24.88, 22.85.

FAP-MB-3: Synthesis of **FAP-MB-3** by method A using propionic anhydride as reactant to yield a white solid (0.063 g, 57.3%). LC-MS (ESI, *m/z*): calcd. for $C_{34}H_{41}N_6O_5S$ $[M+H]^+$ 645.28, found 645.40; HPLC purity: 95.72%; ^{1}H NMR (400 MHz, DMSO-d_6) δ 9.97 (s, 1H), 7.92 (t, J = 5.7 Hz, 1H), 7.58 (d, J = 8.1 Hz, 2H), 7.36–7.17 (m, 4H), 6.75–6.57 (m, 4H), 5.10 (s, 2H), 4.51–4.36 (m, 1H), 4.06–3.92 (m, 2H), 3.92–3.74 (m, 2H), 2.88 (s, 12H), 2.15 (q, J = 7.6 Hz, 2H), 2.08–1.80 (m, 4H), 1.00 (t, J = 7.7 Hz, 3H); ^{13}C NMR (101 MHz, DMSO-d_6) δ 173.66, 171.01, 167.81, 154.07, 149.08, 139.27, 132.44, 131.44, 128.81, 128.03, 127.47, 119.56, 111.34, 110.18, 67.36, 60.83, 46.49, 41.63, 40.64, 29.81, 28.69, 24.88, 10.33.

FAP-MB-4: Synthesis of **FAP-MB-4** by method B using methacrylic acid as reactant to yield a white solid (0.057 g, 50.9%). LC-MS (ESI, *m/z*): calcd. for $C_{35}H_{41}N_6O_5S$ $[M+H]^+$ 657.28, found 657.40; HPLC purity: 96.32%; ^{1}H NMR (400 MHz, DMSO-d_6) δ 9.96 (s, 1H), 8.01 (t, J = 6.0 Hz, 1H), 7.59 (d, J = 8.0 Hz, 2H), 7.40–7.18 (m, 4H), 6.75–6.53 (m, 4H), 5.37 (s, 2H), 5.10 (s, 2H), 4.50–4.36 (m, 1H), 4.12–3.81 (m, 2H), 3.70–3.46 (m, 2H), 2.88 (s, 12H), 2.20–1.92 (m, 4H), 1.88 (s, 3H); ^{13}C NMR (101 MHz, DMSO-d_6) δ 170.99, 168.02, 167.68, 154.07, 149.07, 139.92, 139.26, 132.44, 131.44, 128.83, 128.03, 127.47, 120.08, 119.52, 111.33, 110.18, 67.36, 60.84, 46.53, 41.91, 40.64, 29.80, 24.89, 18.99.

FAP-MB-5: Synthesis of **FAP-MB-5** by method B using *N,N*-dimethylglycine as reactant to yield a white solid (0.072 g, 63.2%). LC-MS (ESI, *m/z*): calcd. for $C_{35}H_{44}N_7O_5S$ $[M+H]^+$ 674.30, found 674.45; HPLC purity: 98.62%; ^{1}H NMR (400 MHz, DMSO-d_6) δ 9.99 (s, 1H), 7.82 (t, J = 5.2 Hz, 1H), 7.58 (d, J = 8.6 Hz, 2H), 7.41–7.19 (m, 4H), 6.80–6.55 (m, 4H), 5.10 (s, 2H), 4.49–4.36 (m, 1H), 4.08–3.88 (m, 2H), 3.67–3.39 (m, 2H), 2.91 (s, 1H), 2.88 (s, 12H), 2.23 (s, 6H), 2.10–1.80 (m, 4H); ^{13}C NMR (101 MHz, DMSO-d_6) δ 170.90, 170.11, 167.44, 154.06, 149.08, 139.25, 132.44, 131.47, 128.86, 128.03, 127.47, 119.56, 111.35, 110.19, 67.36, 63.14, 60.81, 46.38, 46.01, 41.43, 40.65, 29.86, 24.83.

FAP-MB-6: Synthesis of **FAP-MB-6** has been described in the synthesis of compound C.

FAP-MB-7: Synthesis of **FAP-MB-7** by method C using benzyl alcohol as reactant to yield a white solid (0.082 g, 66.7%). LC-MS (ESI, *m/z*): calcd. for $C_{39}H_{43}N_6O_6S$ $[M+H]^+$ 723.29, found 723.40; HPLC purity: 99.21%; ^{1}H NMR (400 MHz, DMSO-d_6) δ 9.99 (s, 1H), 7.58 (d, J = 8.2 Hz,

2H), 7.44–7.11 (m, 9H), 6.78–6.48 (m, 4H), 5.10 (s, 2H), 5.04 (s, 2H), 4.49–4.37 (m, 1H), 4.04–3.71 (m, 2H), 3.71–3.39 (m, 2H), 2.88 (s, 12H), 2.30–1.72 (m, 4H); ^{13}C NMR (101 MHz, DMSO-d_6) δ 170.99, 167.81, 156.97, 154.07, 149.08, 139.29, 137.58, 132.44, 131.43, 128.85, 128.79, 128.21, 128.12, 128.04, 127.47, 119.53, 111.34, 110.19, 67.37, 65.85, 60.81, 46.35, 43.22, 40.64, 29.75, 24.90.

FAP-MB-8: Synthesis of **FAP-MB-8** by method C using 4-pyridylcarbinol as reactant to yield a white solid (0.076 g, 61.8%). LC-MS (ESI, *m/z*): calcd. for $C_{38}H_{42}N_7O_6S$ $[M+H]^+$ 724.28, found 724.35; HPLC purity: 99.97%; ^{1}H NMR (400 MHz, DMSO-d_6) δ 10.01 (s, 1H), 8.58–8.49 (m, 2H), 7.58 (d, J = 8.4 Hz, 2H), 7.41–7.15 (m, 6H), 6.76–6.56 (m, 4H), 5.20–5.00 (m, 4H), 4.52–4.40 (m, 1H), 4.04–3.76 (m, 2H), 3.69–3.41 (m, 2H), 2.88 (s, 12H), 2.20–1.82 (m, 4H); ^{13}C NMR (101 MHz, DMSO-d_6) δ 170.99, 167.69, 156.72, 154.06, 150.06, 149.07, 146.81, 139.29, 132.44, 131.43, 128.86, 128.03, 127.47, 121.93, 119.52, 111.34, 110.19, 67.37, 64.09, 60.81, 46.35, 43.25, 40.64, 29.76, 24.89.

FAP-MB-9: Synthesis of **FAP-MB-9** by method D using Fmoc-L-phenylalanine as reactant to yield a white solid (0.046 g, 36.8%). LC-MS (ESI, *m/z*): calcd. for $C_{40}H_{46}N_7O_5S$ $[M+H]^+$ 736.32, found 736.45; HPLC purity: 96.41%; ^{1}H NMR (400 MHz, DMSO-d_6) δ 10.01 (s, 1H), 8.26 (t, J = 5.3 Hz, 1H), 7.58 (d, J = 8.4 Hz, 2H), 7.42–7.04 (m, 9H), 6.76–6.51 (m, 4H), 5.10 (s, 2H), 4.56–4.40 (m, 1H), 4.12–3.87 (m, 2H), 3.67–3.44 (m, 2H), 3.21–2.93 (m, 1H), 2.88 (s, 12H), 2.68–2.60 (m, 2H), 2.19–1.73 (m, 4H); ^{13}C NMR (101 MHz, DMSO-d_6) δ 173.79, 170.91, 167.32, 154.06, 149.07, 139.25, 138.64, 132.44, 131.48, 129.81, 128.85, 128.68, 128.03, 127.47, 126.74, 119.58, 111.33, 110.19, 67.36, 60.84, 56.01, 46.45, 41.77, 40.64, 29.88, 24.85, 19.57.

FAP-MB-10: Synthesis of **FAP-MB-10** by method B using 3-indolepropionic acid as reactant to yield a white solid (0.055 g, 42.6%). LC-MS (ESI, *m/z*): calcd. for $C_{42}H_{46}N_7O_5S$ $[M+H]^+$ 760.32, found 760.40; HPLC purity: 99.90%; ^{1}H NMR (400 MHz, DMSO-d_6) δ 10.73 (s, 1H), 9.94 (s, 1H), 8.07 (t, J = 5.5 Hz, 1H), 7.64–7.46 (m, 3H), 7.35–7.23 (m, 4H), 7.11 (s, 1H), 7.08–6.92 (m, 3H), 6.70–6.61 (m, 4H), 5.09 (s, 2H), 4.51–4.35 (m, 1H), 4.12–3.80 (m, 2H), 3.63–3.47 (m, 2H), 2.93 (t, J = 7.8 Hz, 2H), 2.88 (s, 12H), 2.57–2.51 (m, 2H), 2.19–1.73 (m, 4H); ^{13}C NMR (101 MHz, DMSO-d_6) δ 172.68, 171.00, 167.84, 154.07, 149.08, 139.27, 136.70, 132.44, 128.83, 128.04, 127.51, 127.47, 122.60, 121.32, 119.58, 118.76, 118.58, 114.28, 111.74, 111.34, 110.19, 67.37, 60.85, 46.53, 41.78, 40.64, 36.39, 29.83, 24.88, 21.42.

Synthesis and characterization of N-FAP-MB

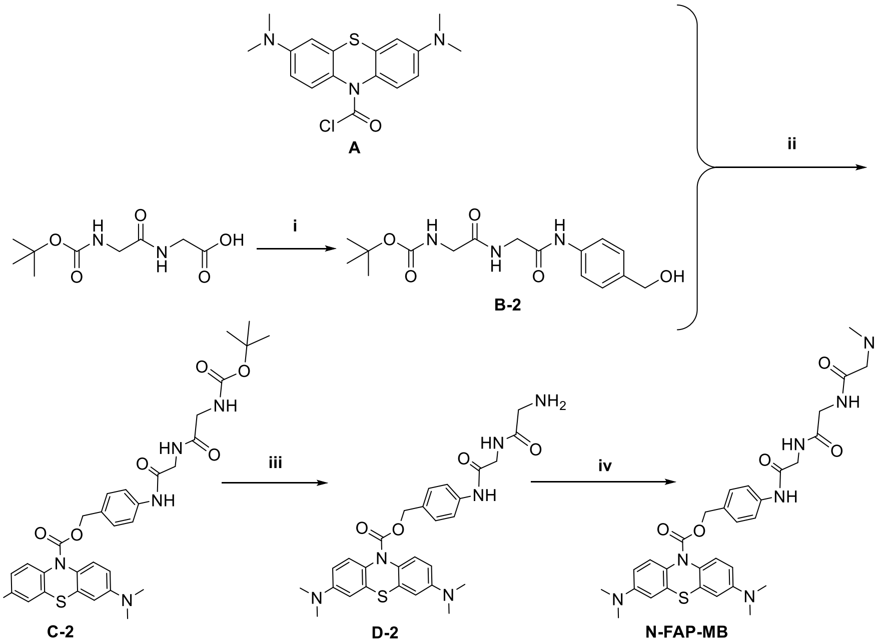

Scheme S2. Synthesis route of compound N-FAP-MB. Reagents and conditions: (i) *p*-Aminobenzyl alcohol, HATU, DIPEA, DMF, r.t.; (ii) Na_2CO_3, DMAP, DCM-MeCN (1:1), reflux.; (iii) TFA, DCM, r.t.; (iv) HATU, DIPEA, DCM, r.t.

Synthesis of compound B-2

Compound B-2 was synthesized according to the procedure of synthesis of compound B using 2-[[2-[(2-methylpropan-2-yl)oxycarbonylamino]acetyl]amino]acetic acid (Boc-Gly-Gly-OH) as reactant to yield a yellow solid (1.106 g, 76.1%). LC-MS (ESI, *m/z*): calcd. for $C_{16}H_{22}N_3O_5$ $[M-H]^-$ 336.16, found 336.15.

Synthesis of compound C-2

Compound B-2 (0.4 g, 0.33 mmol), Compound A (0.453 g, 0.37 mmol), DMAP (0.159 g, 0.37 mmol), Na_2CO_3 (0.377 g, 1 mmol) were added to a mixed solvent of 15 mL of MeCN and 15 mL of DCM. The mixture was refluxed until the reaction was completed as monitored by TLC analysis. The reaction mixture was filtered and concentrated under reduced pressure, the residue was dissolved in DCM (20 mL), and successively washed with water (2×10 mL), 0.2 M HCl (2×10 mL) and brine (2×10 mL). The organic layer was dried over anhydrous Na_2SO_4, filtered and evaporated on a rotary evaporator. The crude product was purified by column chromatography using DCM/MeOH (25: 1) as eluent to yield **compound C-2** as white solid (0.4 g, 52.02%). LC-MS (ESI, *m/z*): calcd. for $C_{33}H_{41}N_6O_6S$ $[M+H]^+$ 649.27, found 649.30; HPLC purity: 99.57%; ^1H NMR (500

MHz, DMSO-d_6) δ 9.88 (s, 1H), 8.17 (t, *J* = 5.8 Hz, 1H), 7.59 (d, *J* = 8.2 Hz, 2H), 7.37–7.24 (m, 4H), 7.09 (t, *J* = 6.1 Hz, 1H), 6.80–6.56 (m, 4H), 5.10 (s, 2H), 3.90 (d, *J* = 5.7 Hz, 2H), 3.61 (d, *J* = 6.0 Hz, 2H), 2.88 (s, 12H), 1.39 (s, 9H).

Synthesis of compound D-2

Compound D-2 was synthesized according to the procedure of synthesis of compound D using compound C-2 as reactant to yield a white solid (0.085 g, 51.3%). LC-MS (ESI, *m/z*): calcd. for $C_{28}H_{33}N_6O_4S$ $[M+H]^+$ 549.22, found 549.30; HPLC purity: 98.92%; ^1H NMR (400 MHz, DMSO-d_6) δ 10.01 (s, 1H), 8.20 (t, 1H), 7.57 (d, *J* = 8.1 Hz, 2H), 7.40–7.13 (m, 4H), 6.78–6.52 (m, 4H), 5.10 (s, 2H), 3.94 (d, 2H), 3.17 (t, 2H), 2.88 (s, 12H); ^{13}C NMR (101 MHz, DMSO-d_6) δ 173.72, 168.24, 154.07, 149.08, 139.04, 132.45, 131.53, 128.87, 128.04, 127.47, 119.53, 111.34, 110.19, 67.36, 45.07, 42.94, 40.65.

Synthesis of N-FAP-MB.

Synthesis of **N-FAP-MB** by method B using compound D-2 and *N,N*-dimethylglycine as reactants to yield a white solid (0.042 g, 72.5%). LC-MS (ESI, m/z): calcd. for $C_{32}H_{40}N_7O_5S$ $[M+H]^+$ 634.27, found 634.35; HPLC purity: 98.76%; ^1H NMR (400 MHz, DMSO-d_6) δ 10.00 (s, 1H), 8.32 (t, *J* = 5.9 Hz, 1H), 8.16 (t, *J* = 5.8 Hz, 1H), 7.61 (d, *J* = 8.1 Hz, 2H), 7.42–7.23 (m, 4H), 6.79–6.54 (m, 4H), 5.10 (s, 2H), 3.91 (d, *J* = 5.8 Hz, 2H), 3.82 (d, *J* = 5.8 Hz, 2H), 3.08 (s, 2H), 2.88 (s, 12H), 2.32 (s, 6H); ^{13}C NMR (101 MHz, DMSO-d_6) δ 169.94, 169.65, 168.13, 154.07, 149.08, 139.04, 132.44, 131.55, 128.83, 128.03, 127.48, 119.52, 111.34, 110.19, 67.35, 62.38, 45.68, 43.18, 42.34, 40.65.

Supporting Figures

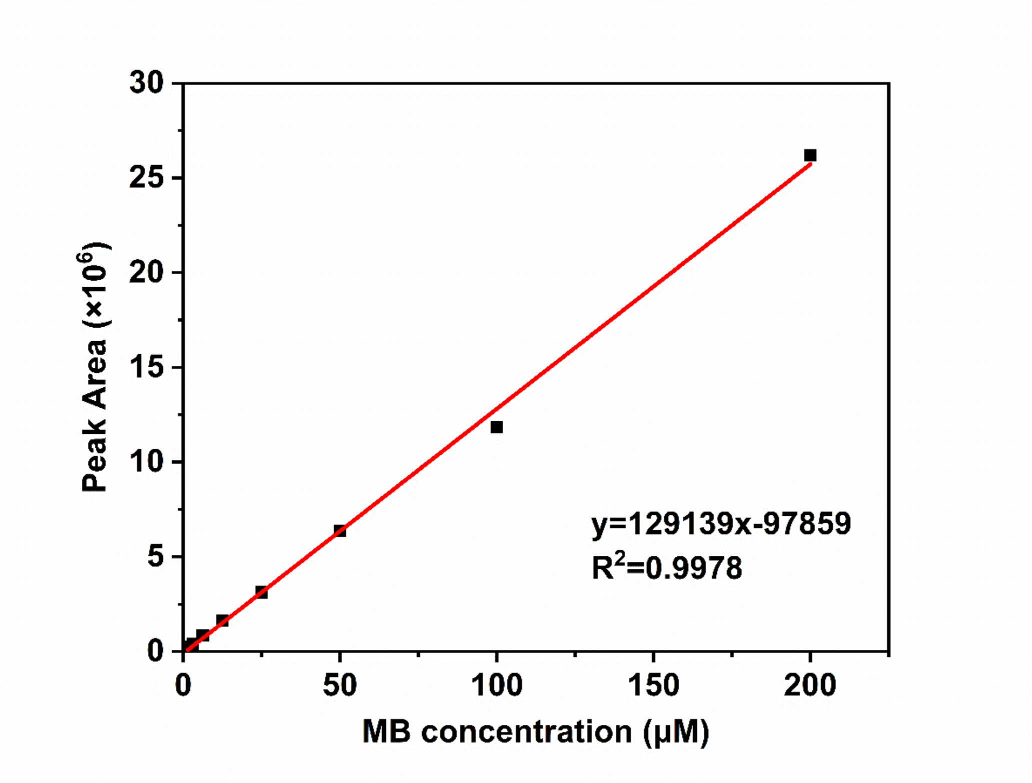

Figure S1. Linear relationship between various concentrations of MB (1.5−200 μM) and peak area at 665 nm *via* HPLC.

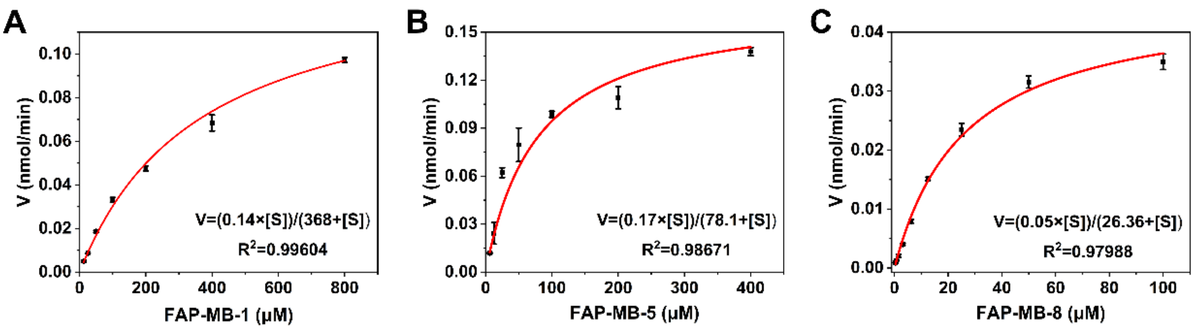

Figure S2. Kinetics studies of FAP-MB-1 (A), FAP-MB-5 (B) and FAP-MB-8 (C) towards FAPα.

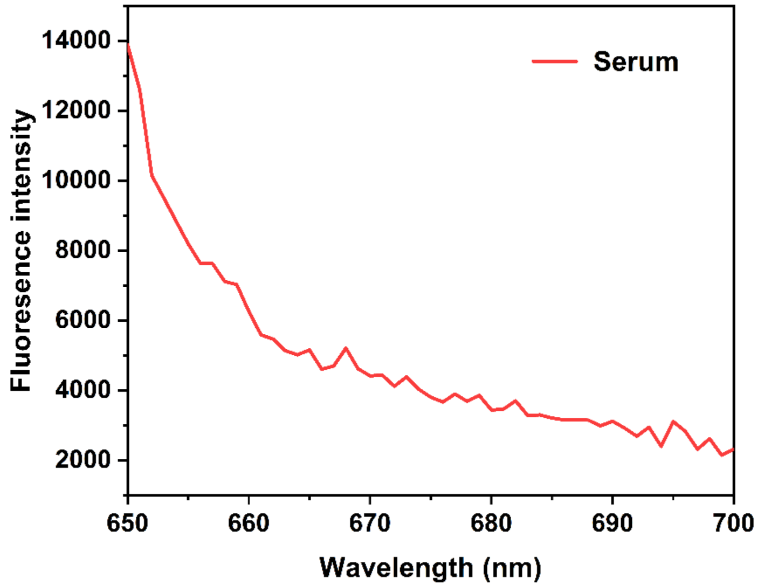

Figure S3. The fluorescence intensity of serum.

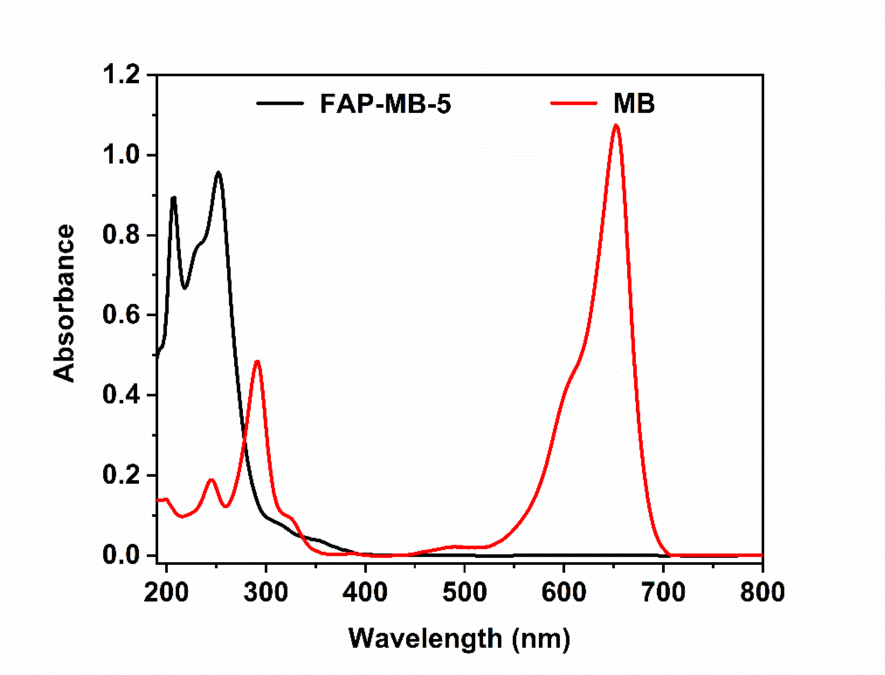

Figure S4. UV-vis absorption spectra of FAP-MB-5 and MB in MeOH.

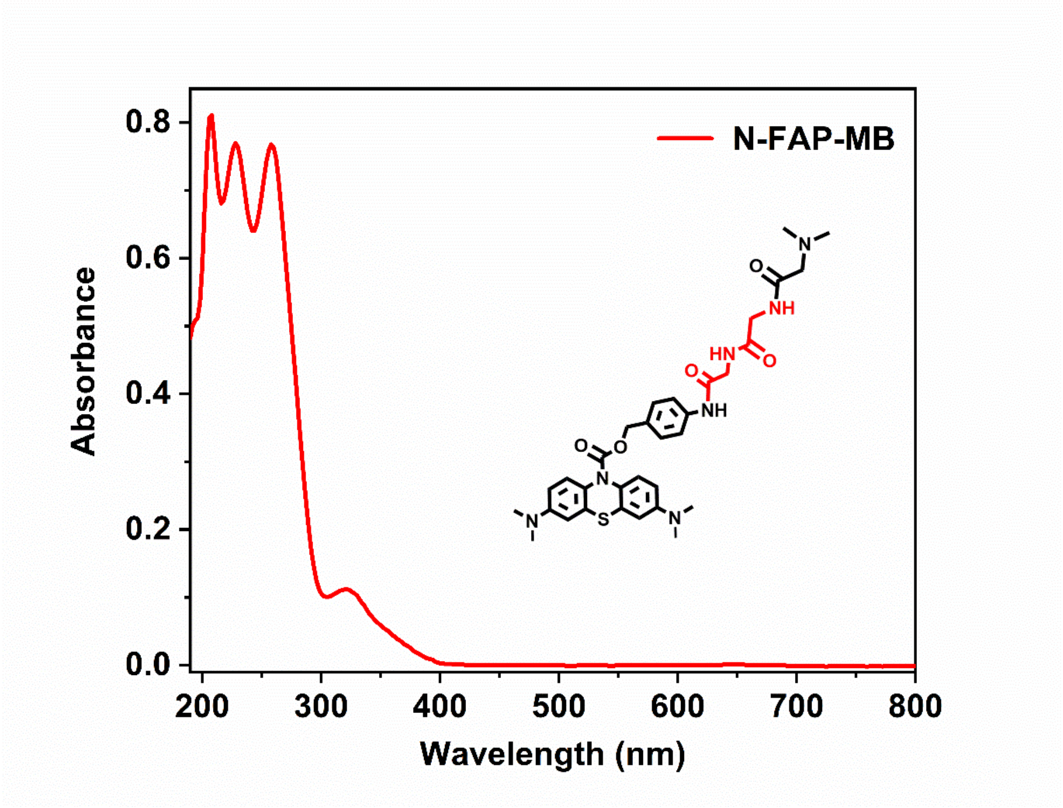

Figure S5. UV-vis absorption spectra of N-FAP-MB in MeOH.

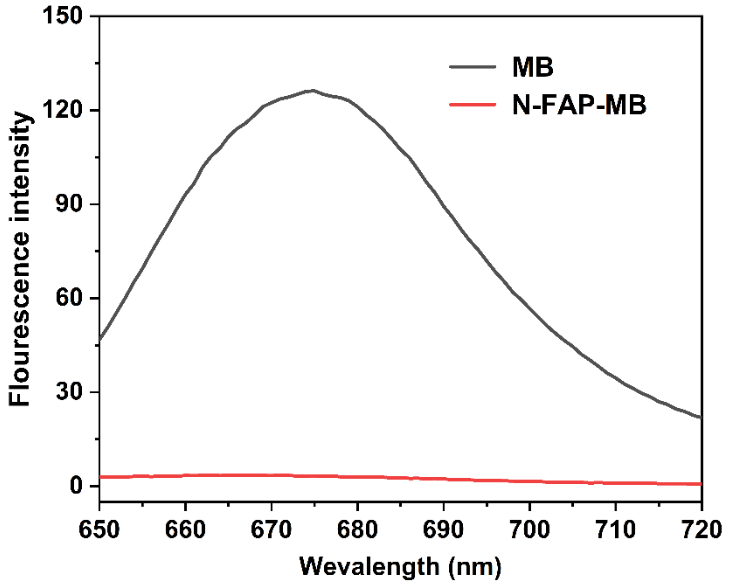

Figure S6. Fluorescence emission of N-FAP-MB and MB in MeOH.

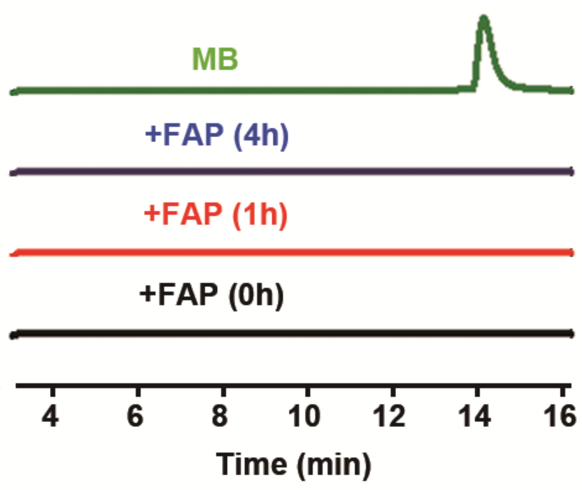

Figure S7. HPLC analysis for FAPα-mediated hydrolysis of N-FAP-MB at different time points.

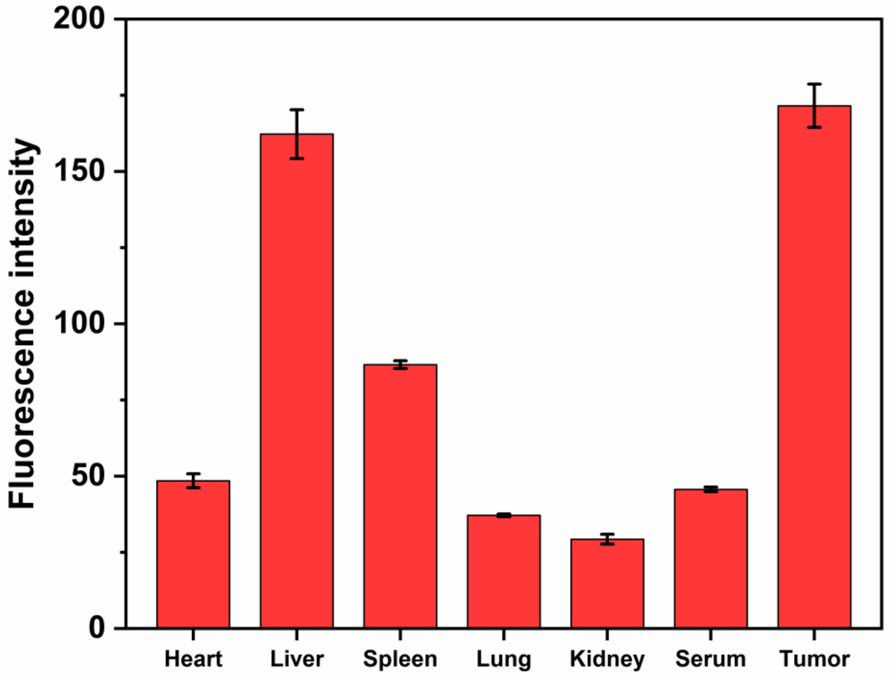

Figure S8. The fluorescence intensity of FAP-MB-5 incubated with serum and major organ homogenate for 4h.

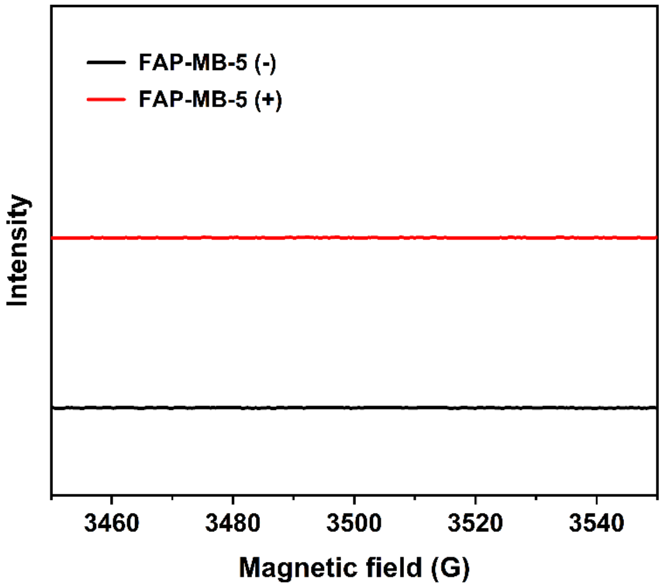

Figure S9. ESR spectra of FAP-MB-5 with or without irradiation. (+) and (–) refer to the treatment with or without irradiation, respectively.

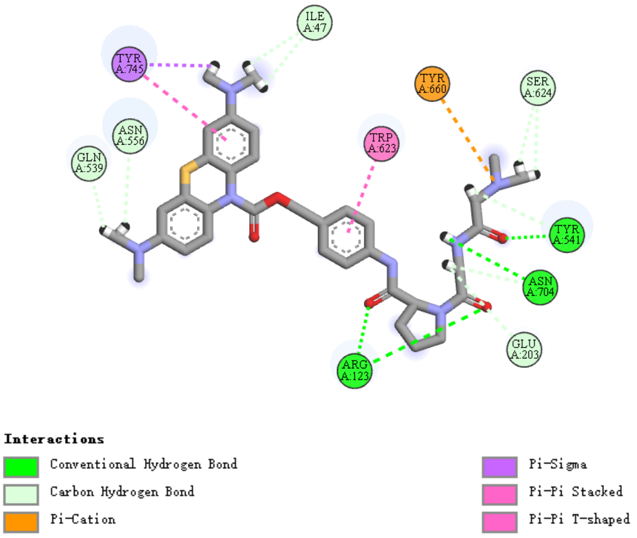

Figure S10. Details of the interaction of FAP-MB-5 with FAPα in a two-dimensional view.

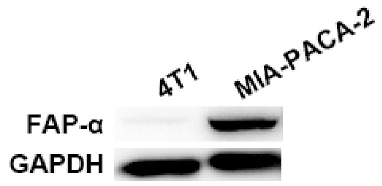

Figure S11. Western blot analysis of FAPα expression in 4T1 and Mia-paca-2 cells.

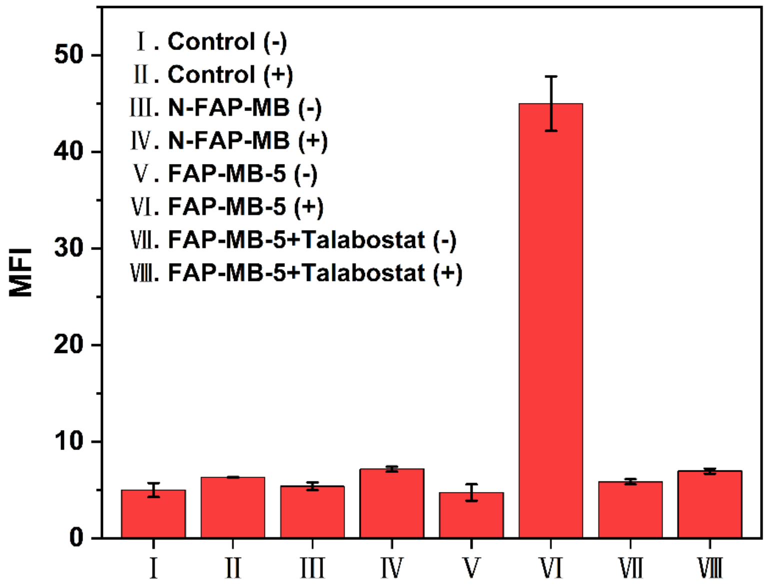

Figure S12. The corresponding mean fluorescence intensity of ROS generation by flow cytometric analysis with various treatments. (+) and (–) refer to the treatment with or without irradiation, respectively.

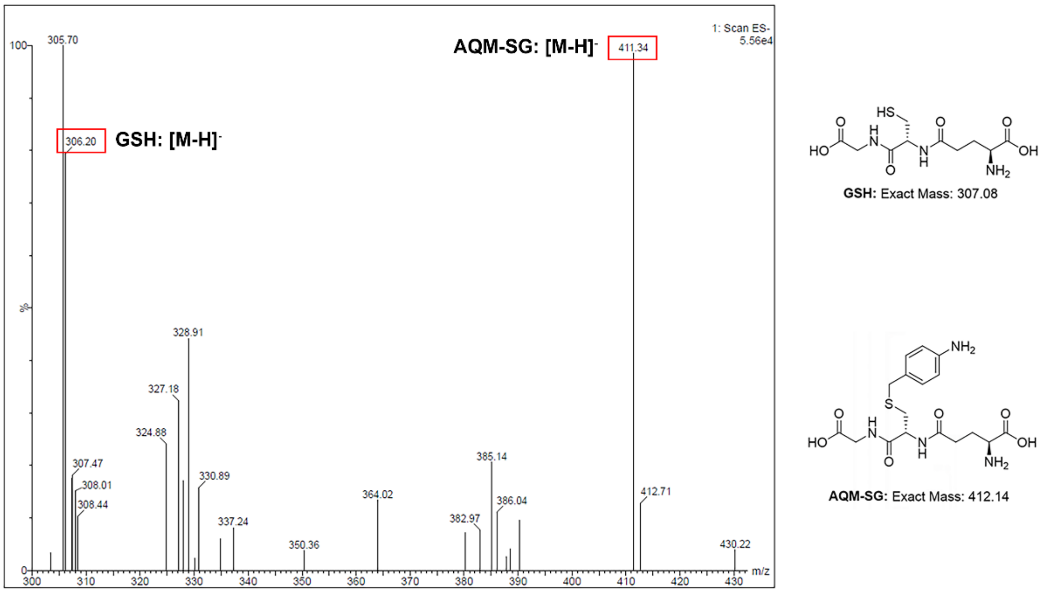

Figure S13. LC-MS spectrum of AQM-SG. AQM-SG was product of nucleophilic addition between AQM and GSH.

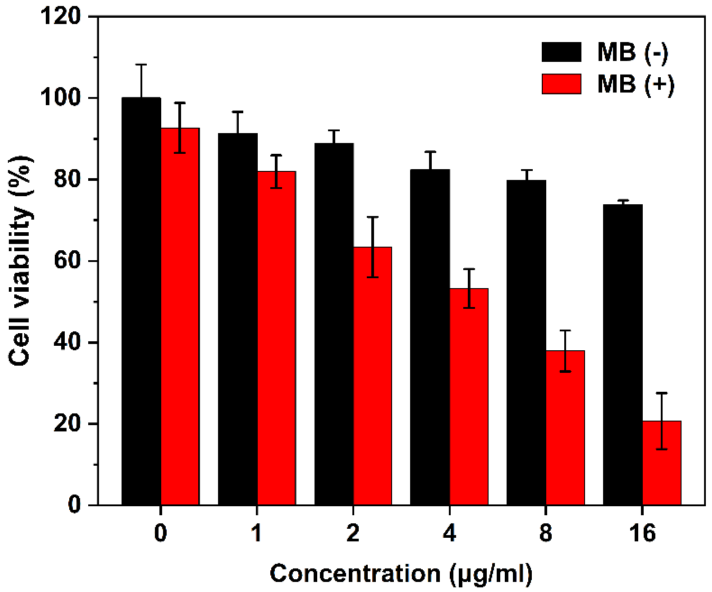

Figure S14. The cell viability of Mia-paca-2 cells treated with MB for 24 h (633 nm, 100 mW/cm^2, 5 min). (+) and (–) refer to the treatment with or without irradiation, respectively.

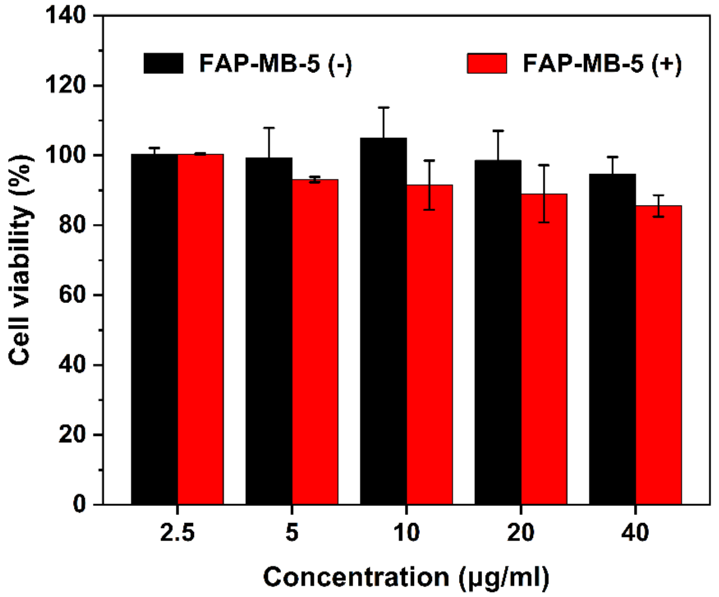

Figure S15. The cell vibility of FAP-MB-5 against 3T3 cells under dark or irradiation (633 nm, 100 mW/cm^2, 5 min). (+) and (–) refer to the treatment with or without irradiation, respectively.

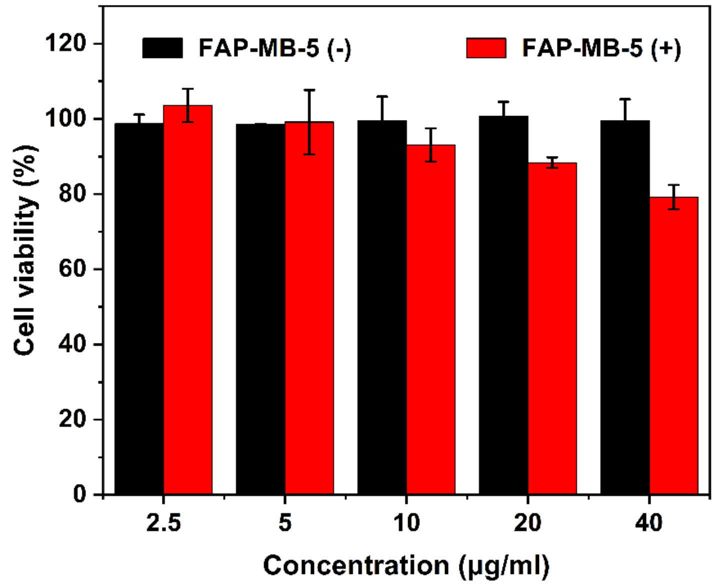

Figure S16. The cell vibility of FAP-MB-5 against 4T1 cells under dark or irradiation (633 nm, 100 mW/cm^2, 5 min). (+) and (–) refer to the treatment with or without irradiation, respectively.

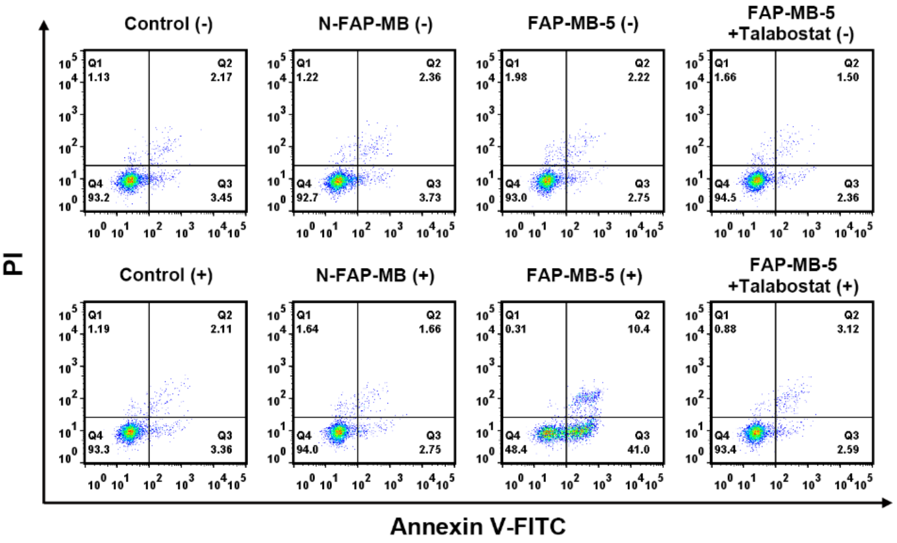

Figure S17. Annexin V-FITC/PI assay of Mia-paca-2 cells treated with FAP-MB-5 and N-FAP-MB (40 μg/mL) under irradiation (633 nm, 100 mW/cm^2, 5 min). (+) and (–) refer to the treatment with or without irradiation, respectively.

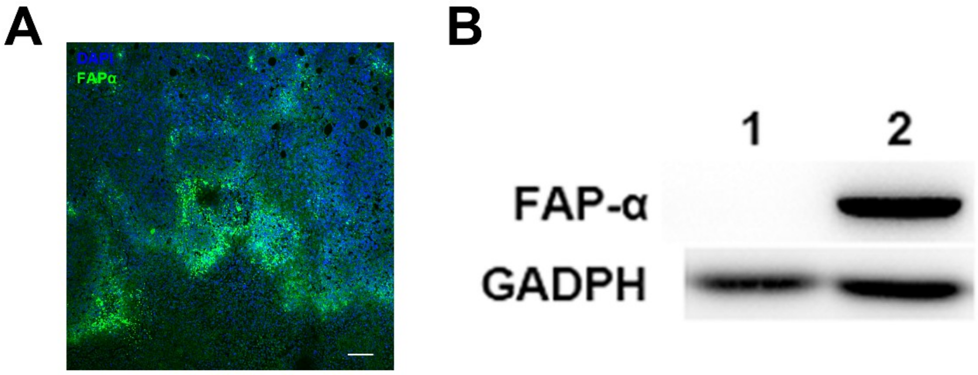

Figure S18. (A) Immunofluorescence analysis of FAPα expression in 4T1 xenograft tissues. Scale bar: 100 μm. (B) Western blot analysis of FAPα expression in normal tissue lysate (1) and 4T1 xenograft lysate (2).

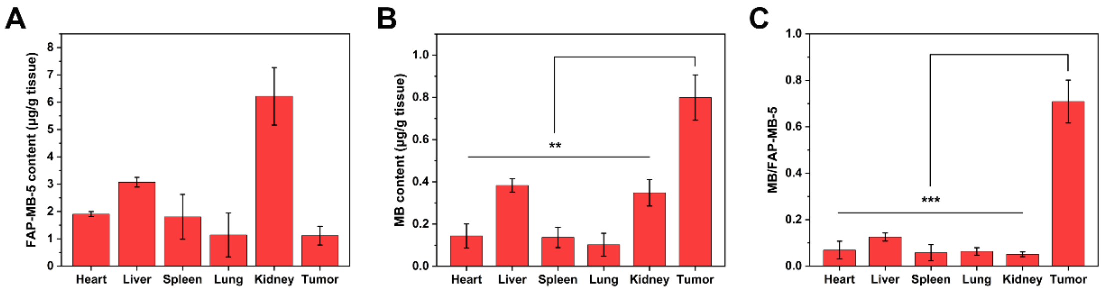

Figure S19. The content of the original FAP-MB-5 (**A**) detected by HPLC and the released MB (**B**) determined by fluorescence spectrofluorometer in tumor and major organs at 4 h post-injection. (**C**) The content of MB/FAP-MB-5 ratios in tumor and major organs at 4 h post-injection.

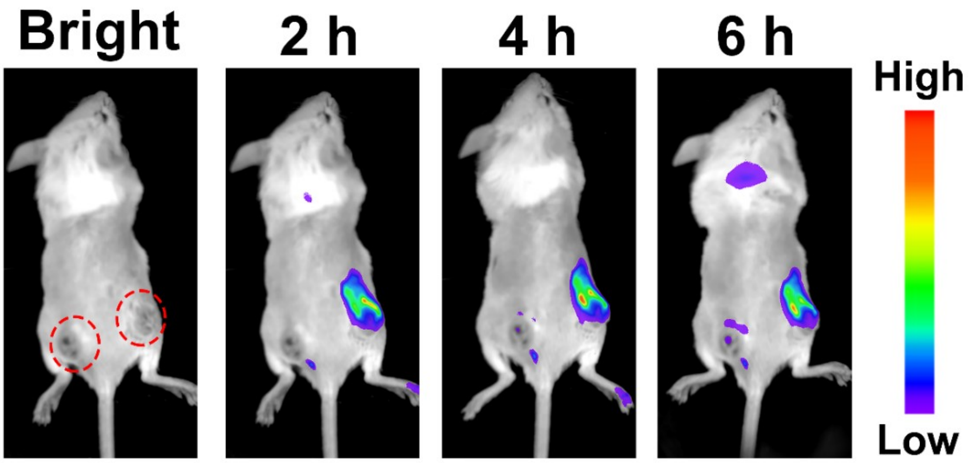

Figure S20. *In vivo* fluorescence images of 4T1 tumor-bearing mice after intratumoral injection of FAP-MB-5 (7 mg/kg, right tumor) and N-FAP-MB (7 mg/kg, left tumor), respectively.

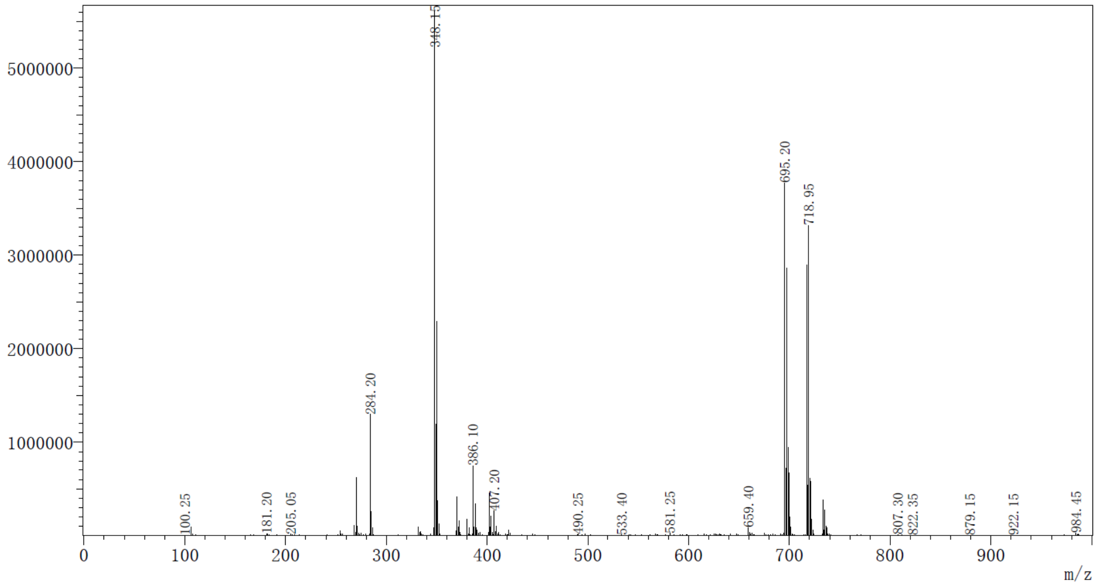

Figure S21. MS spectrum of compound A.

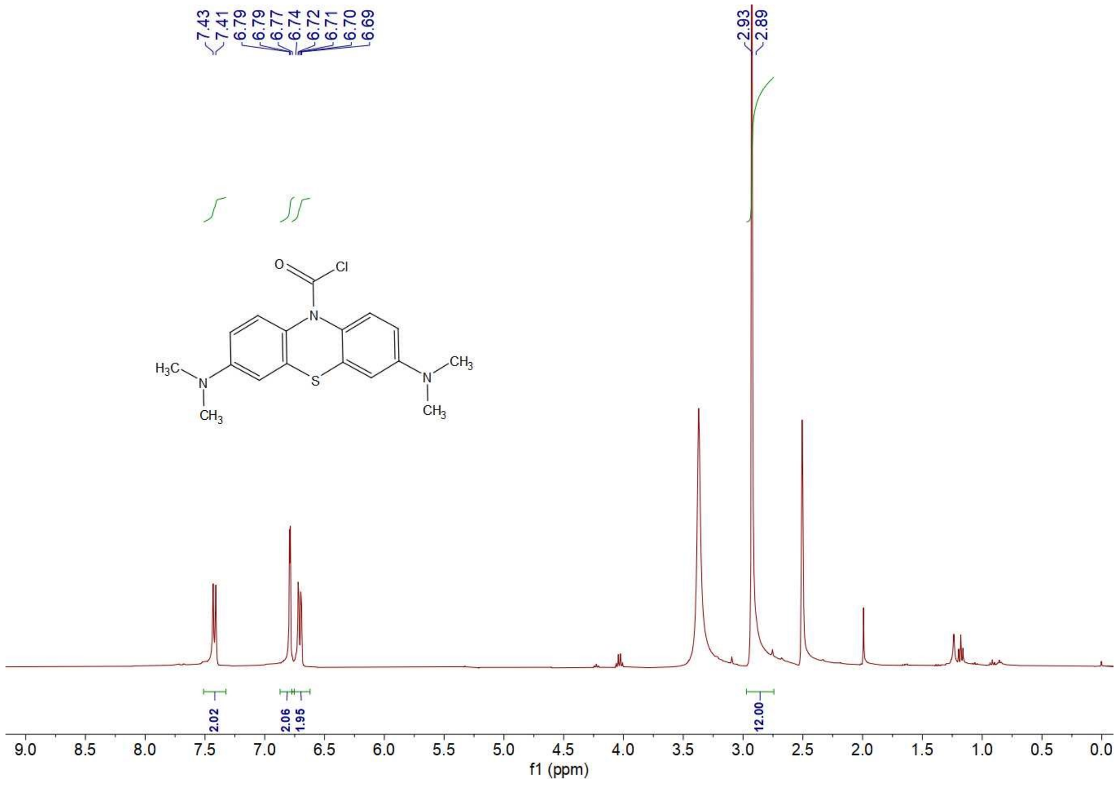

Figure S22. ^{1}H-NMR spectrum of compound A.

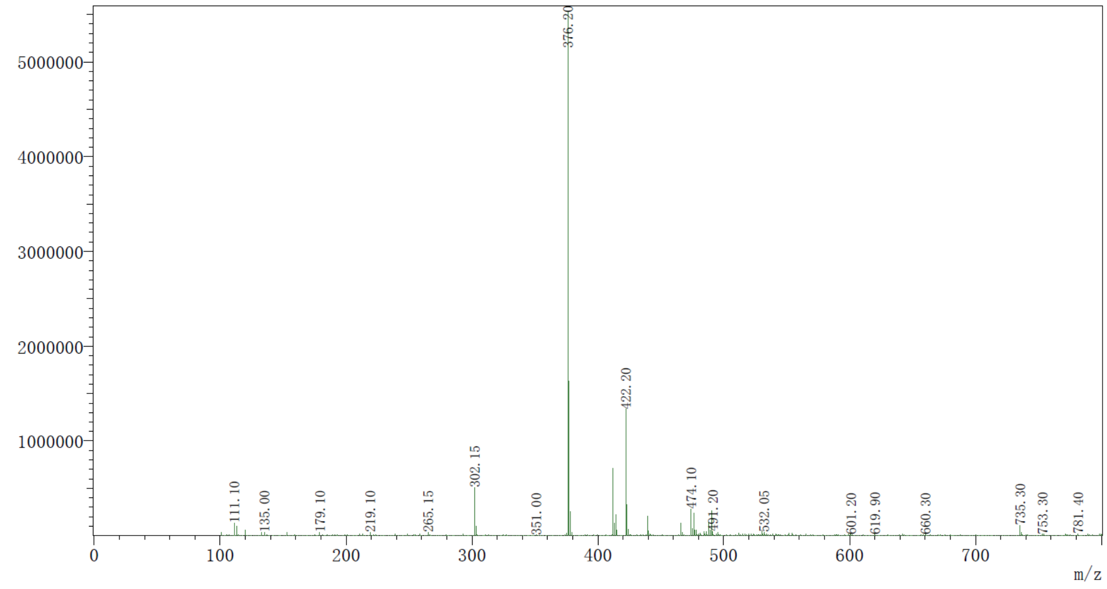

Figure S23. MS spectrum of compound B.

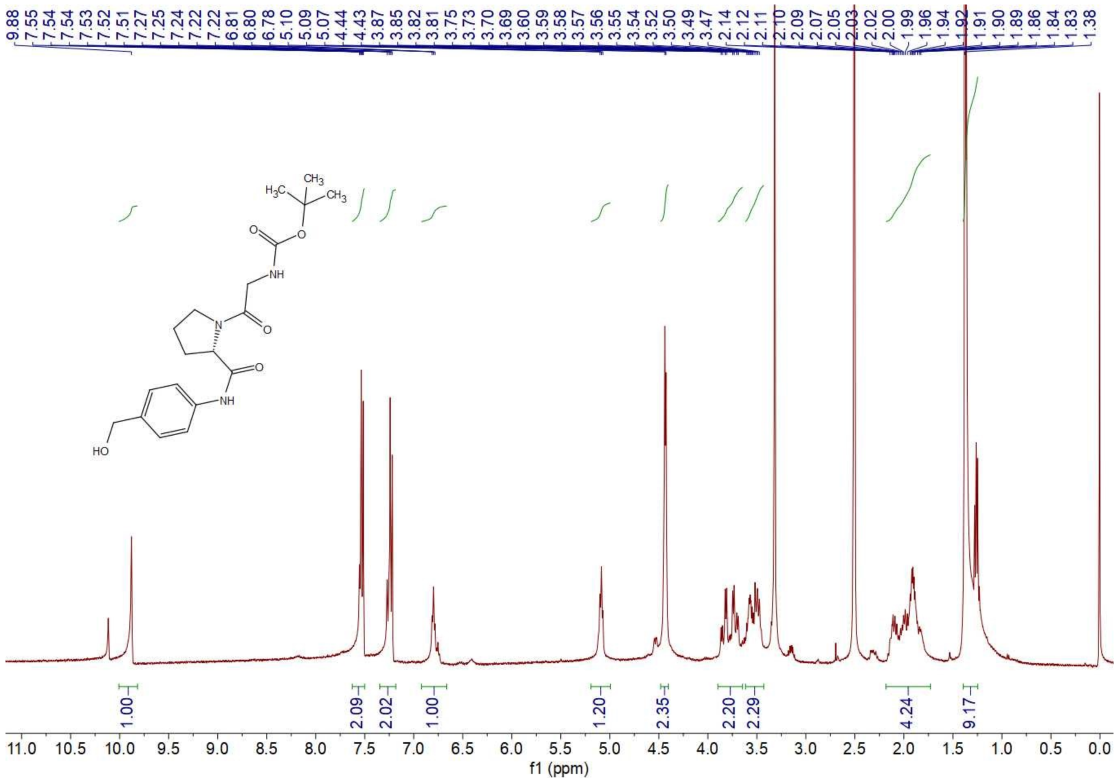

Figure S24. ^{1}H-NMR spectrum of compound B.

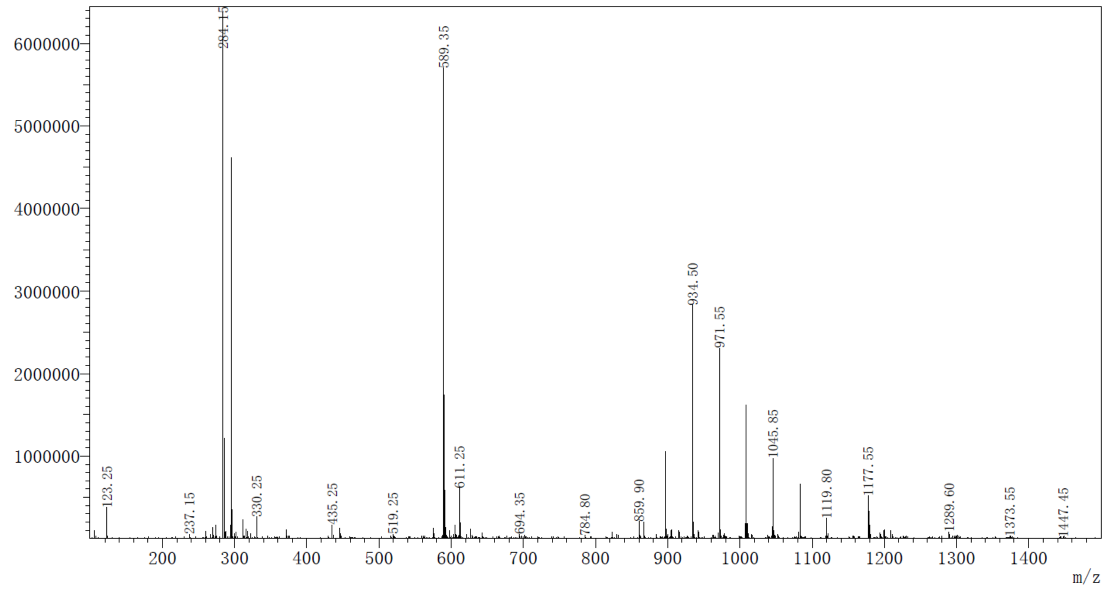

Figure S25. MS spectrum of FAP-MB-1.

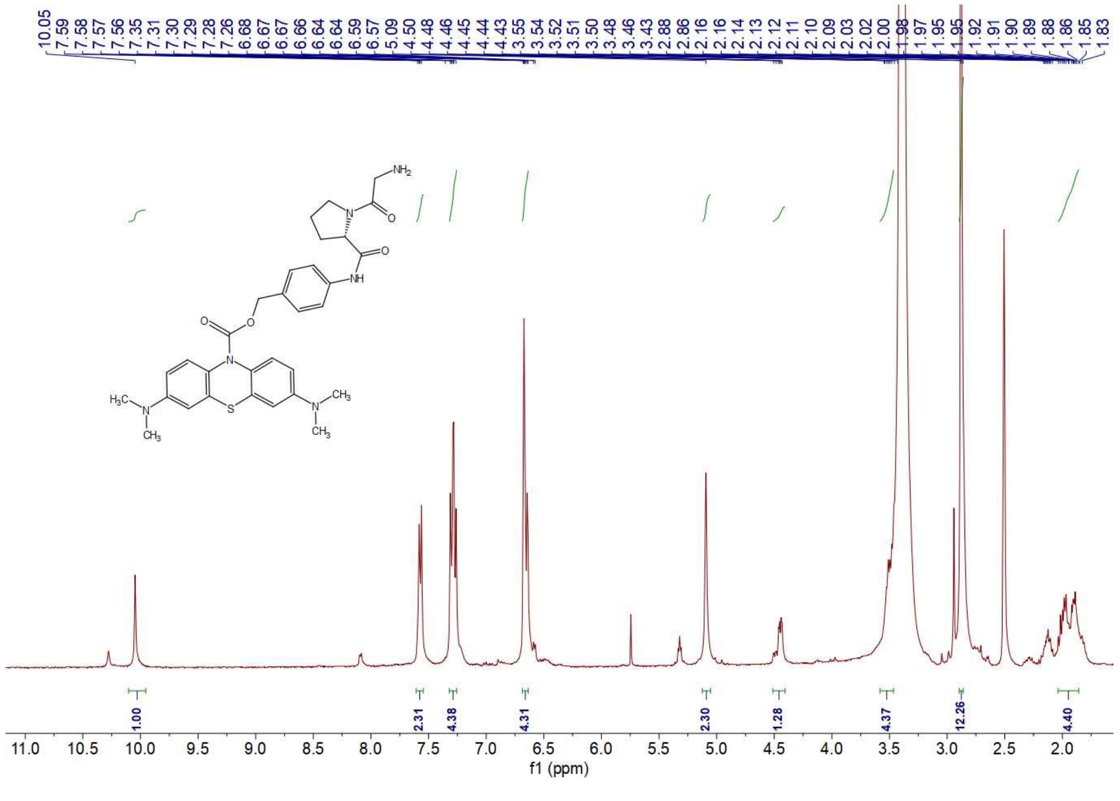

Figure S26. ^{1}H-NMR spectrum of FAP-MB-1.

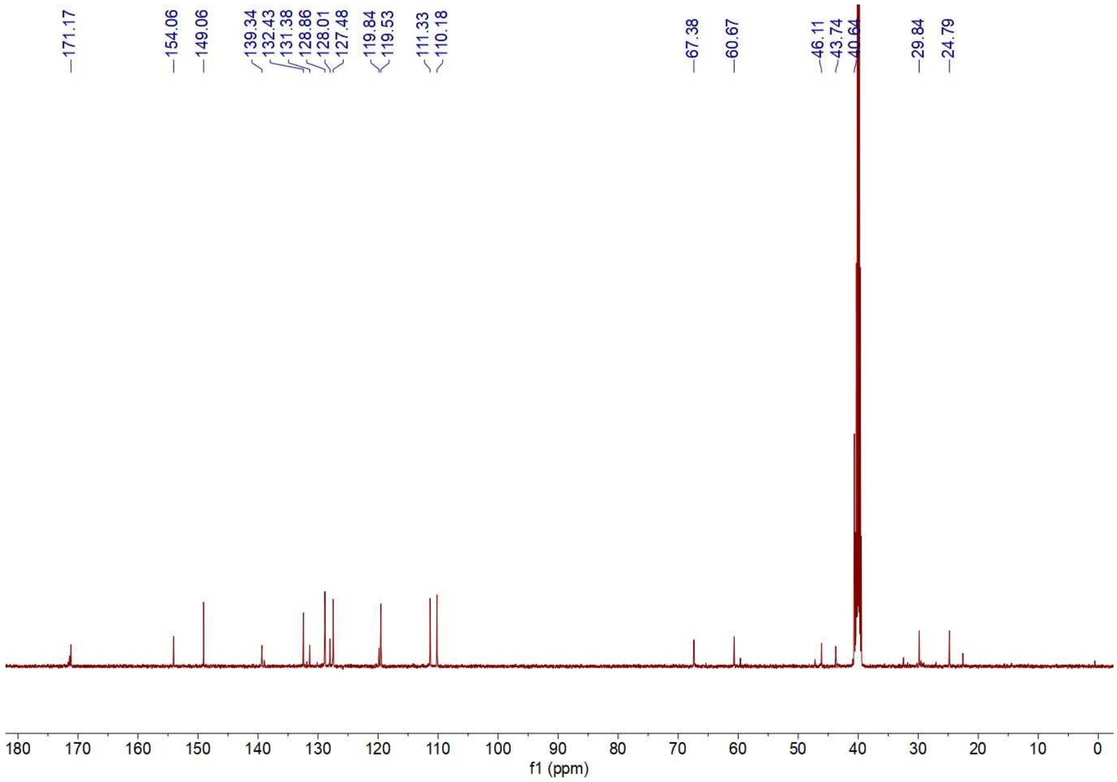

Figure S27. ^{13}C-NMR spectrum of FAP-MB-1.

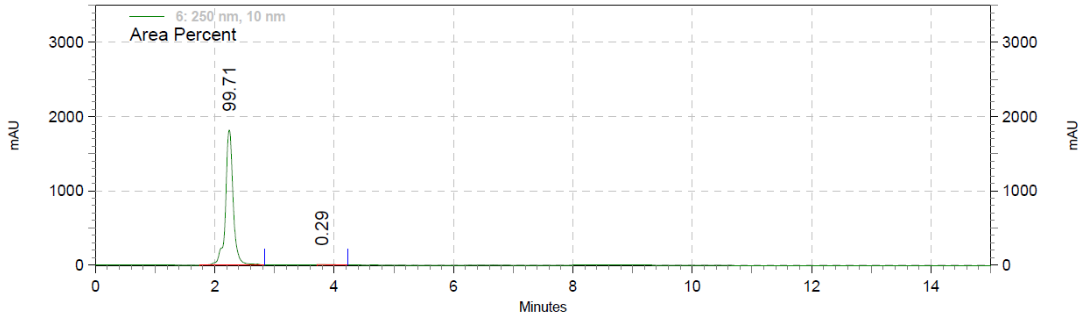

6: 250 nm, 10 nm Results

Pk #	Retention Time	Height	Height %	Area	Area %
**1**	2.24	7280485	99.90	65088381	99.71
**2**	3.79	7640	0.10	188115	0.29
**Totals**		7288125	100.00	65276496	100.00

Figure S28. HPLC data of FAP-MB-1.

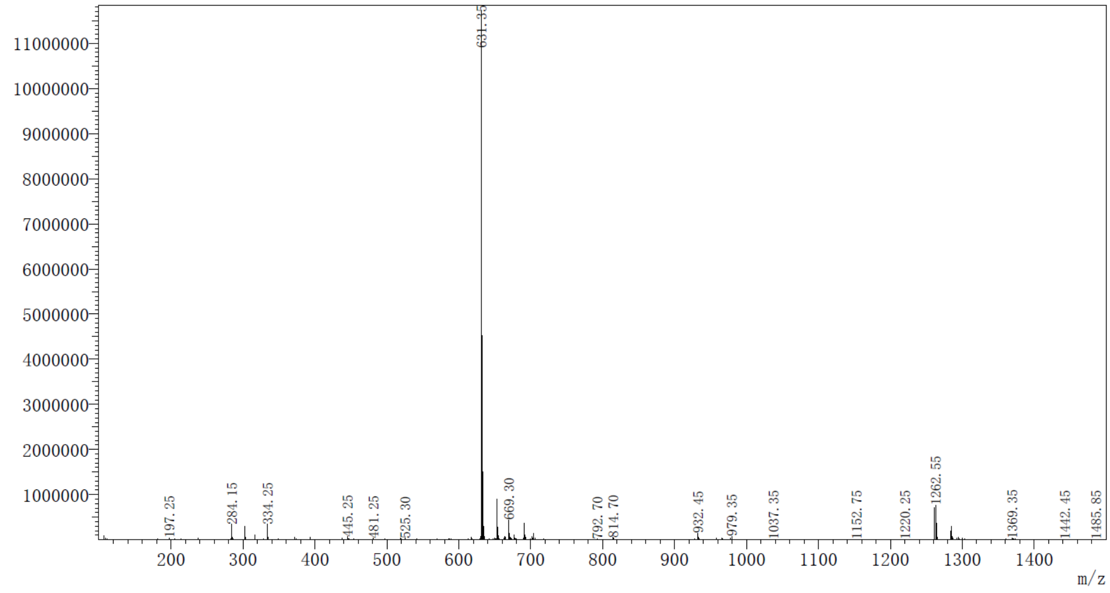

Figure S29. MS spectrum of FAP-MB-2.

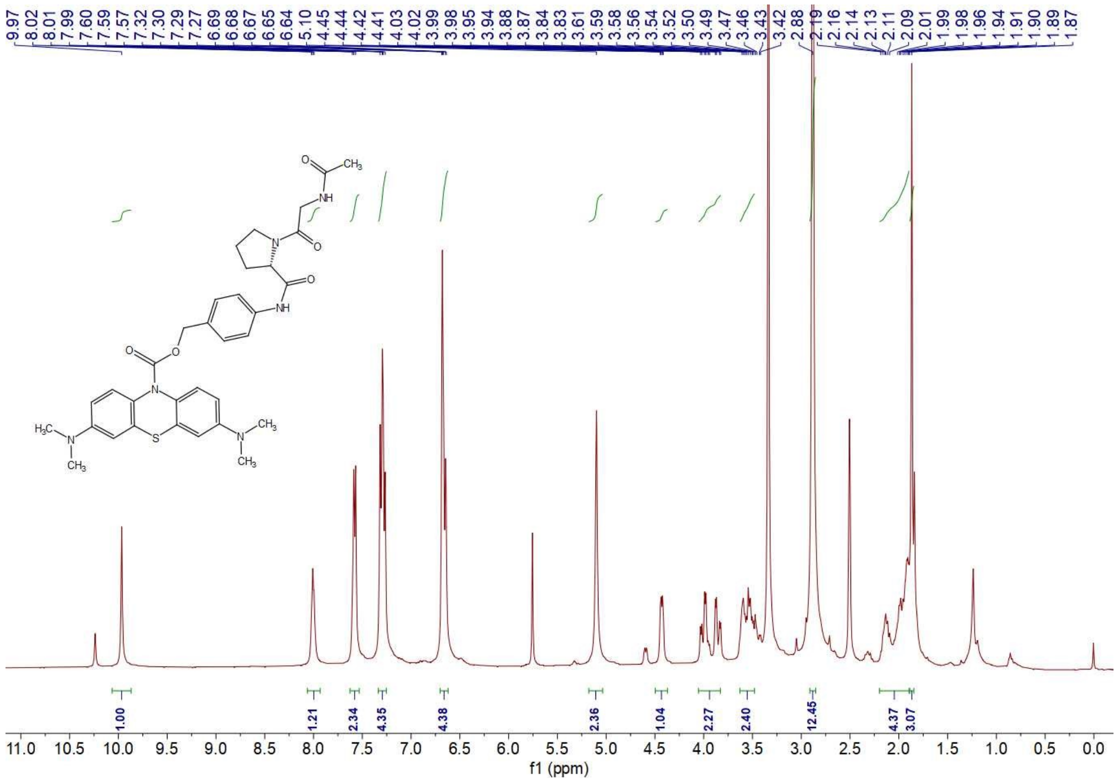

Figure S30. ^{1}H-NMR spectrum of FAP-MB-2.

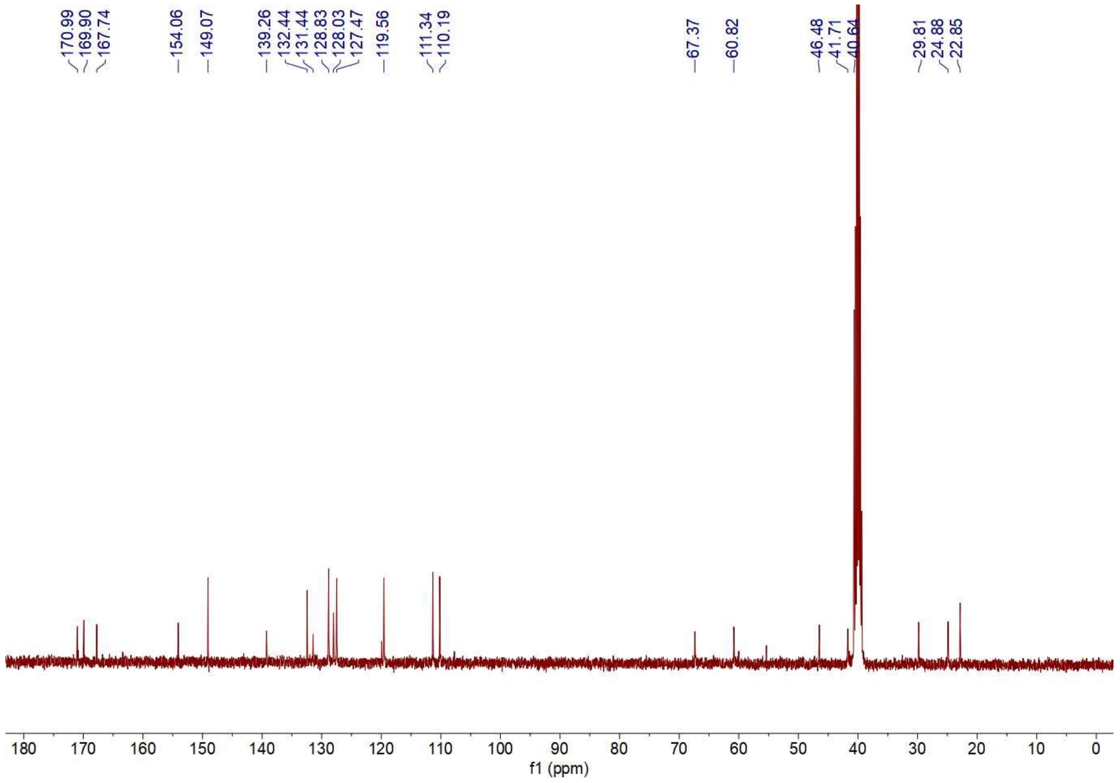

Figure S31. ^{13}C-NMR spectrum of FAP-MB-2.

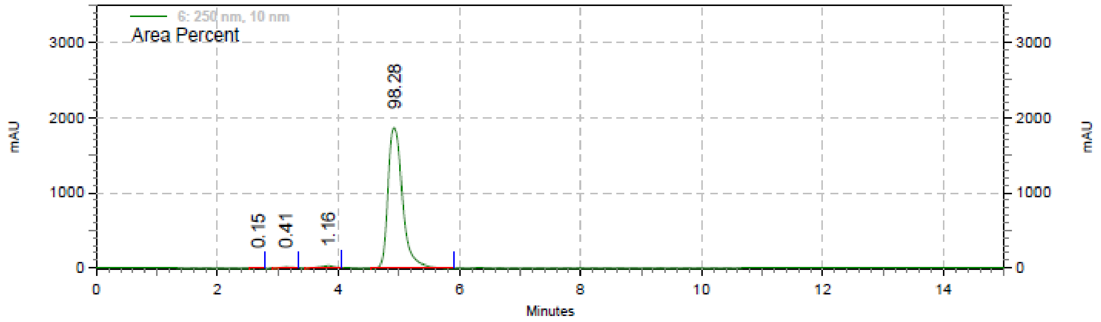

6: 250 nm, 10 nm Results

Pk #	Retention Time	Height	Height %	Area	Area %
1	2.67	26767	0.35	184844	0.15
2	3.13	40093	0.53	511948	0.41
3	3.83	100643	1.32	1459523	1.16
4	4.92	7433717	97.80	123544729	98.28
Totals		7601220	100.00	125701044	100.00

Figure S32. HPLC data of FAP-MB-2.

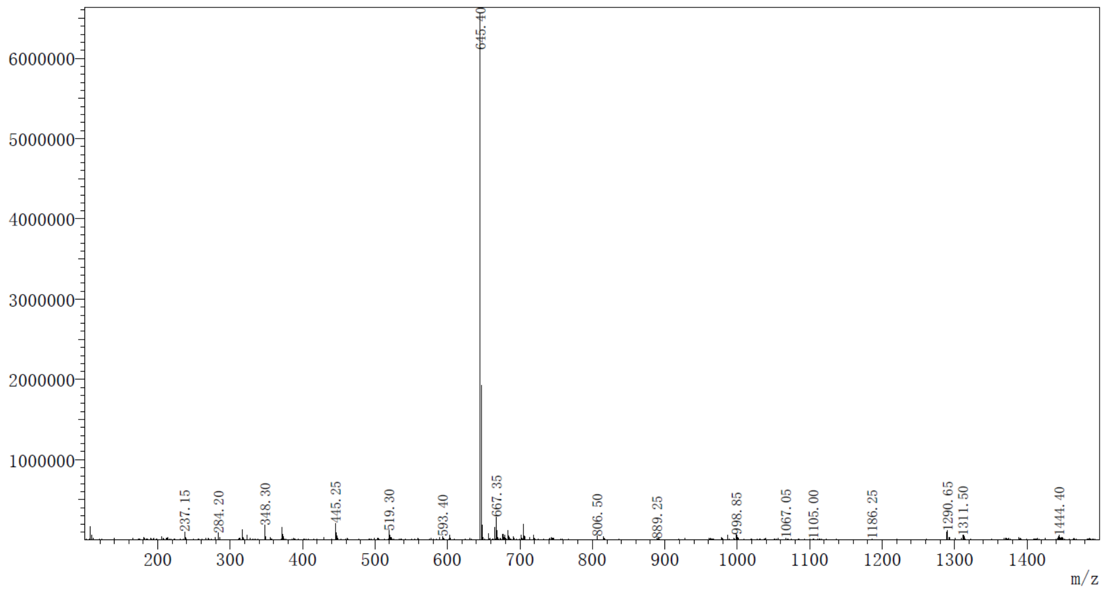

Figure S33. MS spectrum of FAP-MB-3.

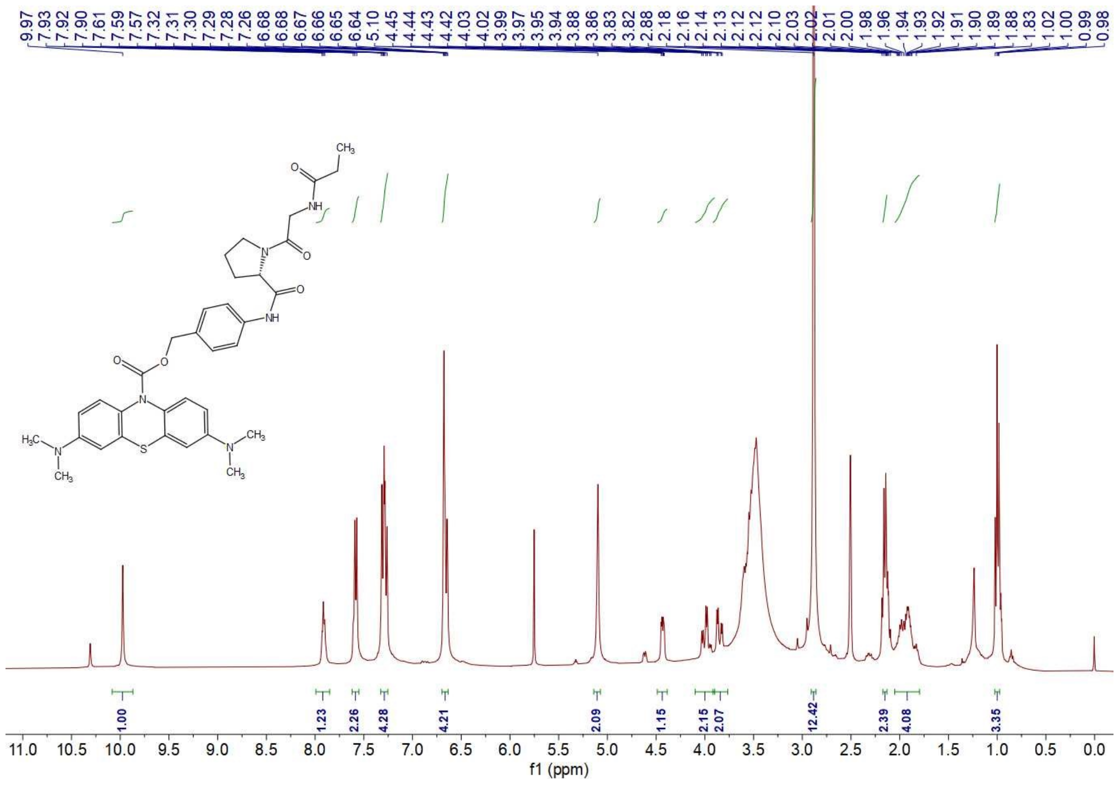

Figure S34. ^{1}H-NMR spectrum of FAP-MB-3.

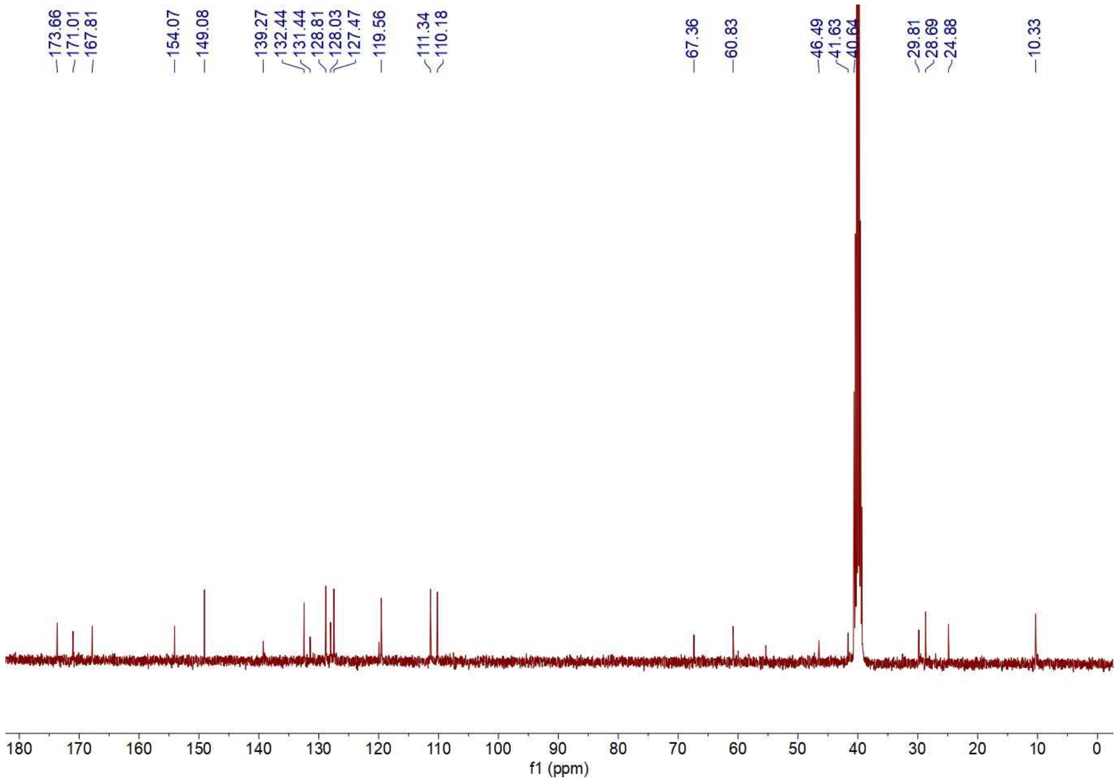

Figure S35. ^{13}C-NMR spectrum of FAP-MB-3.

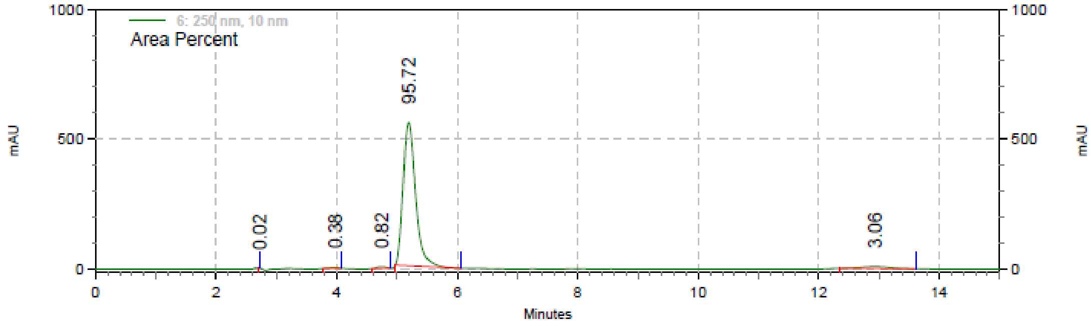

6: 250 nm, 10 nm Results

Pk #	Retention Time	Height	Height %	Area	Area %
1	2.72	4093	0.18	5722	0.02
2	3.97	11123	0.49	125986	0.38
3	4.74	26686	1.17	268975	0.82
4	5.19	2205159	96.84	31481215	95.72
5	12.93	30111	1.32	1006172	3.06
**Totals**		2277172	100.00	32888070	100.00

Figure S36. HPLC data of FAP-MB-3.

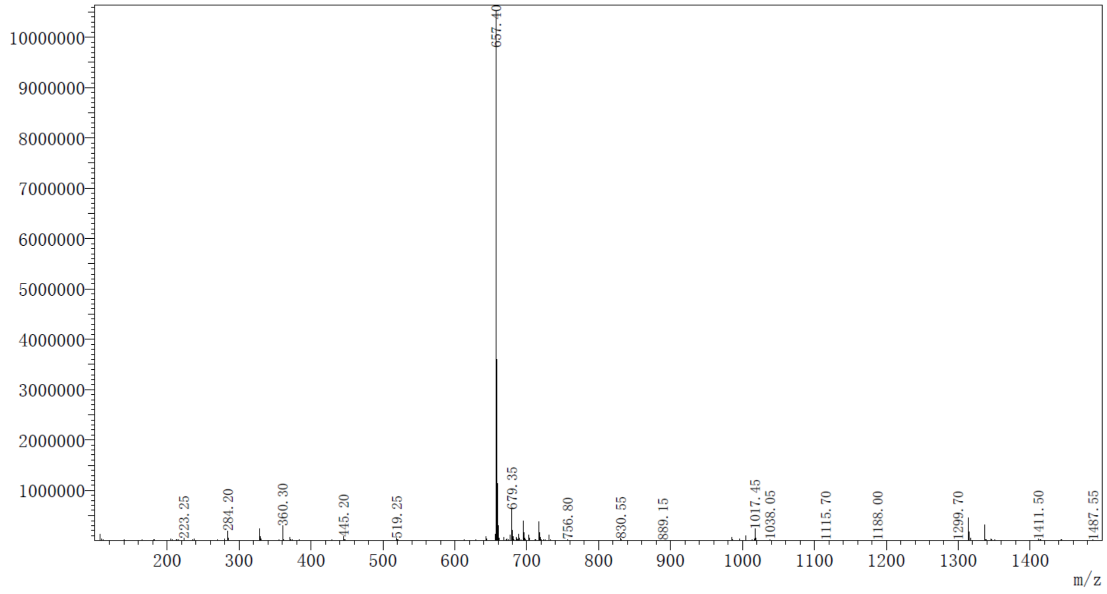

Figure S37. MS spectrum of FAP-MB-4.

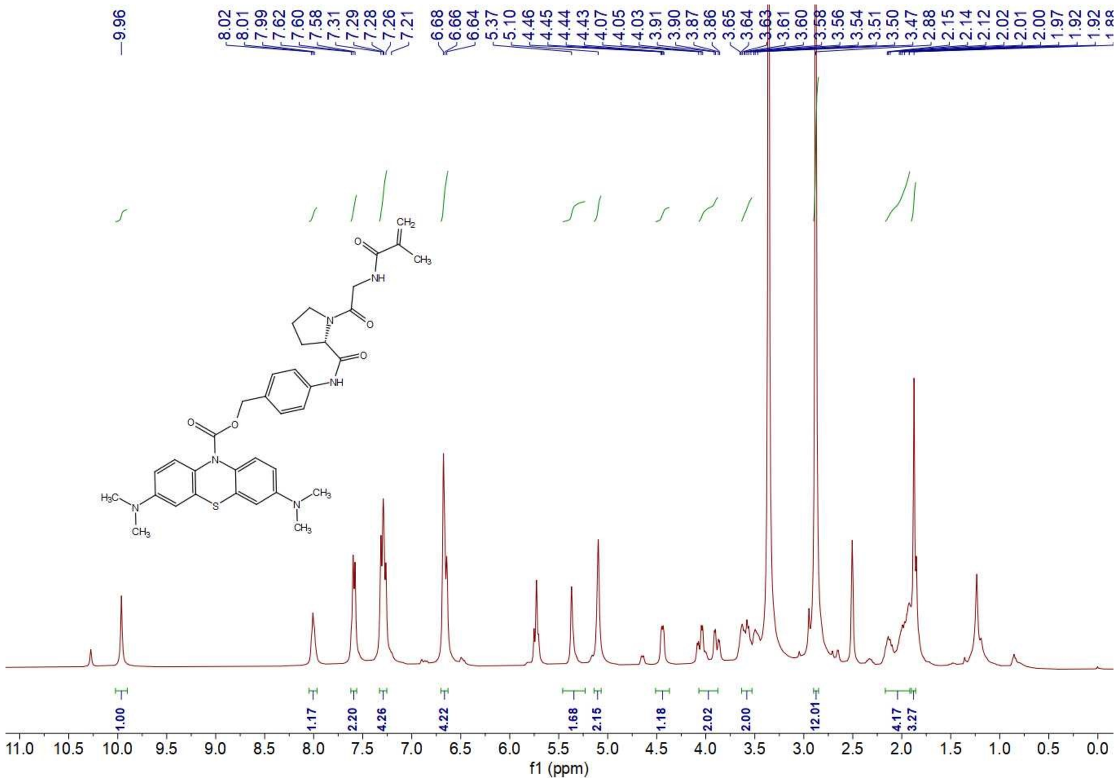

Figure S38. ^{1}H-NMR spectrum of FAP-MB-4.

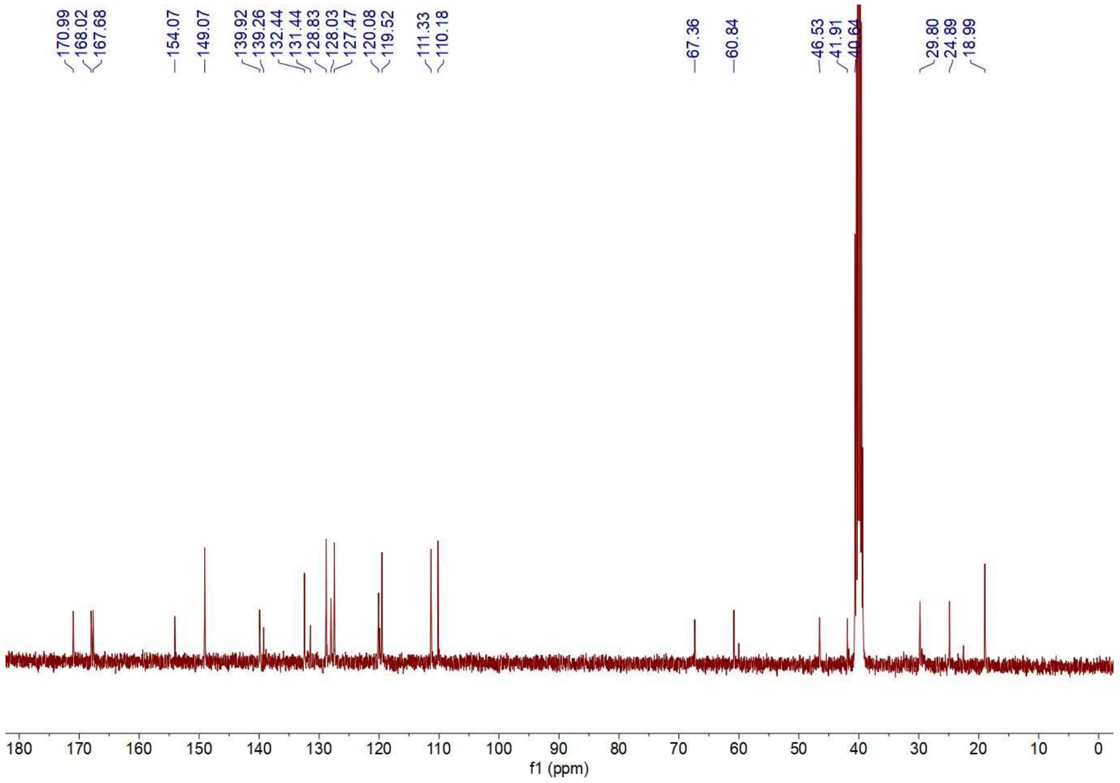

Figure S39. ^{13}C-NMR spectrum of FAP-MB-4.

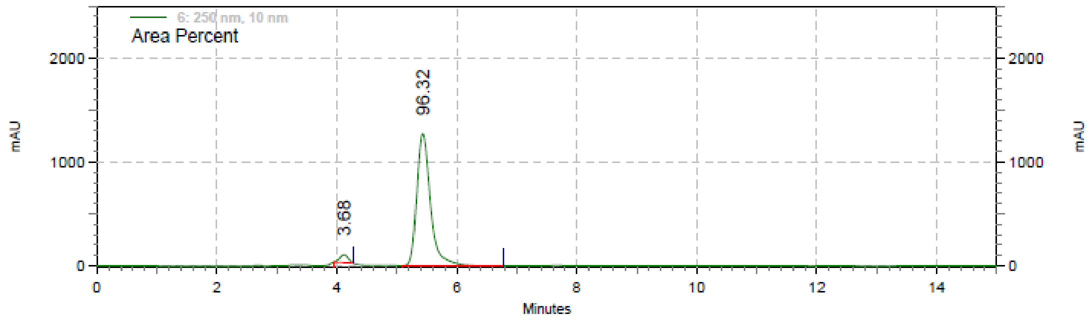

6: 250 nm, 10 nm Results

Pk #	Retention Time	Height	Height %	Area	Area %
1	4.12	306210	5.69	3073125	3.68
2	5.43	5076444	94.31	80379896	96.32
**Totals**		5382654	100.00	83453021	100.00

Figure S40. HPLC data of FAP-MB-4.

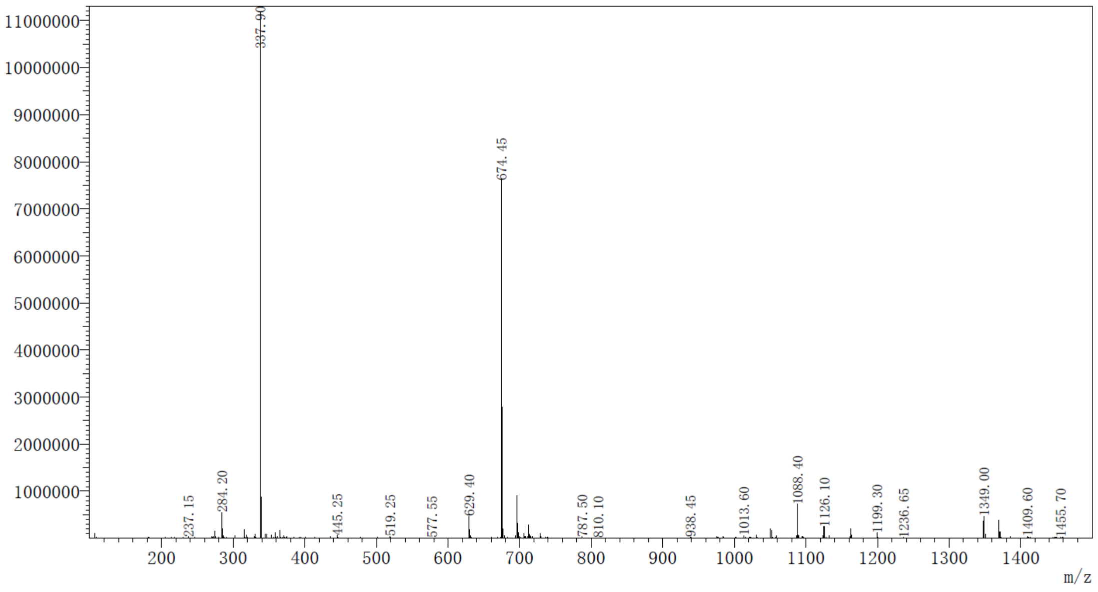

Figure S41. MS spectrum of FAP-MB-5.

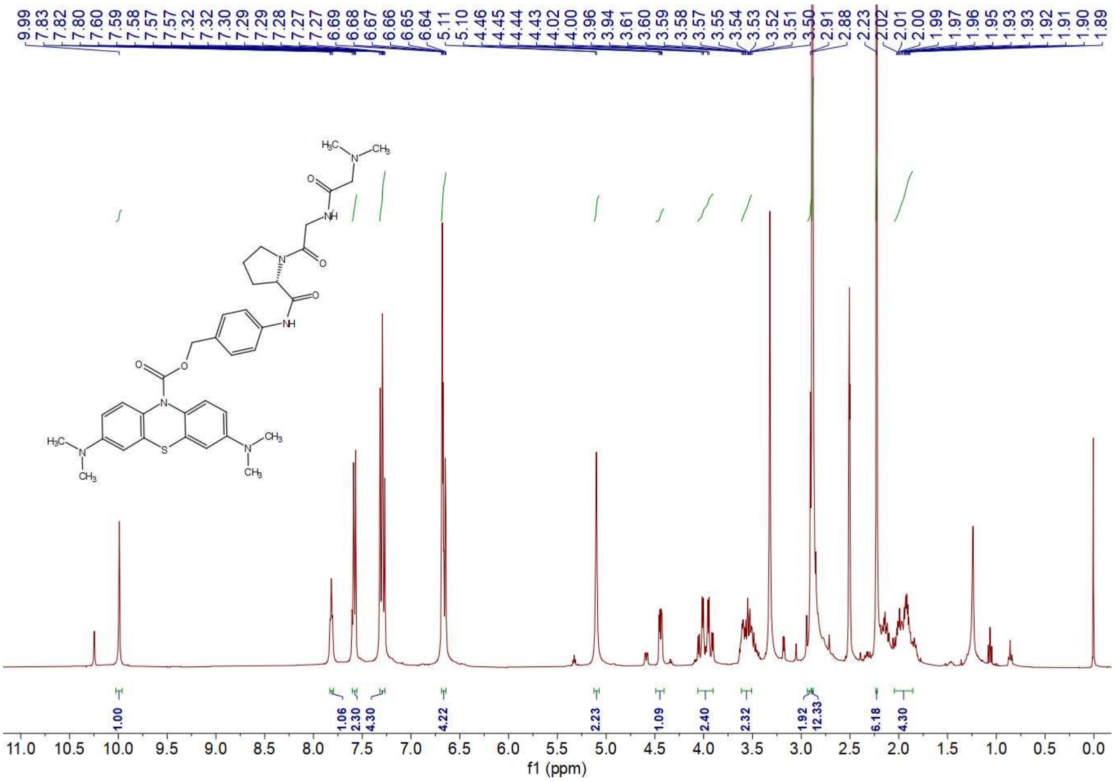

Figure S42. ^{1}H-NMR spectrum of FAP-MB-5.

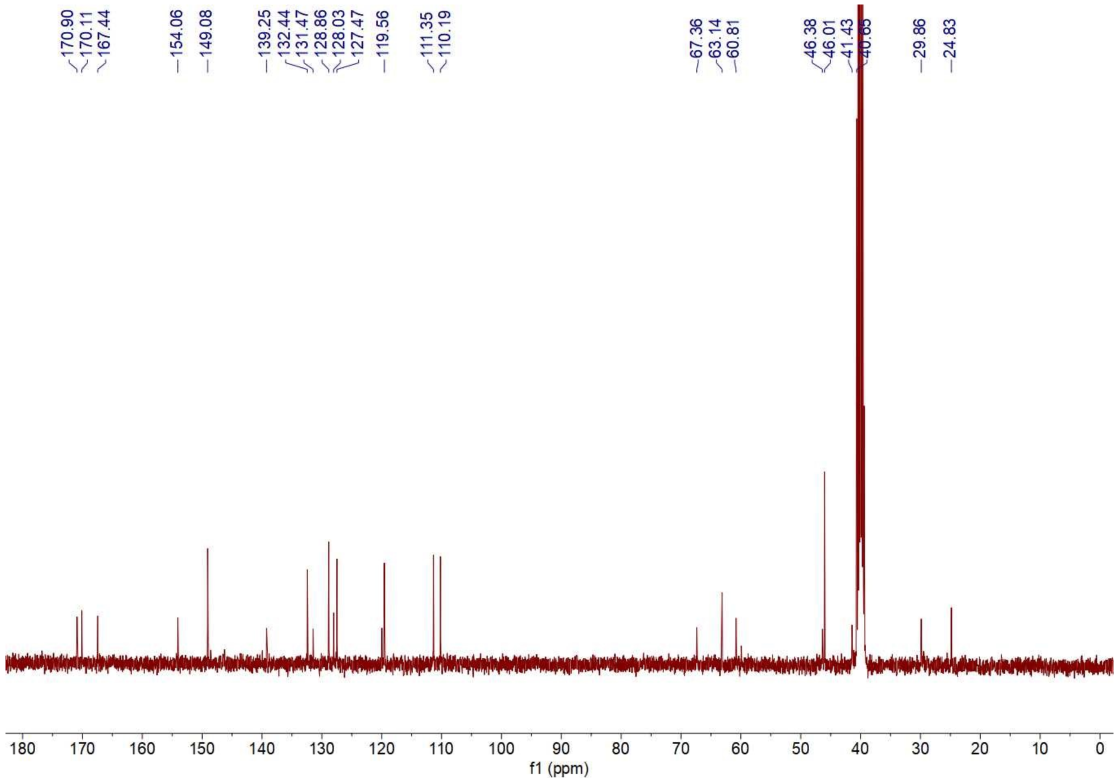

Figure S43. ^{13}C-NMR spectrum of FAP-MB-5.

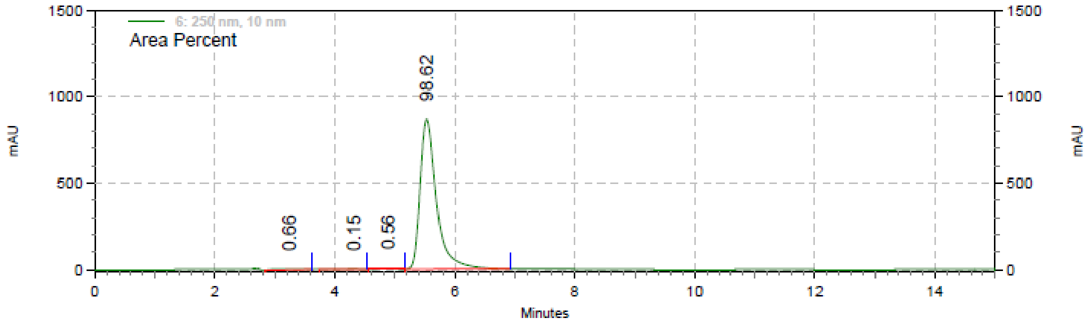

6: 250 nm, 10 nm Results

Pk #	Retention Time	Height	Height %	Area	Area %
1	3.23	12423	0.35	443014	0.66
2	4.31	6086	0.17	101654	0.15
3	4.88	20499	0.58	374062	0.56
4	5.53	3465346	98.89	65875893	98.62
**Totals**		3504354	100.00	66794623	100.00

Figure S44. HPLC data of FAP-MB-5.

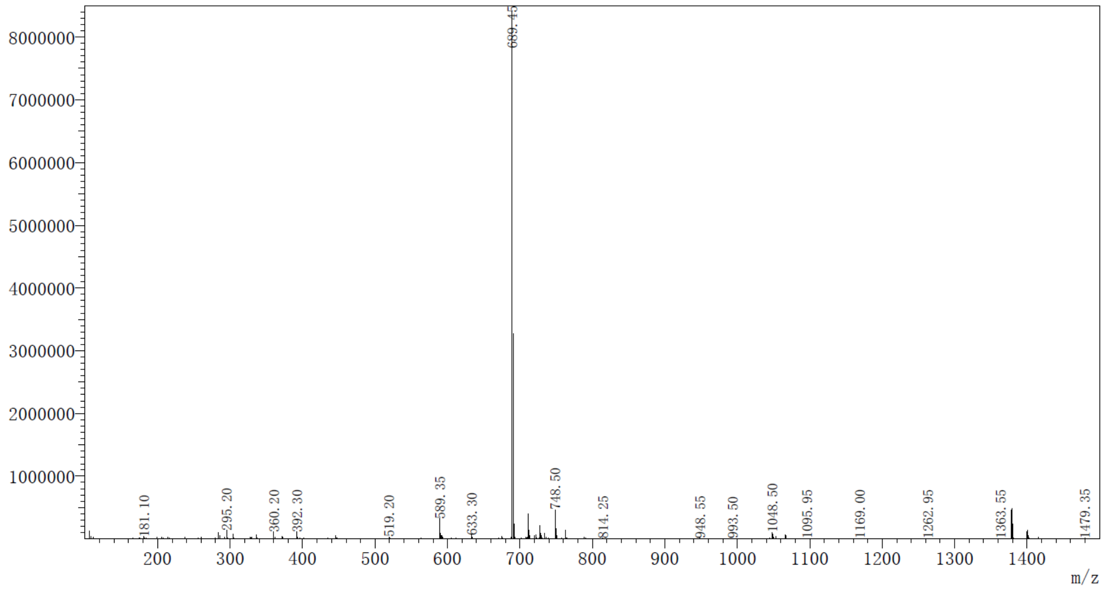

Figure S45. MS spectrum of FAP-MB-6.

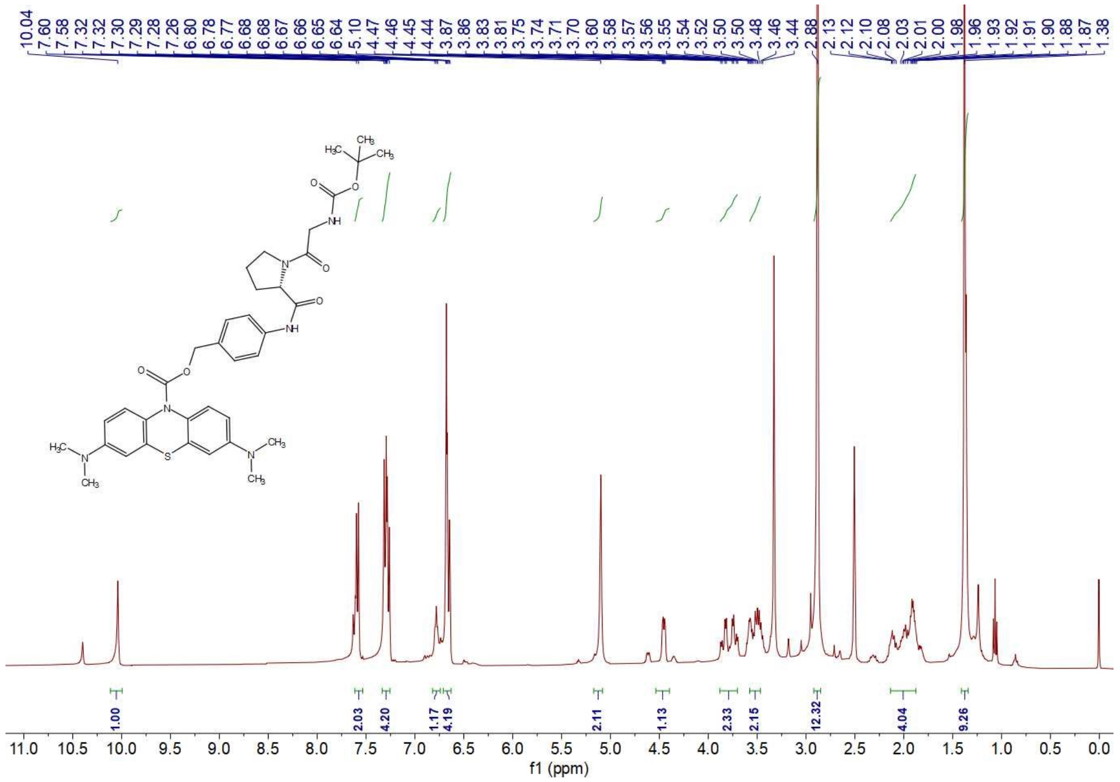

Figure S46. ^{1}H-NMR spectrum of FAP-MB-6.

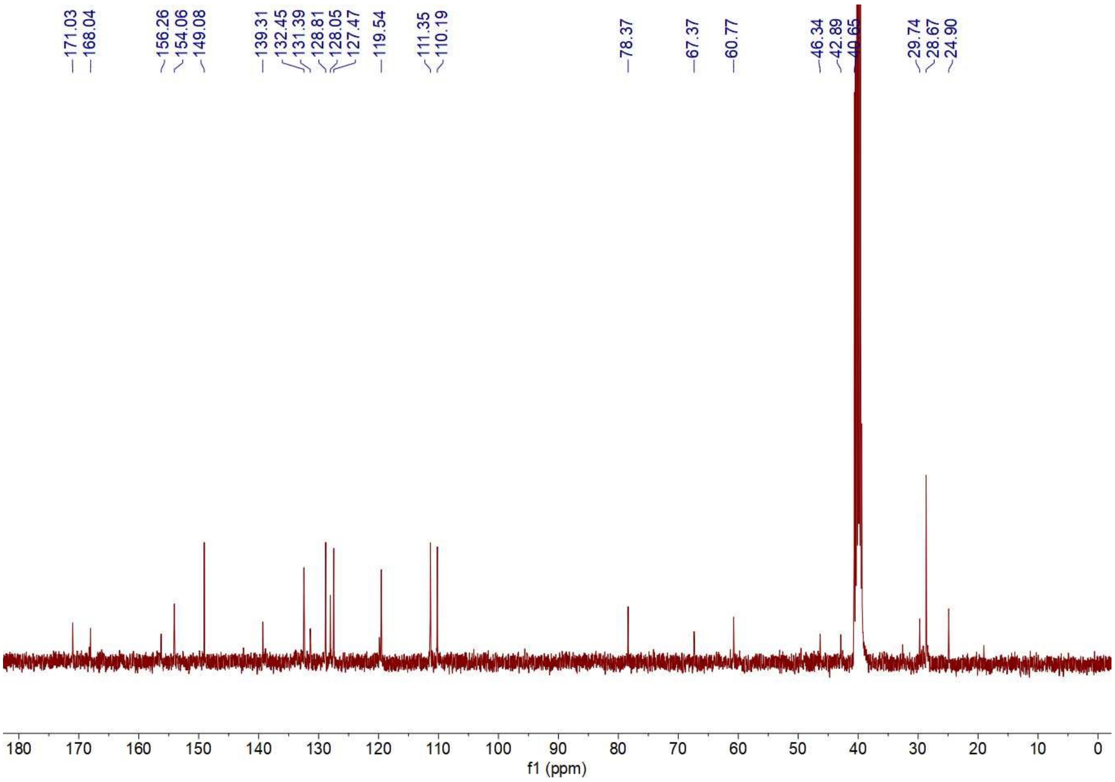

Figure S47. ^{13}C-NMR spectrum of FAP-MB-6.

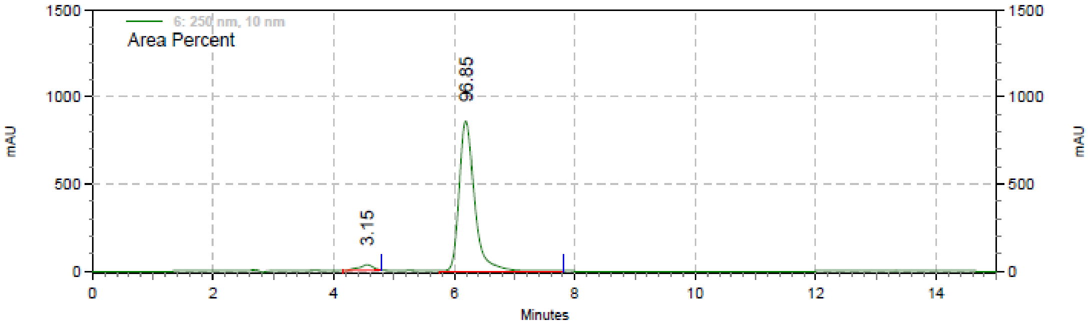

6: 250 nm, 10 nm Results

Pk #	Retention Time	Height	Height %	Area	Area %
1	4.55	125120	3.50	1964755	3.15
2	6.19	3446044	96.50	60430807	96.85
**Totals**		3571164	100.00	62395562	100.00

Figure S48. HPLC data of FAP-MB-6.

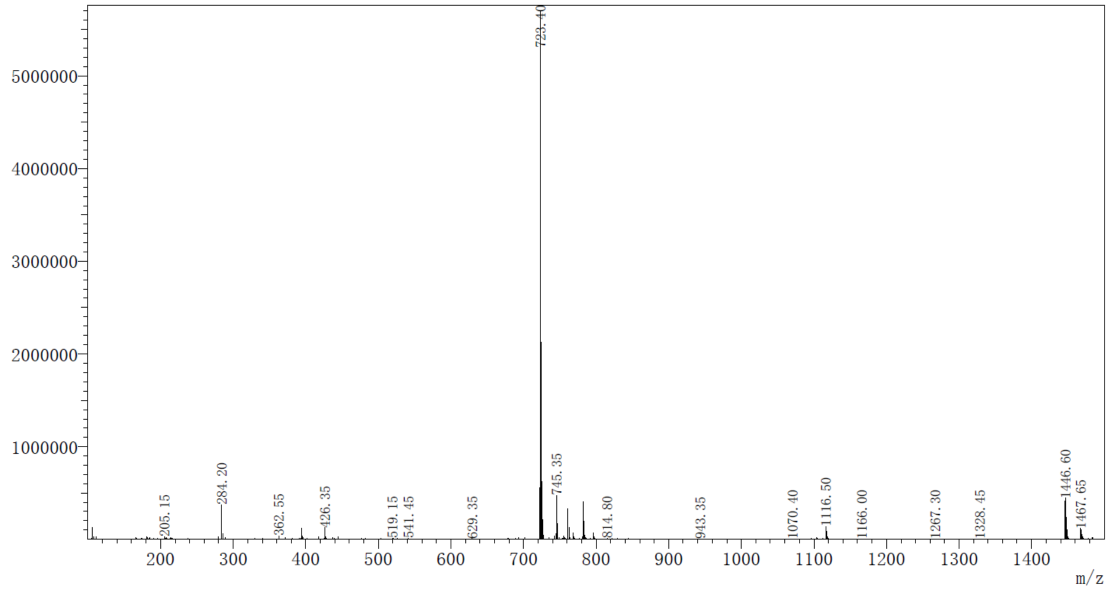

Figure S49. MS spectrum of FAP-MB-7.

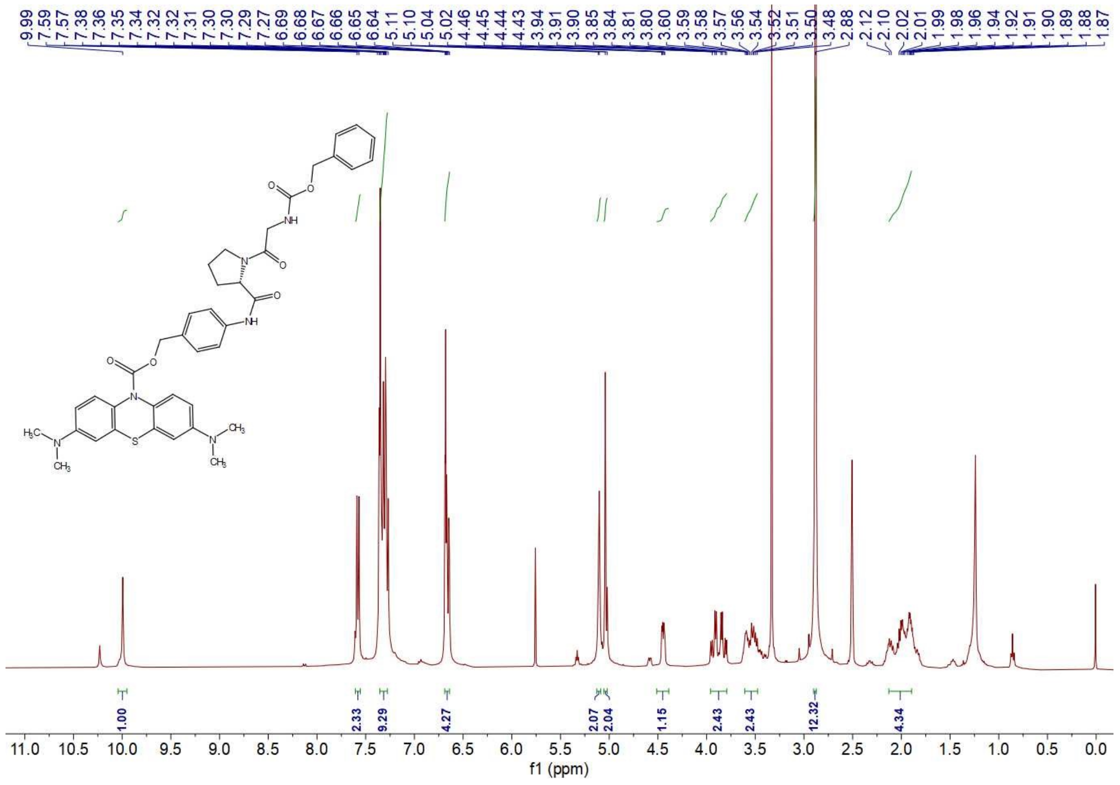

Figure S50. ^{1}H-NMR spectrum of FAP-MB-7.

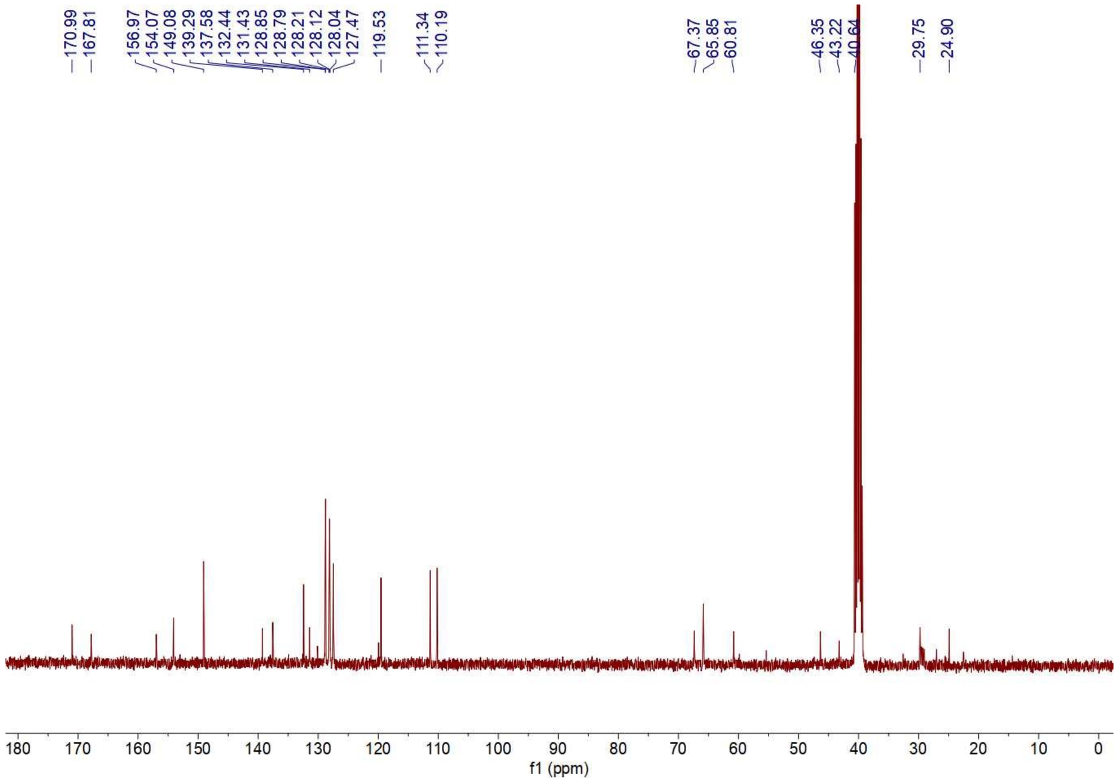

Figure S51. ^{13}C-NMR spectrum of FAP-MB-7.

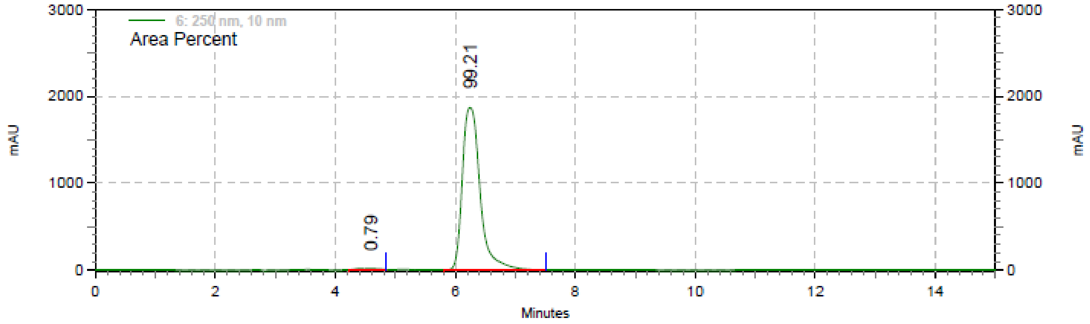

6: 250 nm, 10 nm Results

Pk #	Retention Time	Height	Height %	Area	Area %
1	4.59	52656	0.70	1207069	0.79
2	6.25	7470902	99.30	152062108	99.21
**Totals**		7523558	100.00	153269177	100.00

Figure S52. HPLC data of FAP-MB-7.

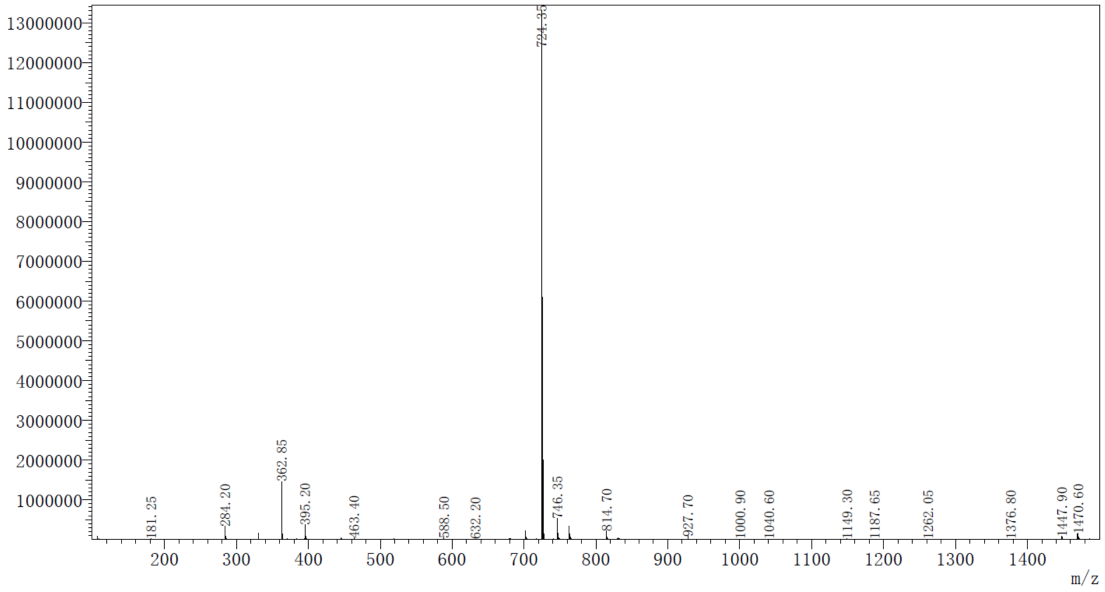

Figure S53. MS spectrum of FAP-MB-8.

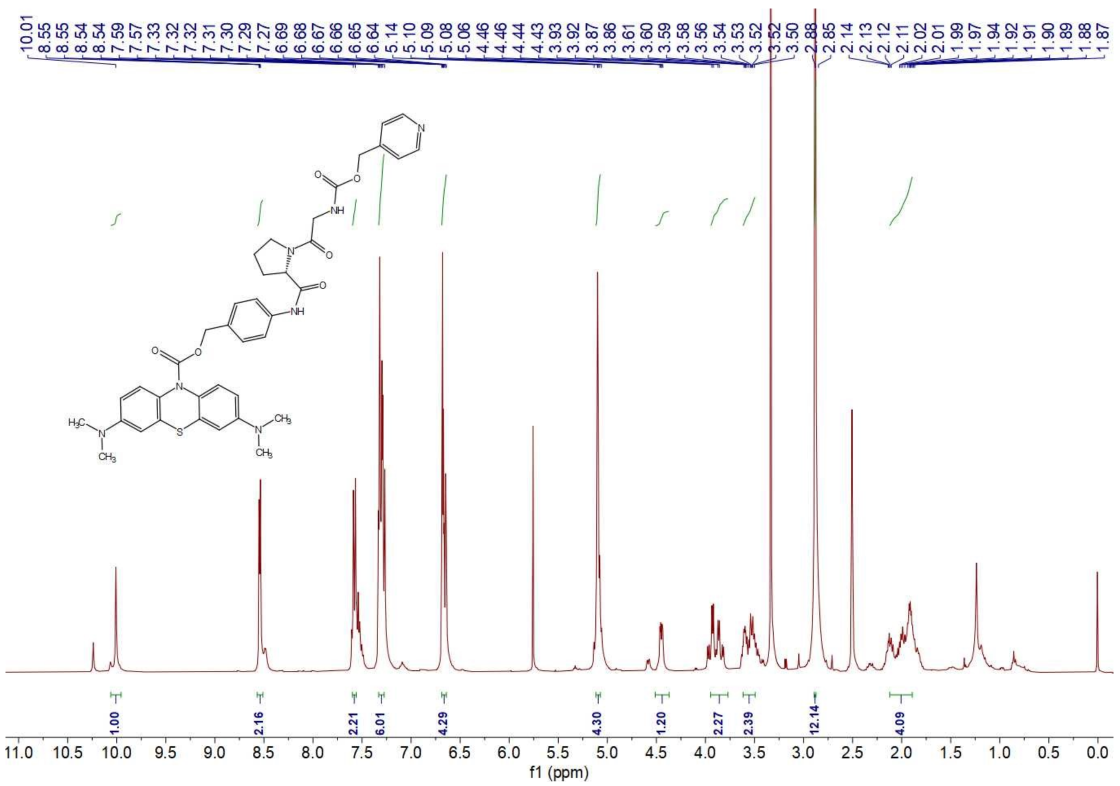

Figure S54. ^{1}H-NMR spectrum of FAP-MB-8.

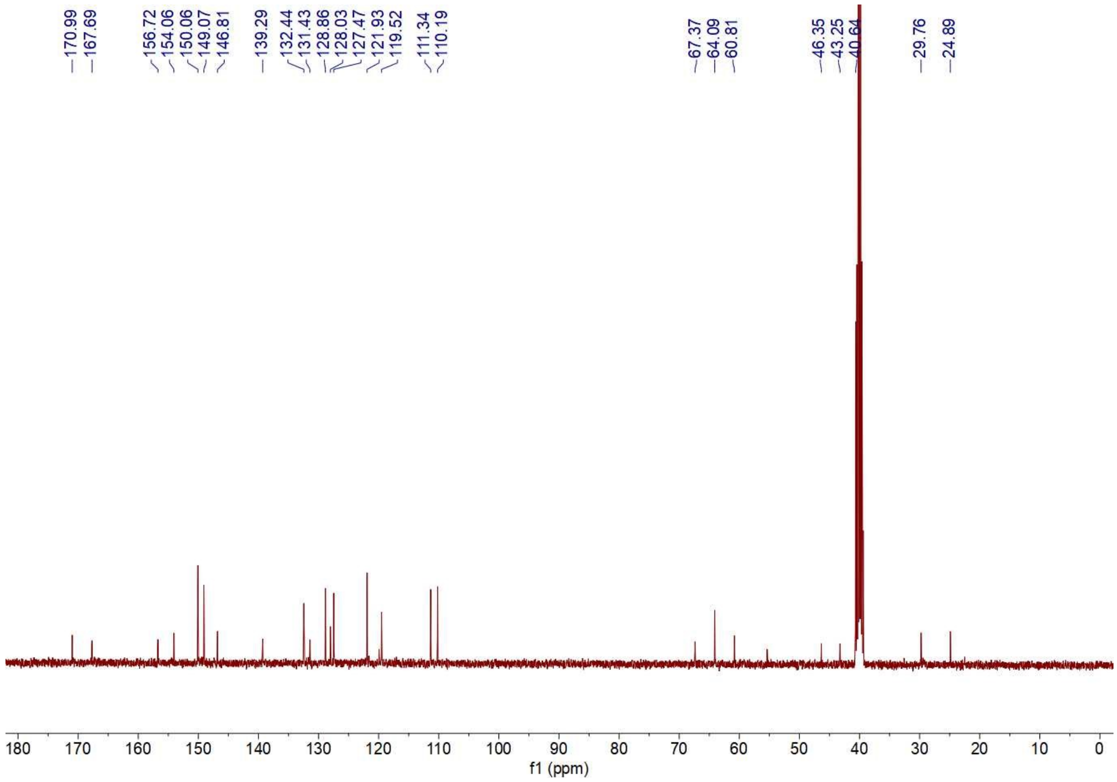

Figure S55. ^{13}C-NMR spectrum of FAP-MB-8.

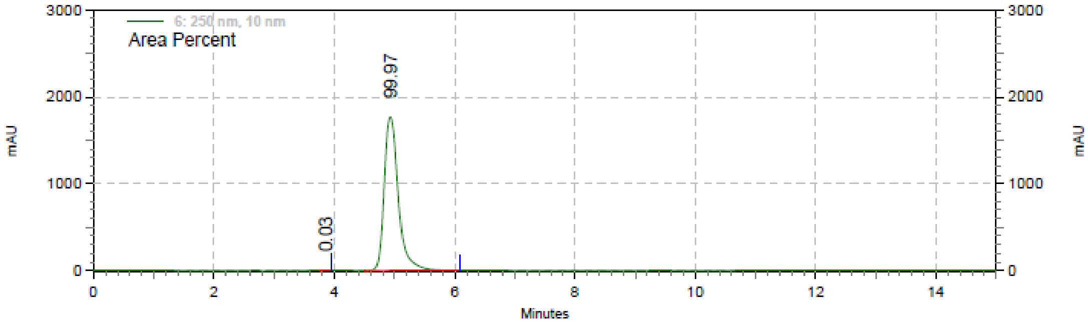

6: 250 nm, 10 nm Results

Pk #	Retention Time	Height	Height %	Area	Area %
1	3.85	5696	0.08	37911	0.03
2	4.93	7050536	99.92	115887300	99.97
**Totals**		7056232	100.00	115925211	100.00

Figure S56. HPLC data of FAP-MB-8.

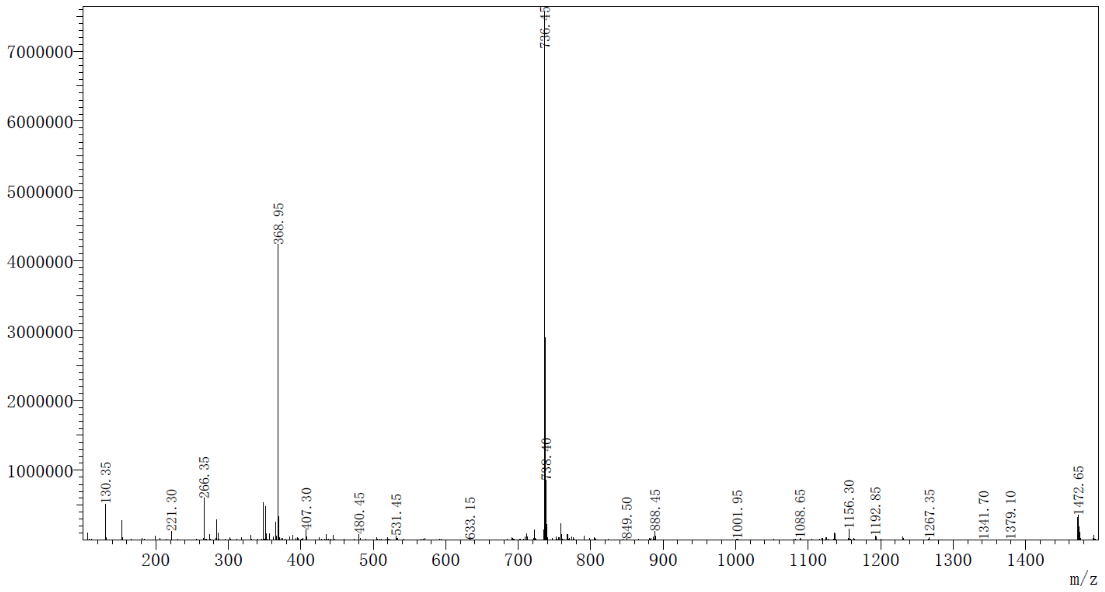

Figure S57. MS spectrum of FAP-MB-9.

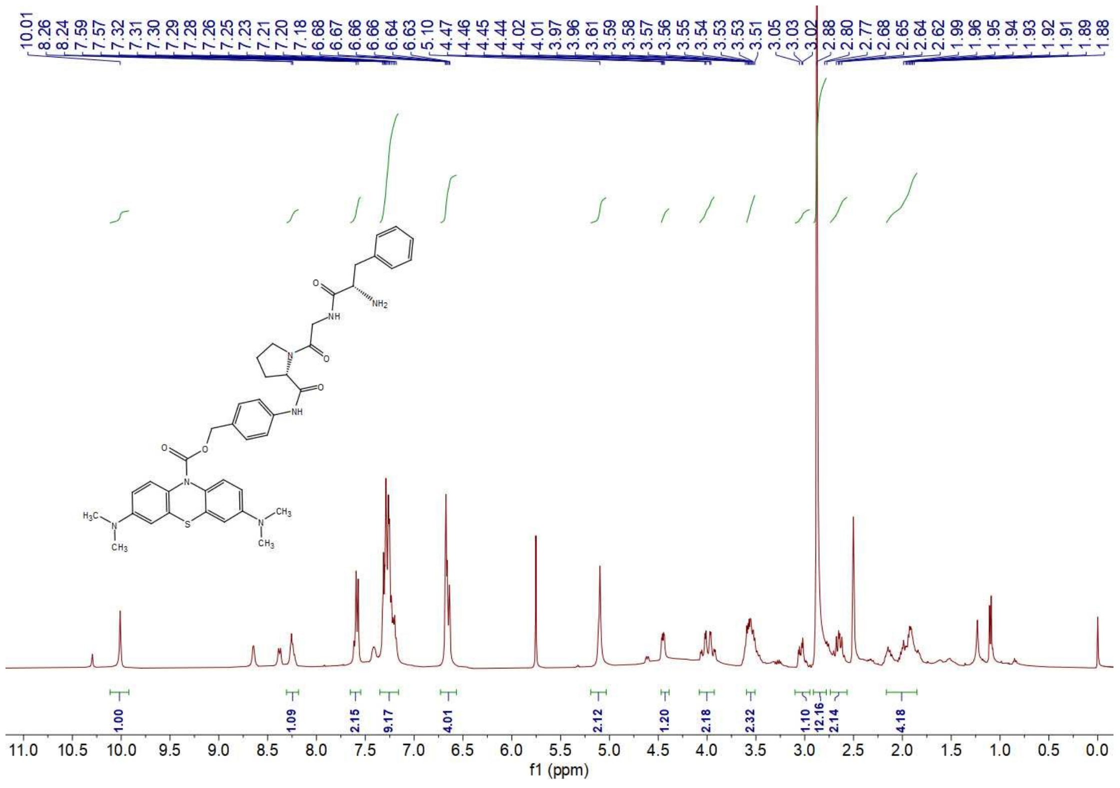

Figure S58. ^{1}H-NMR spectrum of FAP-MB-9.

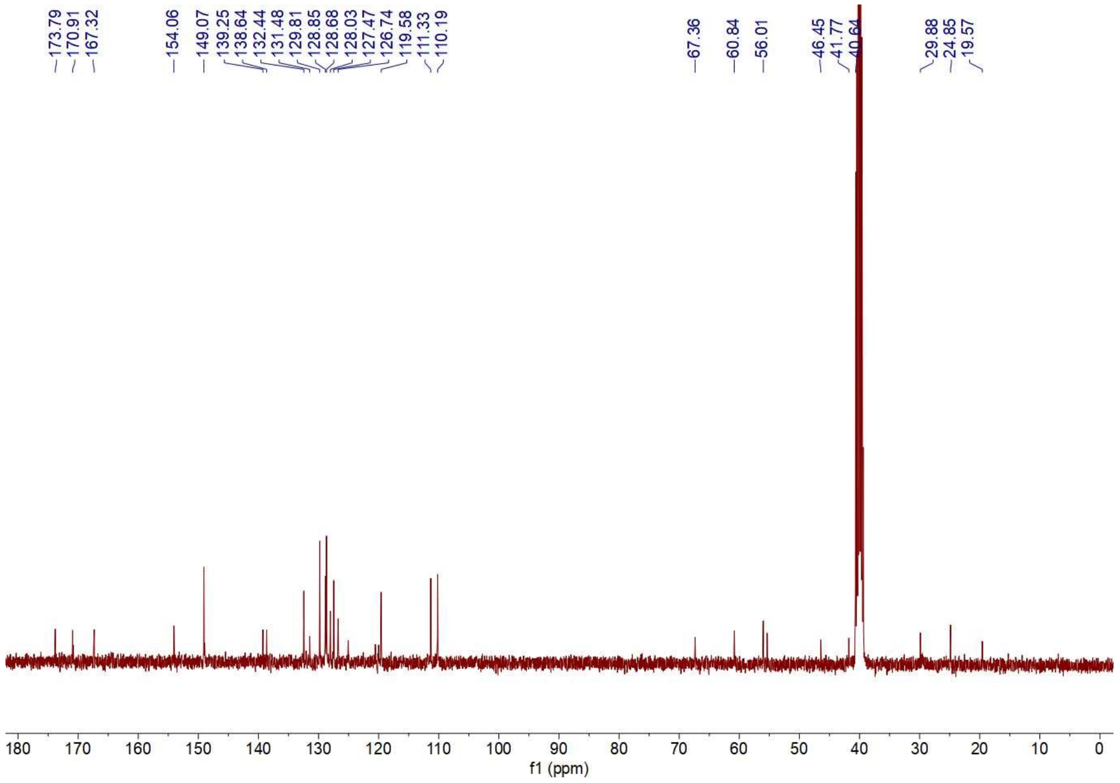

Figure S59. ^{13}C-NMR spectrum of FAP-MB-9.

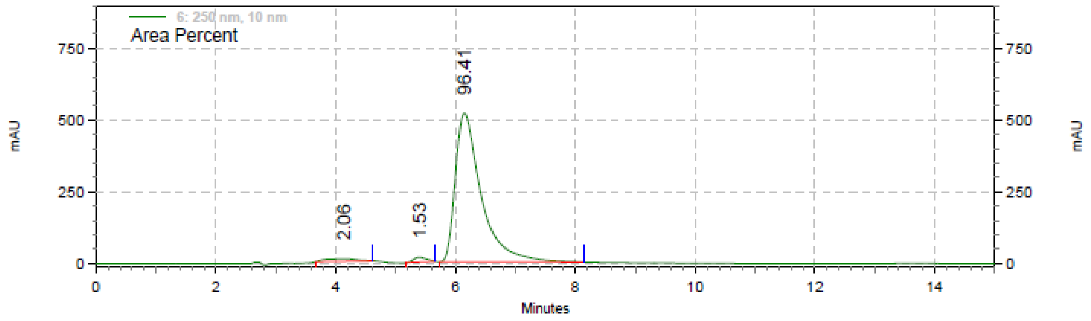

6: 250 nm, 10 nm Results

Pk #	Retention Time	Height	Height %	Area	Area %
1	4.13	37159	1.71	1328042	2.06
2	5.40	67453	3.10	986410	1.53
3	6.15	2074281	95.20	62119056	96.41
**Totals**		2178893	100.00	64433508	100.00

Figure S60. HPLC data of FAP-MB-9.

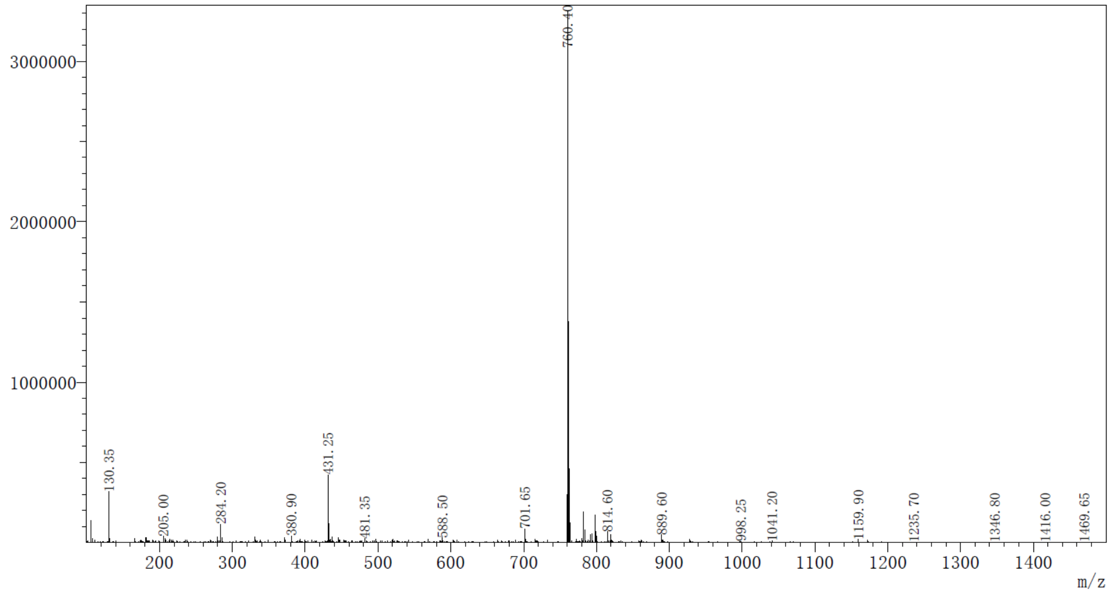

Figure S61. MS spectrum of FAP-MB-10.

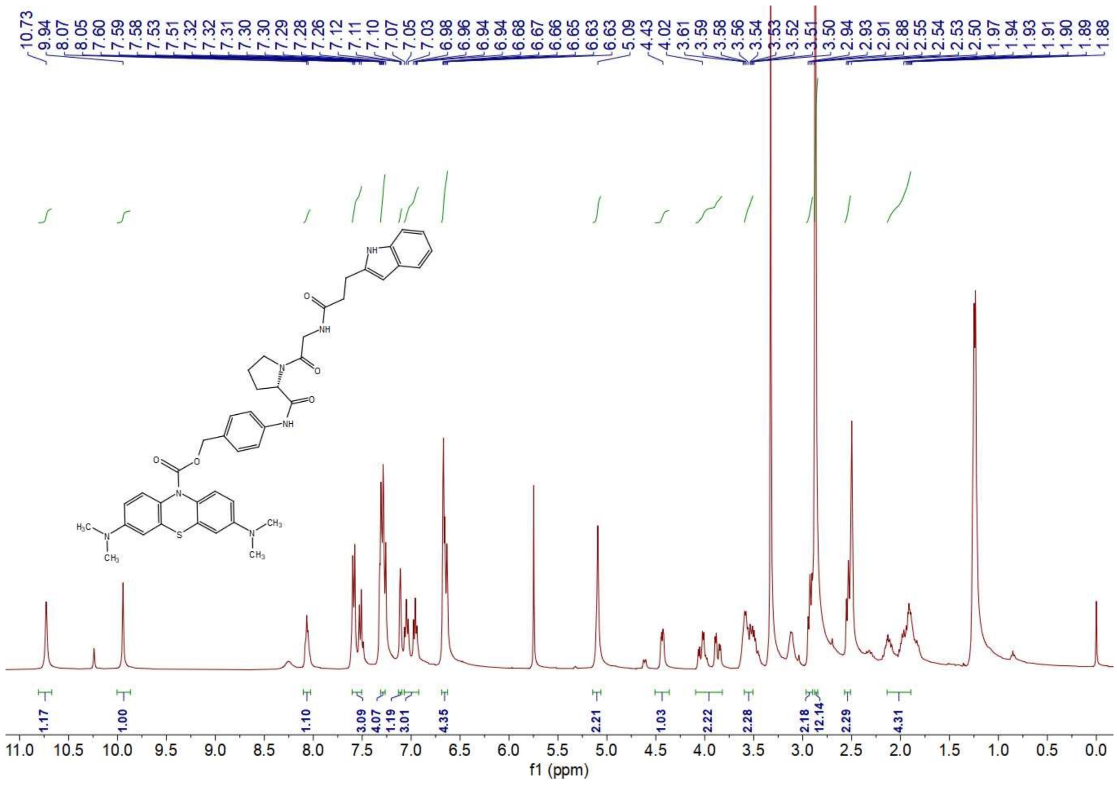

Figure S62. ^{1}H-NMR spectrum of FAP-MB-10.

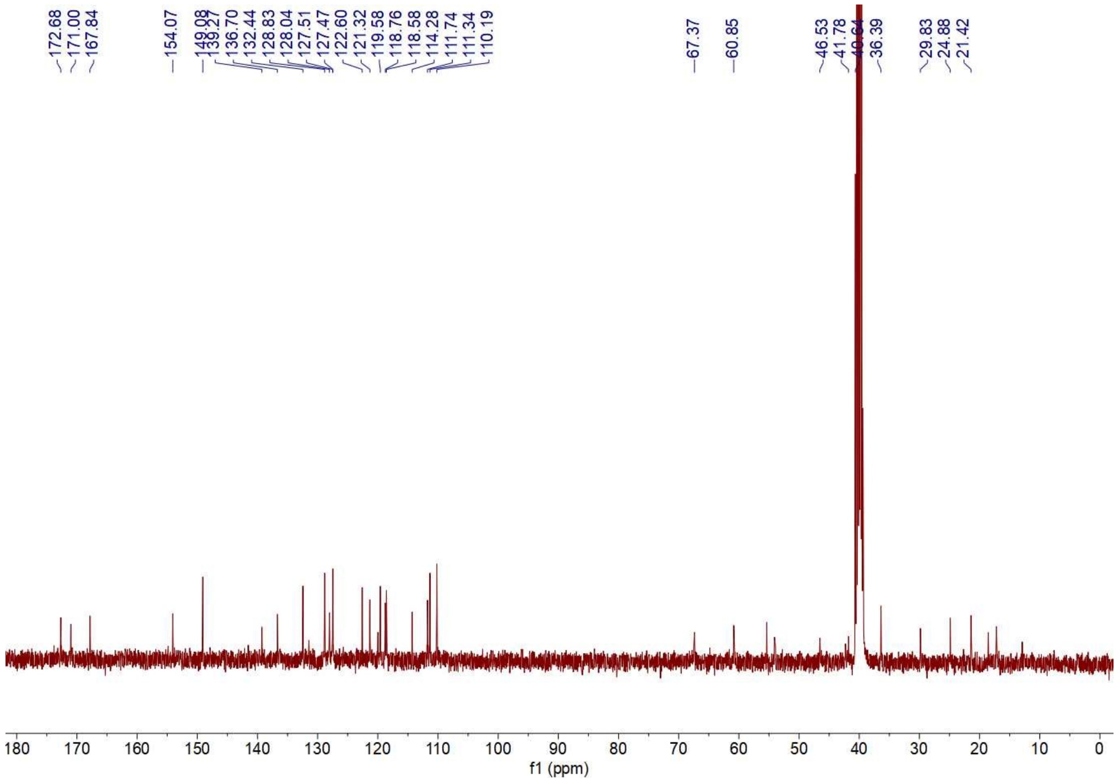

Figure S63. ^{13}C-NMR spectrum of FAP-MB-10.

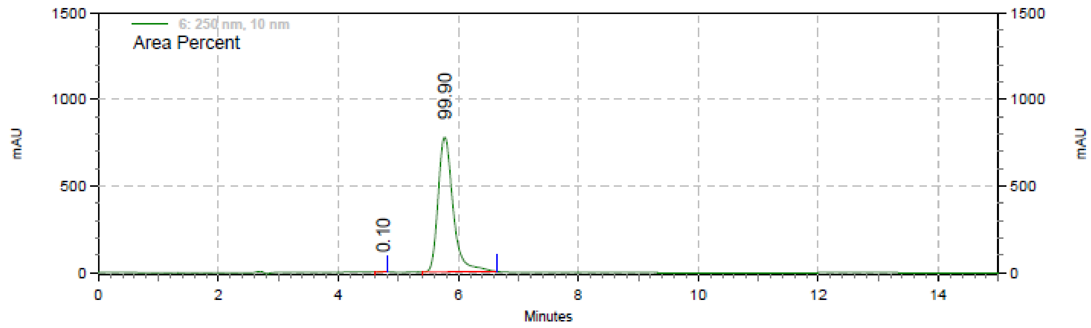

6: 250 nm, 10 nm Results

Pk #	Retention Time	Height	Height %	Area	Area %
1	4.74	7659	0.25	55358	0.10
2	5.77	3100329	99.75	53382603	99.90
**Totals**		3107988	100.00	53437961	100.00

Figure S64. HPLC data of FAP-MB-10.

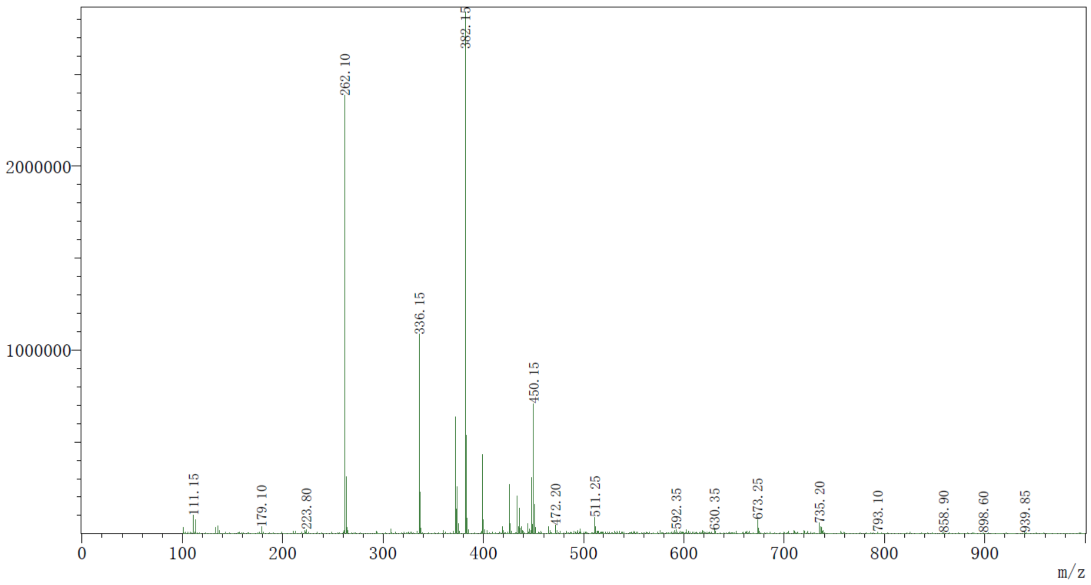

Figure S65. MS spectrum of compound B-2.

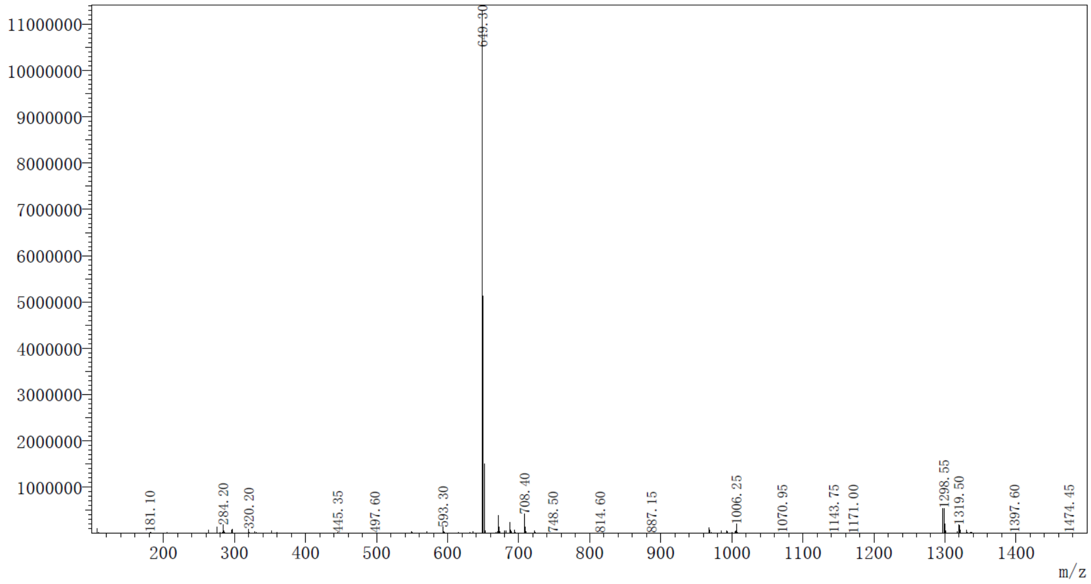

Figure S66. MS spectrum of compound C-2.

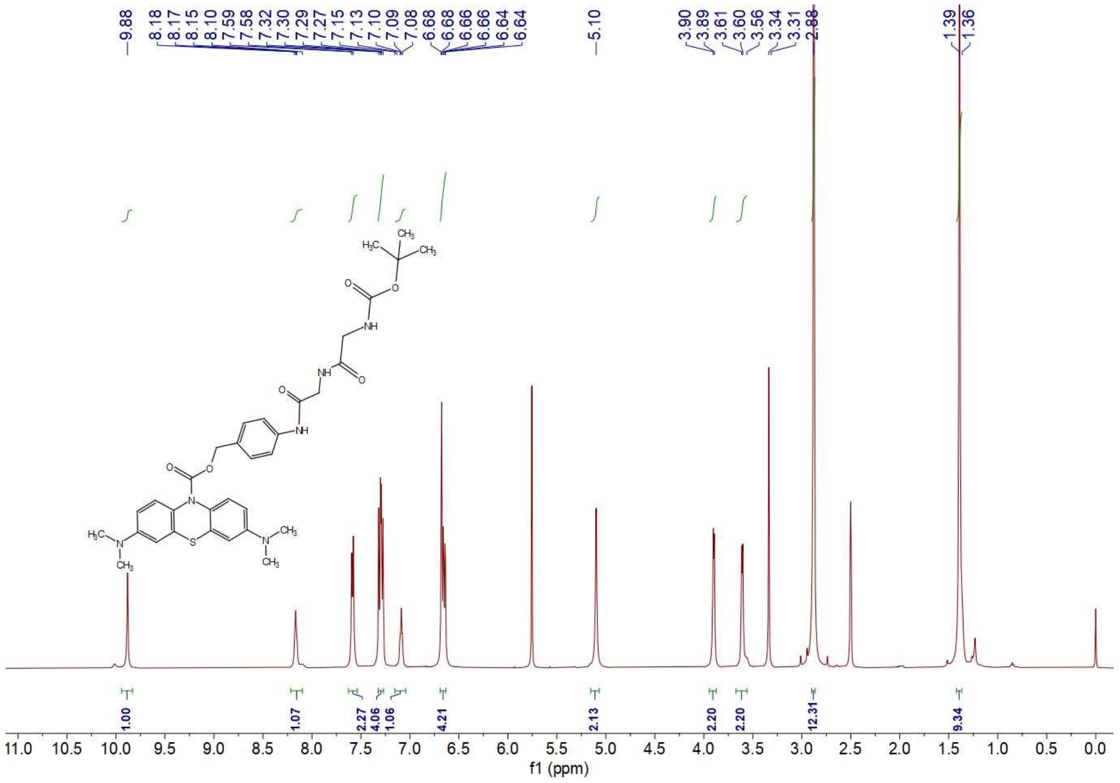

Figure S67. ^{1}H-NMR spectrum of compound C-2.

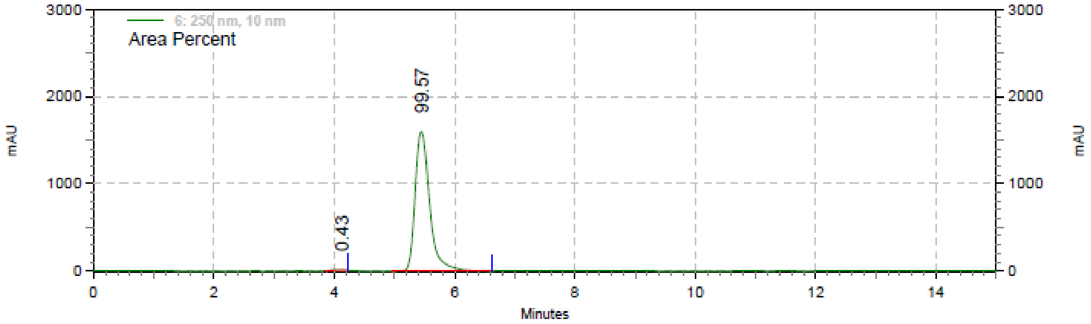

6: 250 nm, 10 nm Results

Pk #	Retention Time	Height	Height %	Area	Area %
1	4.13	32360	0.50	455151	0.43
2	5.45	6375789	99.50	105786796	99.57
**Totals**		6408149	100.00	106241947	100.00

Figure S68. HPLC data of compound C-2.

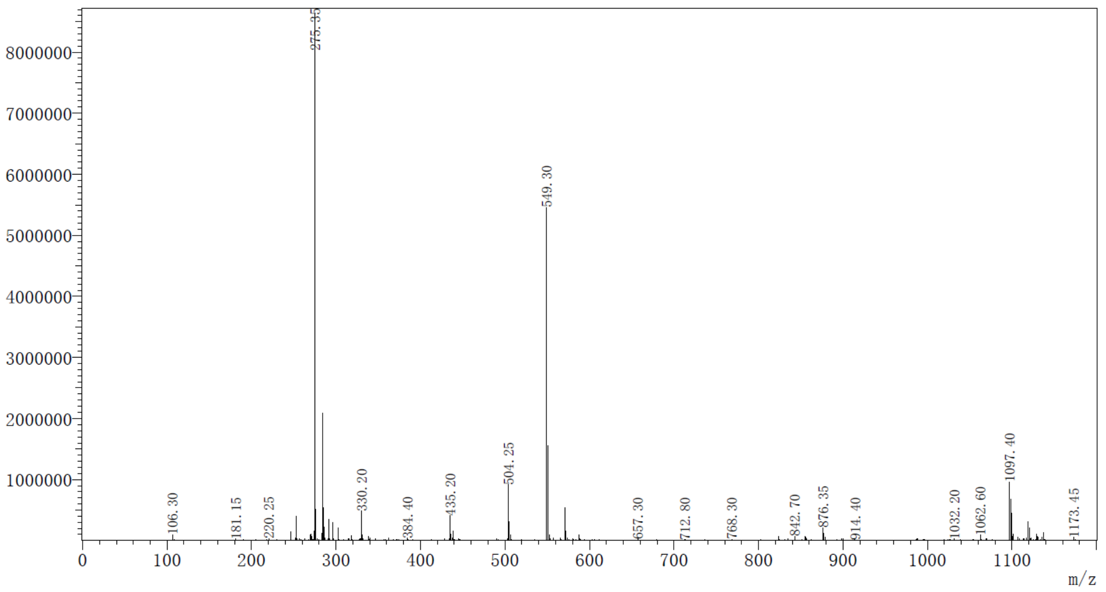

Figure S69. MS spectrum of compound D-2.

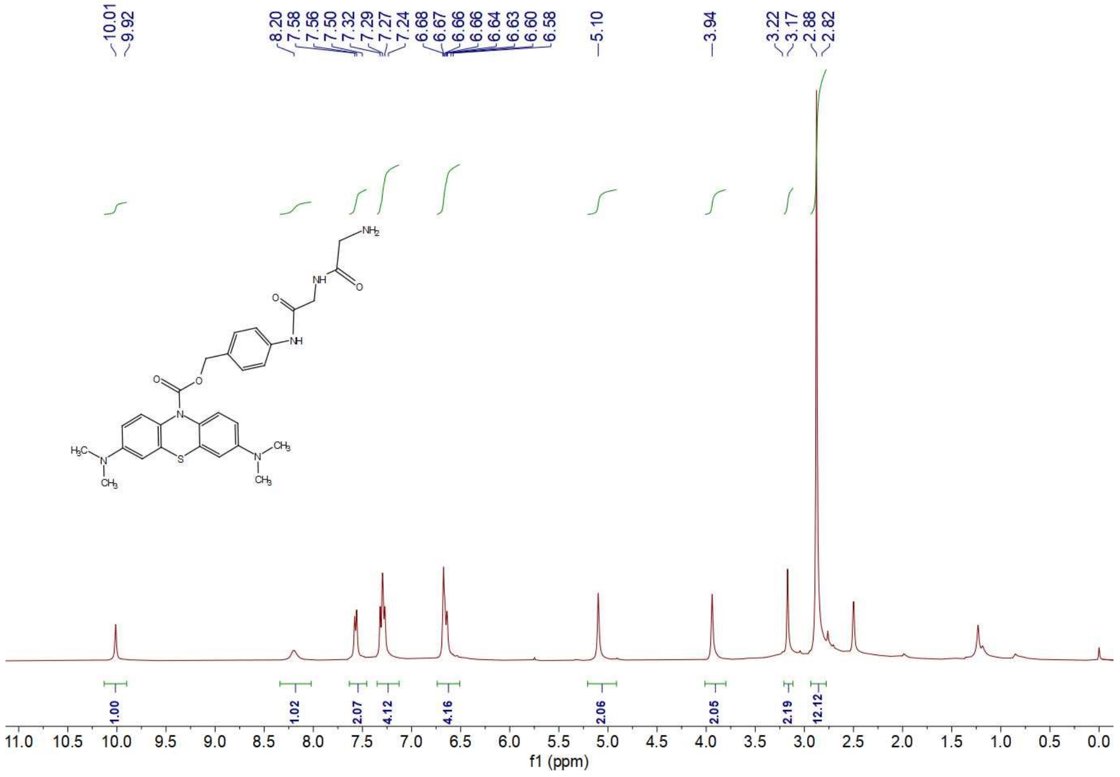

Figure S70. ^{1}H-NMR spectrum of compound D-2.

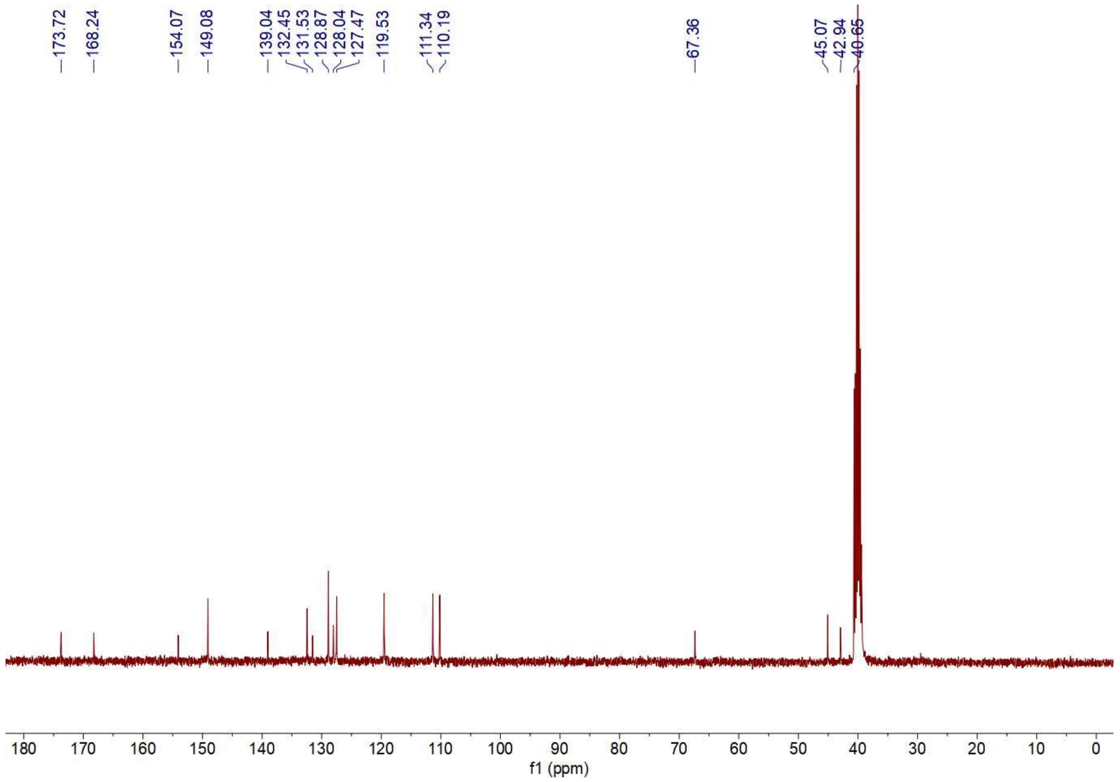

Figure S71. ^{13}C-NMR spectrum of compound D-2.

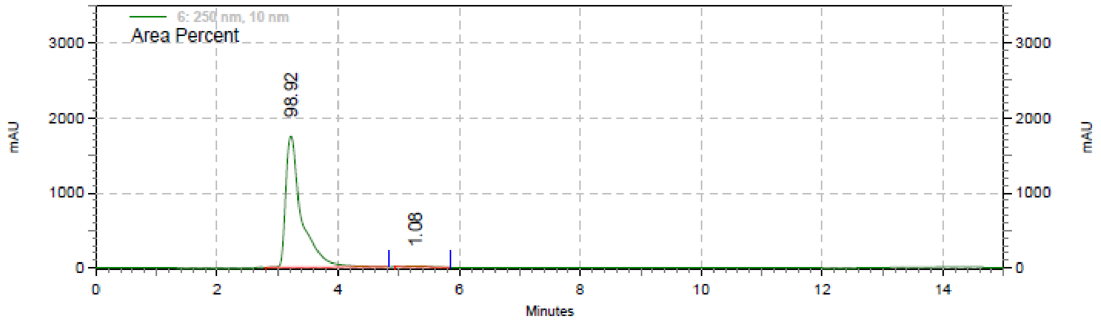

6: 250 nm, 10 nm Results

Pk #	Retention Time	Height	Height %	Area	Area %
1	3.22	6985738	99.37	127660448	98.92
2	5.27	44414	0.63	1387753	1.08
**Totals**		7030152	100.00	129048201	100.00

Figure S72. HPLC data of compound D-2.

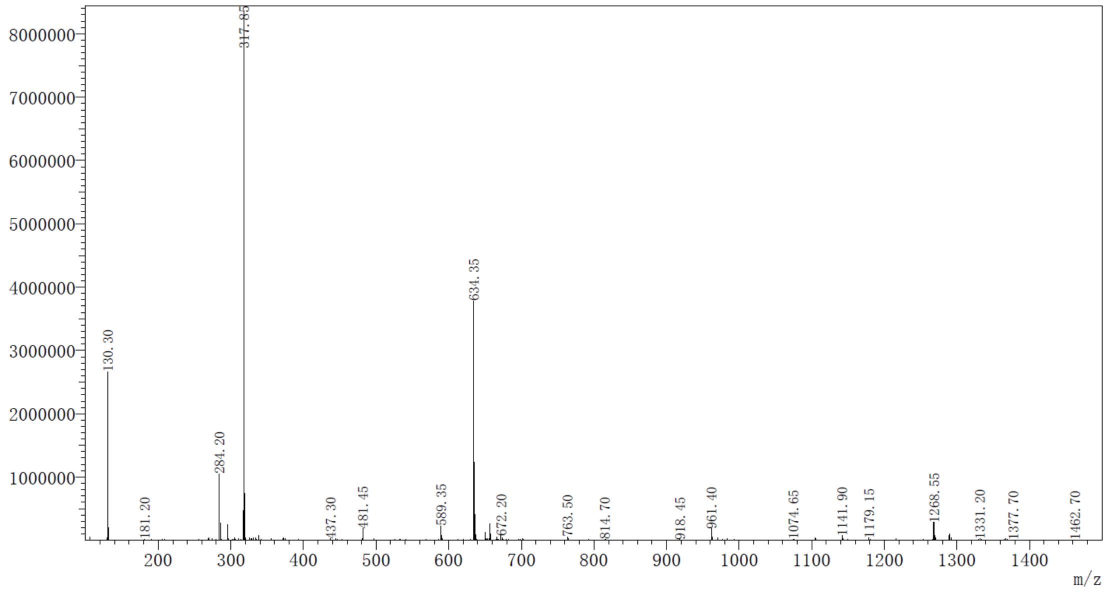

Figure S73. MS spectrum of N-FAP-MB.

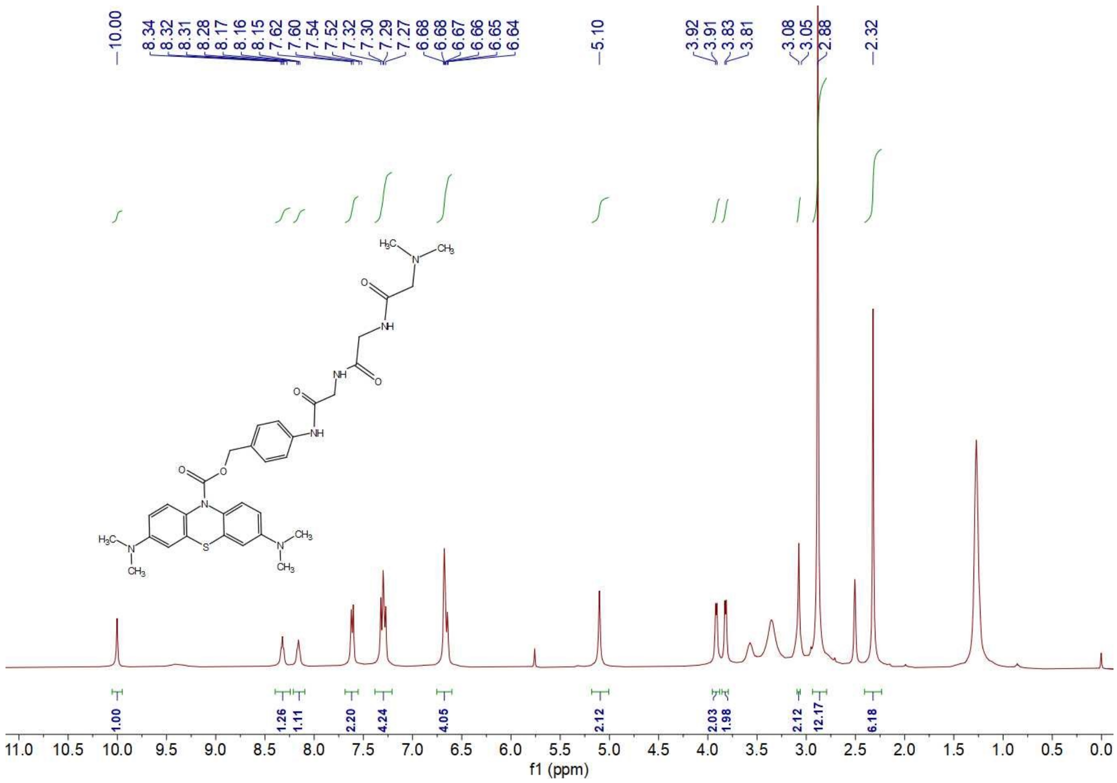

Figure S74. ^{1}H-NMR spectrum of N-FAP-MB.

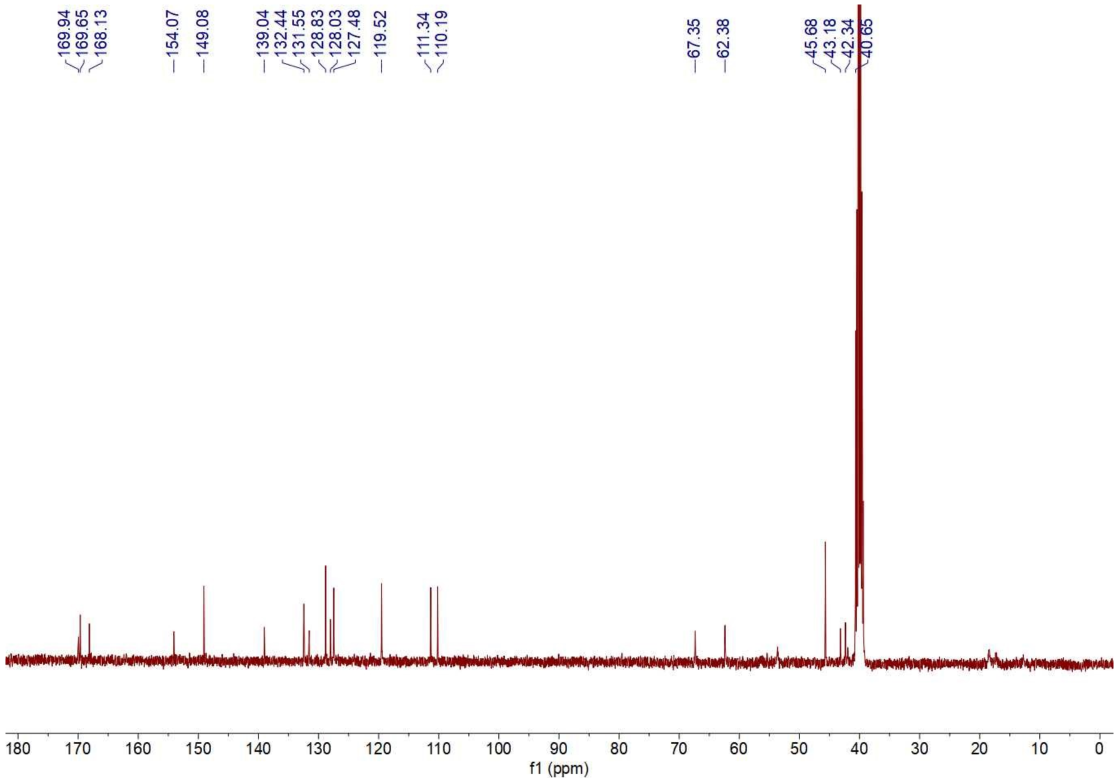

Figure S75. ^{13}C-NMR spectrum of N-FAP-MB.

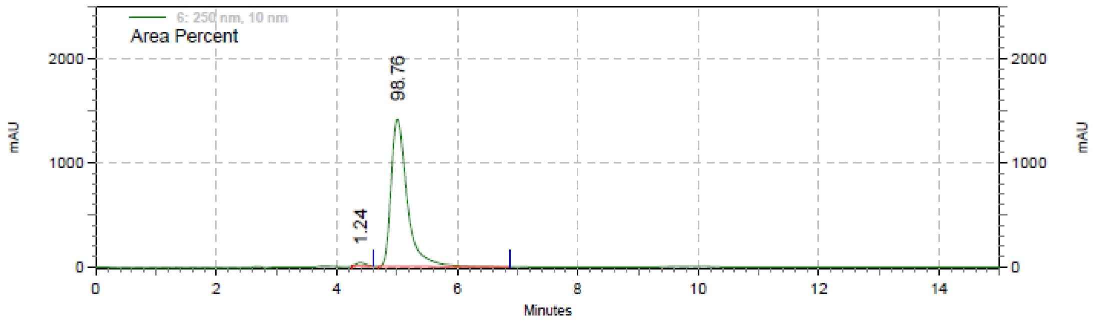

6: 250 nm, 10 nm Results

Pk #	Retention Time	Height	Height %	Area	Area %
1	4.39	119782	2.08	1312957	1.24
2	5.01	5630472	97.92	104399730	98.76
Totals		5750254	100.00	105712687	100.00

Figure S76. HPLC data of N-FAP-MB.
